# Cocaine- and amphetamine-regulated transcripts in two percomorphs: evolutionary conservation and energy-status dependent responses

**DOI:** 10.3389/fendo.2026.1870522

**Published:** 2026-06-30

**Authors:** Nikko Alvin R. Cabillon, Liat Koch, Noam Mizrahi, Shifra Ben-Dor, Adi Segev-Hadar, Iris Meiri Ashkenazi, Gertrude Alupo, Amir Bitan, Jakob Biran

**Affiliations:** 1 Department of Poultry and Aquaculture, Institute of Animal Science, Agricultural Research Organization, Rishon LeTsiyon, Israel; 2 Department of Animal Sciences, Robert H. Smith Faculty of Agriculture, Food and Environment, The Hebrew University of Jerusalem, Rehovot, Israel; 3 Israel Oceanographic and Limnological Research, The National Center for Mariculture, Eilat, Israel; 4 Marine Biology and Biotechnology Program, Department of Life Sciences, Ben-Gurion University of the Negev, Beer-Sheva, Israel; 5 Department of Life Sciences Core Facilities, Weizmann Institute of Science, Rehovot, Israel

**Keywords:** appetite, CART, neuroendocrine, seabream, tilapia

## Abstract

Cocaine and amphetamine-regulated transcript (Cart) is a pleiotropic neuropeptide involved in appetite regulation and maintenance of energy homeostasis, among other processes. Although Cart is widely studied in vertebrates, its multigenic nature in fish raises questions about which functions are affected by specific Cart peptides. This study compared two important aquaculture percomorph species with distinct habitats and feeding behaviors: Nile tilapia (*Oreochromis niloticus*) and gilthead seabream (*Sparus aurata*). Bioinformatics revealed seven *cart* genes in tilapia and six in seabream that show high sequence conservation with other vertebrates. Additionally, the predicted mature peptide sequences contain all the cysteines known to stabilize the tertiary peptide structure in other vertebrates. Nevertheless, protein structure modeling suggested that some carts had lost part or all of their cysteine-based disulfide bridges. Quantitative-PCR demonstrated that while all *cart*s are mainly expressed in the brain, several are also expressed in other tissues. Short- and long-term food deprivation resulted in decreased midbrain expression of specific *carts* in each species, while forebrain and hindbrain *cart* responses varied. Taken together, our findings demonstrate species-specific *cart* responses to fish energy status in the Nile tilapia and gilthead seabream, thus, supporting the possibility of functional partitioning in the piscine CART system.

## 1 Introduction

Feeding is regulated by a complex network of orexigenic (appetite-stimulating) and anorexigenic (appetite-suppressing) factors in the vertebrate central nervous system. One of the most potent anorexigenic neuropeptides is cocaine- and amphetamine-regulated transcript (Cart). Since its discovery as a psychoactive drug-specific (cocaine and amphetamine) upregulated transcript in the rat brain (1, 2), Cart has been implicated in diverse physiological processes including stress and anxiety, reward and addiction, reproduction, sleep, and olfaction (3–6). Nevertheless, Cart is mostly known for its role in the central regulation of appetite and feeding (3, 7–10). Several receptors have been suggested to function as cognate receptors for Cart, including CB1 and GPR160 (11–13). Recently, GPR162 has been identified as the Cart receptor in beta cells (14), yet Cart remains an orphan ligand.

In mammals, preproCart and its resulting peptides originate from a single *cart* gene (15, 16). However, in other vertebrates such as birds, amphibians, and fish, multiple *cart* genes have been identified, probably due to various events of whole genome duplication (WGD) in ancestral vertebrates, fish-specific WGDs, and lineage-specific gene losses (17–24). Nevertheless, Cart sequences in vertebrates are highly conserved, especially in the carboxy-termini of preproCart that yield its bioactive forms (25, 26). A number of alternative processing sites and posttranslational modifications give rise to various bioactive Cart peptides, which were correlated with diverse physiological and metabolic changes (27–30). Cart potency as a feeding suppressor was suggested to depend on the stringency of the disulfide bonds formed by the three pairs of cysteine residues in the bioactive peptide (30).

*cart* expression in the rat hypothalamic arcuate, paraventricular and ventromedial nuclei (31), the peripheral nervous system (32), and peripheral tissues such as the gastrointestinal tract, adrenal glands and pancreatic islets (33–36) further support pleiotropic functions of this peptide. Anorexigenic effects of the cart peptide were demonstrated in several fish species (17, 37–40), yet as piscine genomes contain multiple *cart* genes, the possibility of sub-functionalization and/or neo-functionalization of specific *cart*s is possible. Hence, studying the role of cart in regulating fish feeding and appetite requires identifying cart genes in the species studied.

Nile tilapia (*Oreochromis niloticus*) is a freshwater omnivore native to tropical and subtropical Africa (41, 42), while the gilthead seabream (*Sparus aurata*) is a marine species that is mainly carnivorous, common in the Eastern Atlantic and the Mediterranean (43, 44). Both species are highly important for aquaculture production. Therefore, understanding the mechanisms underlying feeding regulation in these species is valuable for comparative neuroendocrine research and for improving aquaculture practices.

In the current study, we identified multiple *cart* genes in the genomes of Nile tilapia and gilthead seabream. Next, we performed an extensive bioinformatics screen for putative cart sequences in various vertebrata genomes, including representatives of jawless vertebrates. Phylogenetic analysis revealed that these cart sequences cluster into four distinct clades, with tilapia and seabream carts represented in all clades. Combined with tertiary structural predictions, our findings suggest that while all *cart* peptides retained six stabilizing cysteines, some disulfide bridges were lost. Additionally, while all *cart* genes are primarily expressed in the midbrain, some cart genes also show high expression in other brain compartments and peripheral tissues, supporting their involvement in various physiological functions. To further study the anorexigenic nature of the identified *cart* genes, tilapia and seabream were subjected to short- or long-term food deprivation, and cart gene expression was analyzed by quantitative PCR. We found that only specific *cart* genes responded to either or both challenges, and that while the expression of some *cart*s suppressed by short- or long-term food deprivation, expression of other *cart*s increased in response to these challenges. Our current findings support the presence of anorexigenic cart genes in each species and suggest functional partitioning in the piscine cart system.

## 2 Methods

### 2.1 Bioinformatics data mining

Seven *cart* orthologs have been previously predicted from the Nile tilapia genome (18), and one cloned gilthead seabream *cart* gene (45). These sequences were used for Translated Basic Local Alignment Search Tool (tblastn) package (NCBI, http://www.ncbi.nlm.nih.gov/BLAST) (46) searches against whole-genome shotgun contigs of gilthead seabream.

Additional animal genomes were selected and searched for to give a large evolutionary spread. TBlastN searches were performed against the most recent RefSeq version of each genome. If a blast hit overlapped a defined protein, that sequence was taken. If no defined protein was available or the prediction was deemed not reliable, manual curation was done, and the sequences were predicted. The putative protein sequences were tested for the presence of the CART domain using CDsearch. All sequences had either cl05722: CART_CTD-like superfamily or pfam06373: CART hits, except for non-Craniata species (tunicates and lancelets). An HMMscan (https://www.ebi.ac.uk/Tools/hmmer/search/hmmscan) was performed against the Pfam database (v37.2) on the distant sequences, and with relaxed parameters, hits were found to pfam06373: CART.

### 2.2 Phylogenetic analysis

Due to the variability of the first exon, the predictions were considered less reliable, and only the second and third exons (which contain the functional elements, KK/KR in the second exon and 6C in the third) were taken for final alignment and tree construction. Alignments were performed with Muscle 3.8.31 and ClustalW v2.1. The alignments were consistent, and the Muscle alignments were used for the trees. Trees were constructed using Phyml 3.0 (http://www.atgc-montpellier.fr/phyml/) online with the default parameters. Trees were visualized with iTol v7 (itol.embl.de/). Logos were constructed with Weblogo 3.9.0 (https://weblogo.threeplusone.com/). Bioinformatics analyses were performed during February 2026. Sequences and alignments are provided as **Supplementary Files 1, 3, 4**.

### 2.3 Cloning

Total RNA was extracted using Trizol reagent (Life Technologies Corporation, Carlsbad, USA) for tilapia or Tri-reagent (BioLab, Jerusalem, Israel) for seabream, according to manufacturer’s protocols. Total RNA quantity and purity were measured using a microplate spectrophotometer Epoch™ for tilapia (BioTek instruments Inc. Winooski, USA) or by NanoDropOne (Thermo scientific Paisley, UK) for seabream. RNA integrity was assessed by running 1 microgram (µg) of total RNA on 1.5% agarose (LifeGene, Modi’in, Israel) containing Redsafe™ stain (Intron Biotechnology, Korea) in 1 x TAE (Tris-acetate acid-EDTA) buffer (Biological industries, Kibbutz Beit-Haemek, Israel). Possible genomic DNA contamination was eliminated by treatment with TURBO DNAse-free™ kit Invitrogen for tilapia (Thermo Fisher Scientific, Vilnius, Lithuania) or DNase (PerfeCTa^®^ DNase I RNase-free Quantabio, Beverly, USA) for seabream according to the manufacturer’s protocol. For tilapia, cDNA was reverse transcribed from 500 ng Total RNA using High Capacity cDNA Reverse transcription kit (Thermo Fisher Scientific, Vilnius, Lithuania), while for seabream 1 ug total RNA was reverse transcribed with qScript cDNA Synthesis Kit (Quantabio, Beverly, USA). Specific primer pairs used for both species are presented in (**Supplementary Table 6**). PCR products amplified with DreamTaq Green PCR Master Mix (Thermo Fisher Scientific) for tilapia or with GoTaq^®^ Green Master Mix (Promega Wisconsin, USA) for seabream were analyzed on 1% agarose (Life Gene, Modi’in, Israel) containing Redsafe™ stain (Intron Biotechnology, Korea) or GelRed^®^ Nucleic Acid Stain (SigmaAldrich Massachusetts, USA) in 1 x Tris-acetate acid-EDTA buffer (Biological industries, Kibbutz Beit-Haemek, Israel). PCR and qPCR products of the predicted amplicon size were extracted from the gel, cloned into pGEM-T easy vector (Promega, Wisconsin, U.S.A.), and sequenced using the forward primer or T7 primer at Hy Laboratories Ltd. (Rehovot, Israel).

### 2.4 Tertiary structure analyses and protein structure prediction

Based on the cloned *cart* transcript sequences, amino acid (aa) sequences were predicted using ExPASy (47). Multiple sequence analysis of CART peptides was done using MUSCLE (48). Tertiary protein structures of the mature *cart* peptide sequences were predicted using i-TASSER (49) and visualized using Jmol (50). The mature CART peptide is the most conserved region, and crystallography data had already been published for CARTPT (PDB entry: 1hy9; accession: Q16568) (51). The C-score, which is a confidence metric used to assess the quality of predicted models generated, was also calculated by i-TASSER. It is derived from the significance of threading template alignments and the convergence parameters of structure assembly simulations. The C-score typically ranges from -5 to 2, with higher values indicating greater confidence in the predicted model and lower values suggesting lower confidence (**Supplementary Table 5**).

### 2.5 Animal procedures

**Figure 1:**
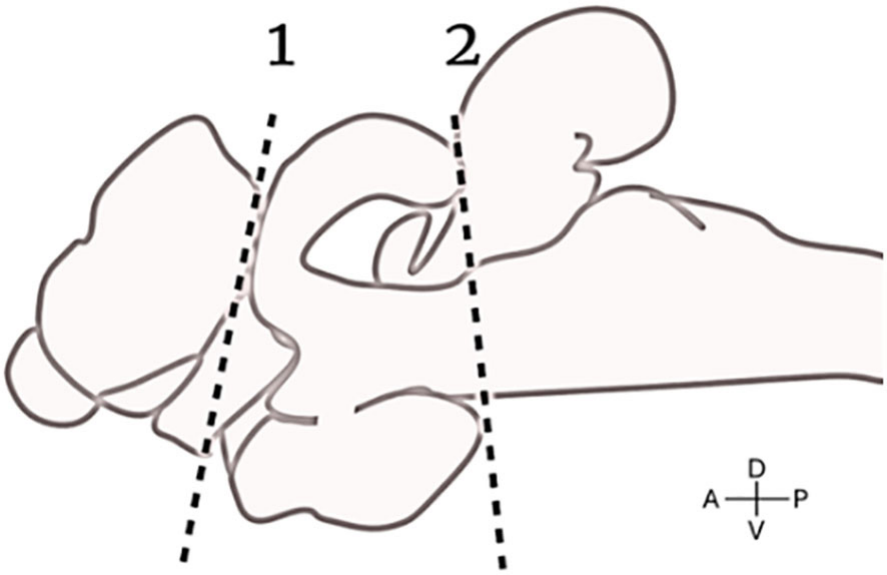
Dissection scheme of tilapia and seabream brains. The forebrain compartment contained the telencephalon and olfactory bulb. The midbrain compartment contained the diencephalon, midbrain tegmentum, tectum and valvulae cerebelli. The hindbrain compartment contained the rhombencephalon, cerebellum, and vagal lobe. lines. The cross at the bottom right indicates brain orientation. A – anterior; P – posterior; D – dorsal; V – ventral.

The experiments were approved by the Agricultural Research Organization (A.R.O.) Committee for Ethics in Using Experimental Animals (approval number: 877/20). Samples for tissue distribution in Nile tilapia were taken from (52). Briefly, we used adult males displaying running milt and weighing 81.87 ± 6.6 g, adult females with fully developed ovaries and weighing 27.13 ± 7.2 g. Samples for tissue distribution in adult seabream males (1035.00 ± 258.0 g) or females (1380 ± 260.6 g) were collected from fish reared under natural photoperiod, with continuous seawater supply, at a natural temperature and salinity. Fish were fed *ad libitum* with commercial dry pellets (6mm Raanan feed, Israel) containing 45% protein. Fish were euthanized using clove oil followed by decapitation. The brain was harvested and dissected into three major compartments (**Figure 1**). The forebrain compartment contained the telencephalon and olfactory bulb. The midbrain compartment contained the diencephalon, midbrain tegmentum, tectum, and valvulae cerebelli. The posterior compartment contained the rhombencephalon, cerebellum, and vagal lobe. The heart, liver, foregut, hindgut, white muscle, gills, testis or ovary, stomach, and kidney tissues were also harvested, snap-frozen in liquid nitrogen, and stored at -80°C until further analysis. Samples following short-term (SD) or long-term (LD) food depravation were collected as follows: Tilapia (*Oreochromis niloticus*) samples for LD analyses were taken from the study of Segev-Hadar et al. (52). The setup consisted of two treatments: one was fed twice daily, and the other was not fed for 21 days. For SD analyses, adult male tilapia (77.93 ± 2.6 g) were acclimated in experimental tanks for 2 weeks prior to the experiment. During acclimation, all fish were fed twice daily *ad libitum* with commercial tilapia feed containing 33% protein (Zemach Feed Mill, Israel). Temperature was maintained at 26˚C, and physico-chemical parameters were monitored. The SD group was harvested 24 h post last feeding based on a previous study demonstrating that significant metabolic changes in Nile tilapia already occur after 24 h of food deprivation (53). The control-fed group was maintained under a regular regime and harvested 1 h after the last feeding. For seabream (*Sparus aurata*), fish (92.37± 6.06 g) were acclimated for 9 days in sea water (with salinity of about 40‰) open flow through tanks (270L), under natural ambient temperature in the range of 22-24˚C and divided for 3 treatments as follows: fed fish that were fed twice a day (Raanan food, Israel) with food containing 46% protein *ad libitum*, or fish that were food deprived for 48 h (SD) or 7 days (LD) prior to sampling. Previous studies in seabream indicate that 24 h of feed deprivation was too short to induce metabolic changes, whereas 48 h were sufficient to induce a hyperphagic response (54, 55), and in 7 days, long-term energy stores were almost completely depleted (56). Each treatment consisted of 3 tanks.

### 2.6 Quantitative real-time PCR

cDNA from various tissues was prepared as described above and stored at -20^0^C. Expression levels were analyzed by quantitative real-time PCR (qPCR) using a StepOnePlus™ Real-Time PCR System (Applied Biosystems, Inc. Foster City, CA, USA) for Nile tilapia. For seabream, 7500 Fast Real-Time PCR System (Applied Biosystems, Inc. Foster City, CA, USA) was used for tissue distribution in females while a StepOnePlus™ Real-Time PCR System (Applied Biosystems, Inc., Foster City, CA, USA), was used for seabream tissue distribution in males, and for SD and LD experiments, in which different primer sets were used for *sacart1b1* and *sacart1b2*. Elongation factor 1 alpha (*ef1α*) (57) and 18S (58) served as reference genes because they showed stability in previous feed-deprivation experiments in both species (52, 59). The geometric mean of the C_T_ values for both genes was used to normalize the data. Gene-specific primers used for qPCR were designed using Primer-BLAST for tilapia (60) or Primer3 for seabream. The *R*^2^ value and slope, calculated by linear regression for all the genes tested, are described in **Supplementary Table 6**. For tilapia, each reaction consisted of 5 microliter (µL) SYBR^®^ green dye (Thermo Fisher Scientific, Vilnius, Lithuania), 0.75 µL of 3 µM forward and reverse primers, 0.5 µL of Ultra-Pure Water (UPW) and 3 µL of cDNA template (all tissues diluted 1:10 in UPW). Analyses were performed in duplicates. For seabream PCR reactions were prepared in a final volume of 10 µl containing 2 µl of 50ng cDNA, 0.3 µl of each forward and reverse primer (10 µM) to total concentration of 3 µM, and 5 ul of the SYBR Green FastMix ROX Kit (Quanta bio). Reactions were conducted in 96-well plates, and samples were analyzed in triplicate. In all cases, for each gene, a negative control (2 µl of UPW instead of cDNA template) was included. Melt curve analysis was used to confirm single-product amplification. Amplification was performed under the following conditions: 95.0°C for 20s, 40 cycles at 95.0°C for 3s, and 60.0°C for 30s, followed by one cycle at 95.0°C for 15s 60.0°C for 1 min, 95.0°C for 15s for the generation of the melting curve. Fluorescence signals of the target, reference genes, and control group were analyzed using StepOne software Version 2.3 or by 7500 Software v2.3 Relative quantification was determined using the 2^−ΔΔ^*^C^*^T^ method (61). Values were compared with the midbrain average for each gene in tissue distribution analysis, or with the fed fish group in SD or LD experiments. Briefly, the first ΔCT is the difference between the CT of the target gene and the geometric mean of reference genes. The ΔΔCT is the difference between the mean of the ΔCT of either the midbrain or the fed control group, and ΔCT of each fish.

### 2.7 Statistical analysis

GraphPad Prism version 8 (GraphPad, San Diego, USA) was used to perform the statistical analyses. Grubb’s test was used to remove outliers. Statistical analysis on the cart expression in various tissues was done using One-Way ANOVA, and the p-value was adjusted for multiple comparisons using Tukey’s test. If the expression was detected in only two tissues, an unpaired t-test was performed. Non-expression tissues were not included in the analysis. Multiple Student’s t-test was employed to identify significant differences in the expression between treated and control groups in food deprivation experiments. The level of significance was set at P < 0.05.

## 3 Results

### 3.1 Bioinformatics analysis

Seven *cart* sequences were identified and cloned from Nile tilapia brain cDNA library on the basis of predicted nucleotide and peptide sequences in GenBank and (18). These sequences were submitted to GenBank as Nile tilapia (on, *Oreochromis niloticus*), *oncart1b1* (PP667319), *oncart1b2* (PP667318), *oncart2a* (PP667321), *oncart2b* (PP667322), *oncart3a* (PP667323), *oncart3b* (PP667324), and *oncart4* (PP667320). The tilapia sequences and a previously identified seabream (sa, *Sparus aurata*) *cart* sequence were then used as templates for tblastn search against whole-genome shotgun contigs (WGS) of the gilthead seabream (62). This search resulted in the identification of several WGS sequences containing partial *cart* homologous sequences (one corresponded to the previously cloned *sacart* gene), encompassing at least 2 predicted exons and sharing high homology with other piscine *cart* sequences. Putative *sacart* mRNAs and peptides were manually assembled according to their sequence homology to tilapia *cart* peptides. This analysis resulted in the identification of six putative *sacarts*, which were then cloned, sequence validated and submitted to GenBank as *sacart1b1* (PP667326), *sacart1b2* (PP667325), *sacart2a* (PP667328), *sacart3a* (PP667329), *sacart3b* (PP667330), and *sacart4* (PP667327). Homology analysis of tilapia and seabream *cart* peptides was performed using Geneious Prime algorithm (https://www.geneious.com/features/prime) and showed 92% identity shared between oncart1b2 and sacart1b2, and that both peptides were homologous to mammalian Cart peptides (57-58% identity; 73% similarity) and chicken cart1 (55% identity; 67% similarity; **Supplementary Table 7**). Conversely, oncart3b and sacart3b only showed 59% identity, and obtained the lowest homology level to other Cart peptides both within and between species (under 30% homology for oncart3b and under 39% homology for sacart3b). The *Cypriniform* zebrafish (dr; *Danio rerio*) cart1 showed highest homology to cart2 peptides of the *Perciforms* tilapia and seabream (61-62% identity; 74-80% similarity). Furthermore, drcart2 and drcart3 showed the highest homology to tilapia and seabream cart1b while drcart4 exhibited the highest homology to the tilapia and seabream cart3a (**Supplementary Table 3**).

**Figure 2:**
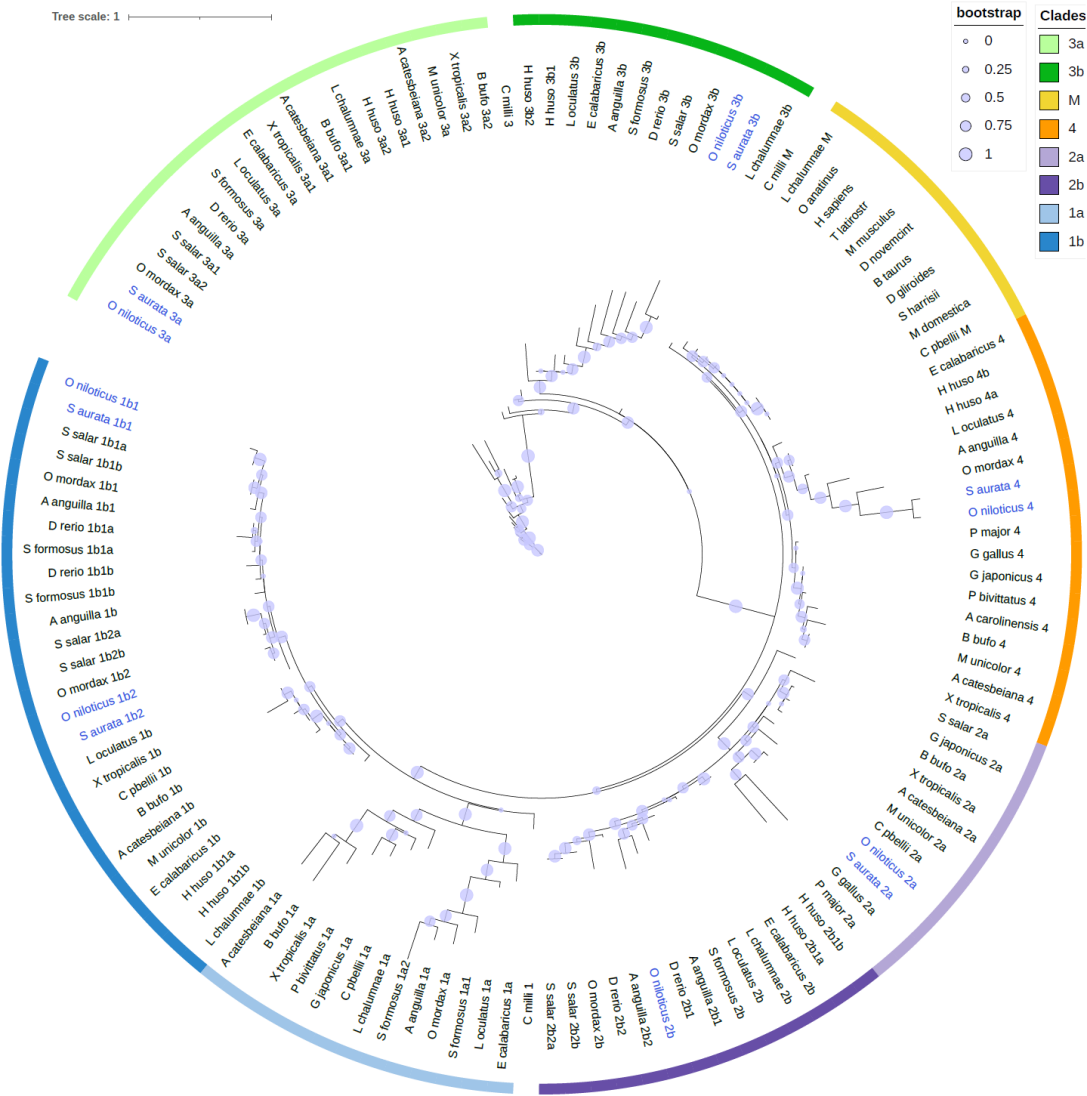
Phylogenetic tree of tilapia and seabream carts that show clustering into four clades. The accession numbers of the sequences used in this analysis are included in **Supplementary Tables 1**, **2**. Complete parameters of the PhyML run are detailed in **Supplementary Table 4**, and the alignment in **Supplementary File 3**.

**Figure 3:**
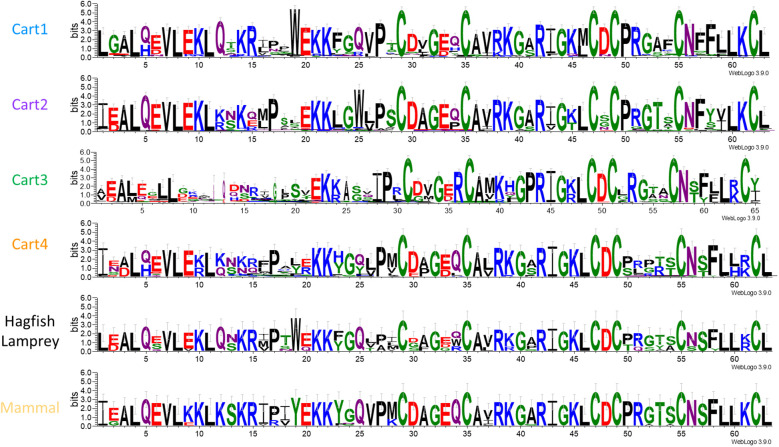
Sequence logos for each of the cart peptide groups showing sequence conservation among phylogenetic groups. If needed, multiple alignments of each group were cleaned of up to two extension-containing sequences, and a logo was generated using Weblogo 3.9.0. Sequences included in this analysis are detailed in **Supplementary File 4**.

The nomenclature of the cart family in fish is muddled, with various papers dividing the family into three (12), four (37), or six clades (18), with sequences assigned different names by different groups. A thorough bioinformatic search was performed in the genomes of various species spanning Eukarya in order to flesh out the family and create a phylogenetic tree and cohesive nomenclature. A balanced species distribution tree containing mammalian, avian, reptilian, amphibian, and piscine cart peptide sequences, with *Callorhinchus milii*, the ghost shark, as the most primordial species was constructed using the Maximum Likelihood method (**Figure 2**). This tree showed a four-clade structure. We then opted to track the evolutionary origins of cart and include more piscine species by searching putative cart sequences in various genomes. The earliest putative cart sequences (proto-cart) were found in tunicates and lancelets. In *Ciona intestinalis*, the single gene has four exons, but in all other species, the gene has three exons, with the second exon containing the di-basic cleavage signal, and the third exon the six cysteines (the exon starts with the first of the six) (63). The proto-cart do not have the cart domain using default parameters, but the domains can be found by relaxing the parameters. The next evolutionary group includes the *Agnathan* hagfish and lamprey that have recognizable cart domains (encoded by one gene and two genes, respectively), but are sufficiently distant to cause a slight distortion of the tree. The full tree (**Supplementary Figure 1**), including cart peptides from hagfish and lamprey, and many fish species, maintains the structure of the balanced analysis and divides clearly into four clades, each with two subclades. Based on the three cart peptides identified in the ghost shark and the six cart peptides of *Latimeria chalumnae*, the resulting tree showed that the vertebrate Carts identified to date fall into several distinct lineage groups (**Figure 2**). The first and second lineages included the reptilian, avian, amphibian and piscine cart1 and cart2 peptides, respectively. The third lineage contained amphibian and fish cart3 peptides and the fourth lineage contained mammalian and mammalian-like (M) cart peptides, as well as piscine, amphibian, reptilian and avian cart4 peptides. The tilapia and seabream carts cloned in the present study are distributed in all clusters (**Figure 2**). According to their phylogenetic clustering, both tilapia and seabream possess two cart1b paralogs (designated oncart1b1, oncart1b2). However, while tilapia possessed two cart2 paralogs (oncart2a, oncart2b), only one cart2 was identified in seabream (sacart2a). Nevertheless, two cart3 paralogs were identified in both tilapia and seabream (cart3a and cart3b). Both species possessed a cart4 gene which clustered with other cart4 peptides and exhibited highest phylogenetic proximity to the mammalian and mammalian-like cart peptide. Sequence logo analysis of the core cart peptides further supported our phylogenetic clustering and updated nomenclature (**Figure 3**). Nevertheless, while the six cysteine (Cys) residues (numbered I-VI) that form three stabilizing disulfide bonds in the human CART (2), were also conserved in all cart peptides analyzed, clear characteristic peptide signatures were identified for each cart type (**Figure 3**). These signatures remained clear also when performing sequence logo analyses for the subtype a and subtype b clusters of cart1, cart 2 and cart 3 peptides (**Supplementary Figure 2**) further supporting the nomenclature proposed herein.

### 3.2 Predicted structures of cart core peptide

**Figure 4:**
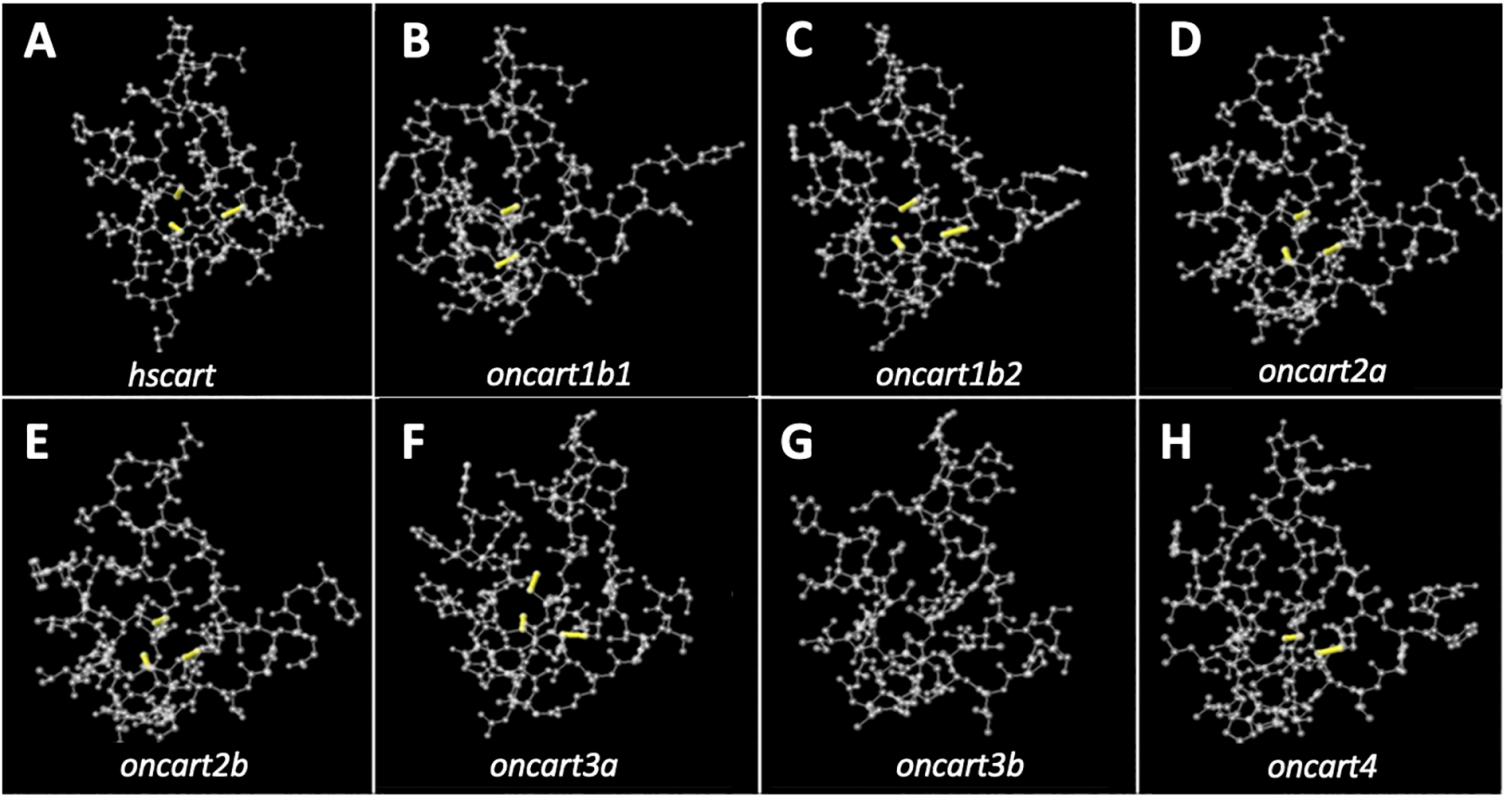
Predicted ball and stick 3D models of the bioactive form of Nile tilapia cart peptides showing conservation of disulfide bond configuration: human CART **(A)** oncart1b1 **(B)**, oncart1b2 **(C)**, oncart2a **(D)**, oncart2b **(E)**, oncart3a **(F)**, oncart3b **(G)**, and oncart4 **(H)**. Human cart peptide PDB entry: 1hy9; accession: Q16568) modeling shows the formation of 3 disulfide bridges. The yellow lines signify the presence of disulfide bonds. All predicted structures obtained a high confidence score (C-score) of >1.0 in a range of -5,2 indicating a high level of confidence in the accuracy and reliability of structures (**Supplementary Table 5**; **Supplementary Figure 3**).

**Figure 5:**
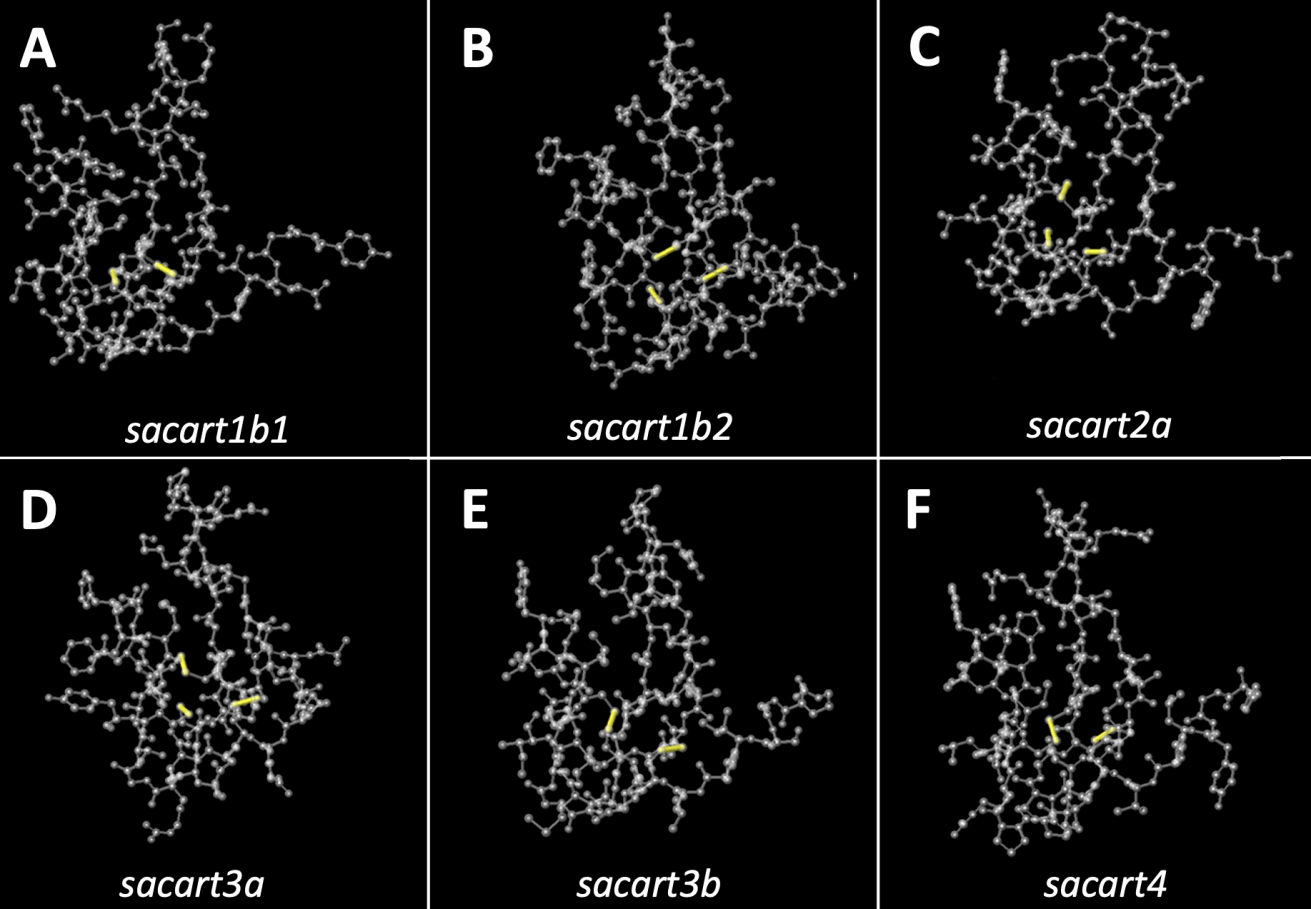
Predicted ball and stick 3D models of the bioactive form of gilthead seabream cart peptides showing conservation of disulfide bond configuration: sacart1b1 **(A)**, sacart1b2 **(B)**, sacart2a **(C)**, sacart3a **(D)**, sacart3b **(E)** and sacart4 **(F)**. The yellow lines signify the presence of disulfide bonds. All predicted structures obtained a high confidence score (C-score) of >1.0 in a range of -5,2 indicating a high level of confidence in the accuracy and reliability of structures (**Supplementary Table 6**; **Supplementary Figure 4**).

Translated cart prepropeptides (cartpt) ranged from 102–147 aa for tilapia and 104–127 aa for seabream. To gain a better understanding whether the high sequence conservation affects the resulting tertiary structure of cart peptides and related stabilizing disulfide bonds, we ran 3D models of the mature bioactive cart sequences. Tertiary protein structures of the tilapia and seabream cart core sequences were predicted using i-TASSER (49) and compared to the structure of human CART. Human CART has three disulfide bonds with a characteristic configuration consisting of Cys I-III (disulfide bond, DS1), Cys II-V (DS2) and Cys IV-VI (DS3), with DS1 not required for maintaining anorexic function (64). This analysis demonstrated that some carts retained all three disulfide bonds, while other piscine carts only retained one or two functional DSs. In tilapia, oncart1b2, oncart2a, oncart2b, and oncart3a were predicted to have all three characteristic disulfide bonds. oncart1b1 and oncart4 were predicted to possess two DSs, and no DS were present in the predicted structure of oncart3b (**Figure 4**). In seabream, sacart1b2, sacart2a, and sacart3a models present all three DS (**Figure 5**). sacart1b1 has two DS (DS1 lost) while sacart4 and sacart3b both have two DS, one of which is the non-functional DS1.

### 3.3 Central and peripheral cart gene expression

**Figure 6:**
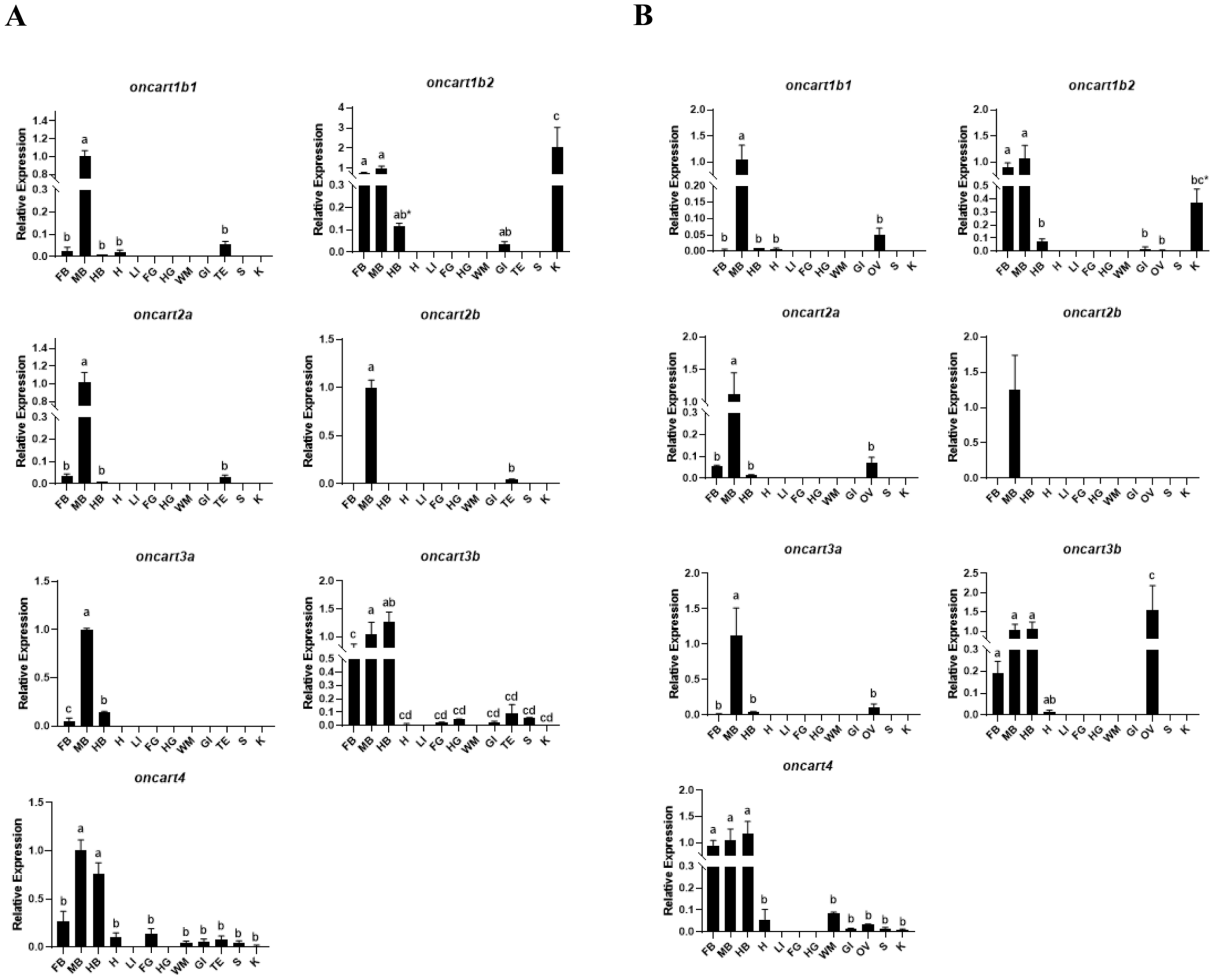
Distribution of tilapia CARTs expression in different tissues. **(A)** Adult male (n = 3) and **(B)** female (n = 3) Nile tilapia tissues were collected: FB, forebrain; MB, midbrain; HB, hindbrain; H, heart; LI, liver; FG, foregut; HG, hindgut; WM, white muscle; GI, gills; TE, testis; OV, ovary; S, stomach; K, kidney. Significant difference between tissues was determined using One-Way ANOVA and Tukey’s test for multiple comparisons. Tissues that do not share lowercase letters are significantly different from one another (p < 0.05). ab* p value = 0.060 and bc* p-value 0.082.

**Figure 7:**
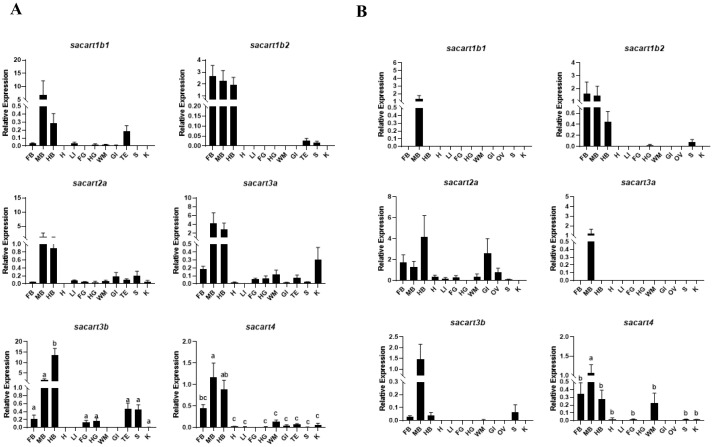
Distribution of seabream CARTs expression in different tissues. **(A)** Adult male (n = 4) and **(B)** female (n = 4) gilthead seabream tissues were collected: FB, forebrain; MB, midbrain; HB, hindbrain; H, heart; LI, liver; FG, foregut; HG, hindgut; WM, white muscle; GI, gills; TE, testis; OV, ovary; S, stomach; K, kidney. Significant difference between tissues was determined using One-Way ANOVA and Tukey’s test for multiple comparisons. Tissues that do not share lowercase letters are significantly different from one another (p < 0.05).

Quantitative PCR (qPCR) analyses of *cart* expression in different tissues were performed in order to gain insight into possible function(s) of the pleiotropic and multigenic cart system in each species. The brain was identified as the primary tissue expressing all cart genes in both tilapia and seabream, with the midbrain (MB) compartment, including the hypothalamus, as the primary site of expression (**Figures 6**, **7**). *cart* expression in the forebrain (FB) and hindbrain (HB) compartments varied in tilapia (**Figure 6**) as well as in the seabream (**Figure 7**). For instance, *oncart2b* was not expressed in either FB or HB, while *oncart1b1*, *oncart2a*, and *oncart3a* were expressed at significantly lower mRNA levels compared to the MB. Similarly, *oncart4* showed significantly lower expression in the FB in males (**Figure 6A**), whereas oncart1b2 showed significantly lower expression in the HB in females (**Figure 6B**). *oncart3b* also had significantly lower expression in the FB in males but not in females. Generally, the same expression pattern in the brain was observed in tilapia males and females. In seabream, *sacart1b2* and *sacart4* were highly expressed in FB, MB, and HB for both males and females, while for *sacart2a*, only males had low expression in the FB. For males, the rest of the *carts* showed higher expression in the HB compared to the FB, but only significant for *sacart3b* (**Figure 7A**). In seabream females, *sacart1b1* and *sacart3a* expression patterns were distinct from the males by not being expressed in FB and HB, and *sacart3b* had a very low expression in FB and HB.

In other tissues, qPCR analysis showed that *cart* genes were expressed in the tilapia gonads, with *oncart2b* selectively expressed in the testis, and *oncart1b2* and *oncart3a* selectively expressed in the ovary. Other *cart* genes were found to be expressed in both gonads (**Figure 6**). Besides, in females, *oncart3b* expression in the ovary was significantly higher than in other tissues. High transcript levels of *oncart1b2* were also detected in the kidney, which was significantly higher than in other tissues in males. Low *cart1b2* expression was also detected in the tilapia gills. *oncart4* was widely expressed in other peripheral tissues, including the gills, heart, kidney, white muscle, foregut (in males), and stomach*. oncart3b* expression was also detected specifically in peripheral tissues of male tilapia such as in the hindgut, foregut, gills, and stomach (**Figure 6A**). In seabream, all *cart* genes were expressed in the testis but only*sacart2a* was expressed in the ovary (**Figure 7B**). In males, all genes except *cart1b2* were widely expressed in peripheral tissues while in females only *sacart2a* and *sacart4* were widely expressed in the peripheral tissues. The expression of *sacart1b1* and *sacart3a* mRNAs in seabream females was restricted to the MB, while *sacart3b* was also expressed in FB, HB, white muscle and stomach.

### 3.4 Differential effects of short- and long-term food deprivation on cart expression

**Figure 8:**
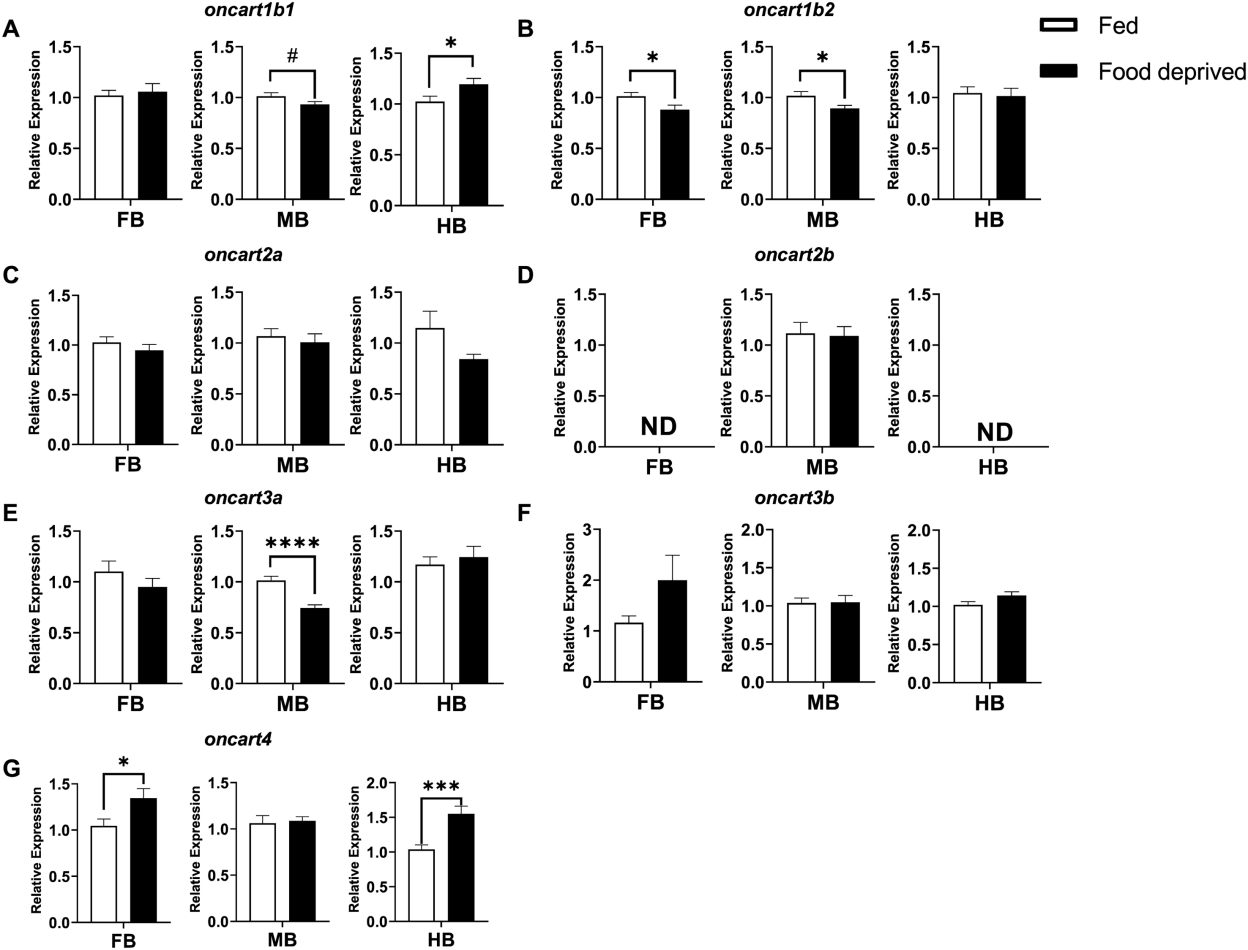
Relative expression of *cart* orthologues in major compartments of Nile tilapia brain following short term food deprivation (SD). Adult tilapia was food deprived for 24h. Expression of *oncart1b1* **(A)**, *oncart1b2* **(B)**, *oncart2a* **(C)**, *oncart2b* **(D)**, *oncart3a* **(E)**, *oncart3b* **(F)**, *oncart4* **(G)** was analyzed in the forebrain, (FB) midbrain region (MB), and hindbrain (HB) of the brain using qPCR (control n = 24, starved n = 24). Significant difference (p < 0.05) is indicated by (*), p <0.01 (**), p <0.001 (***), p <0.0001 (****) and p = 0.087 (#). Multiple t-test with significant value set at p < 0.05.

**Figure 9:**
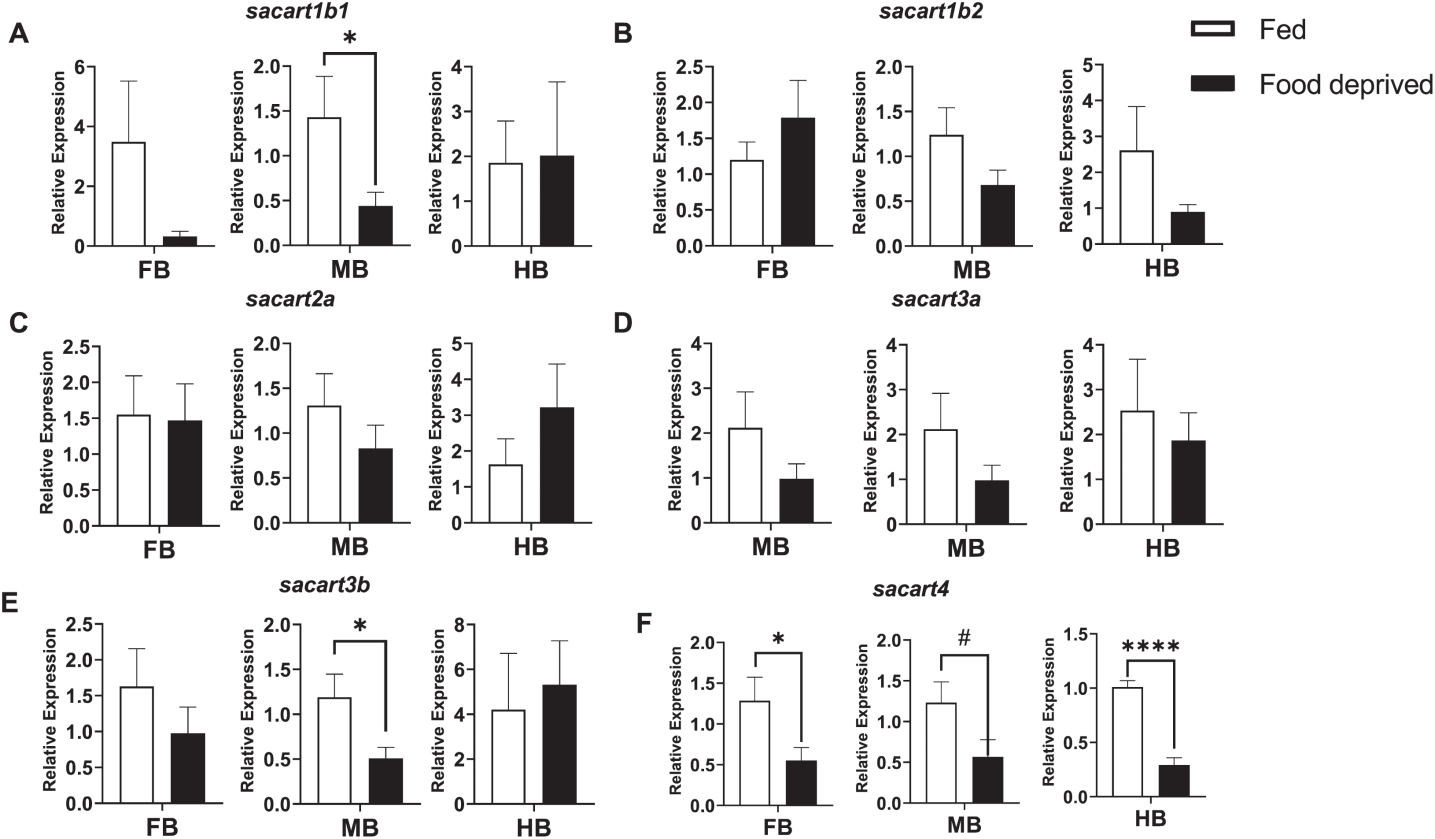
Relative expression of *cart* orthologues in major compartments of sea bream brain following SD. Adult gilthead seabream was food deprived for 48h. Expression of *sacart1b1* **(A)**, *sacart1b2* **(B)**, *sacart2a* **(C)**, *sacart3a* **(D)**, *sacart3b* **(E)**, *sacart4* **(F)** was analyzed in the FB, MB, and HB of the brain using qPCR (control n = 8, starved n = 10). Significant difference p < 0.05 is indicated by (*), p <0.0001 (****), and p = 0.059 (#).

Aiming to identify the anorexigenic nature of seabream and Nile tilapia *cart* genes, fish were deprived of food for short time periods (SD). Nile tilapia subjected to SD showed a significant decrease in MB expression of *oncart1b2* and *oncart3a*, as well as a decreasing trend in the expression of *oncart1b1* (p = 0.08) (**Figure 8**). *oncart1b2* decreased in expression in the FB, while *oncart1b1* increased in expression in the HB. SD also led to increased expression of *oncart4* in both FB and HB regions. Seabream subjected to SD exhibited a significant decrease in MB expression of *sacart1b1, sacart4* (p = 0.059) and *sacart3b* (**Figure 9**). Significantly decreased *sacart4* mRNA was also noted in the FB and HB compartments (**Figure 9**). These data show that the effect of negative energy balance on *cart* gene expression may vary according to the fish species.

**Figure 10:**
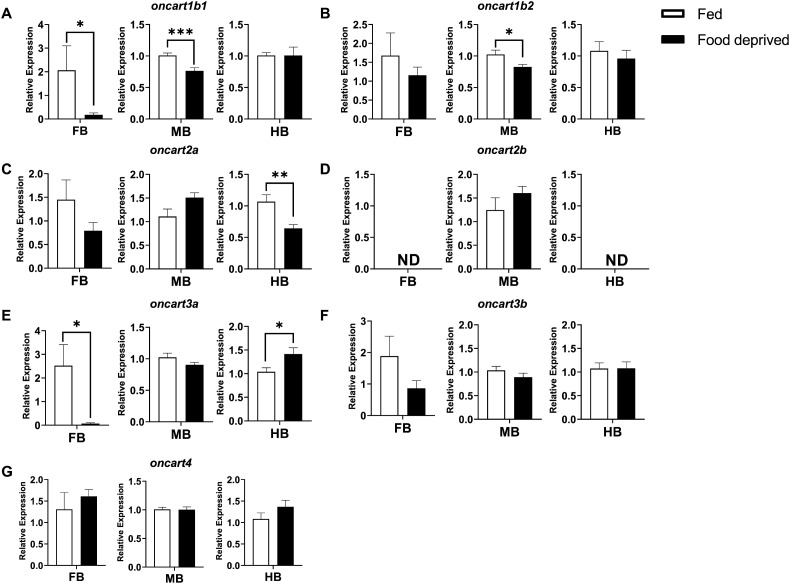
Relative expression of *cart* orthologues in major compartments of Nile tilapia brain following long term food deprivation (LD). Adult tilapia was food deprived for 21 days. Expression of *oncart1b1* **(A)**, *oncart1b2* **(B)**, *oncart2a* **(C)**, *oncart2b* **(D)**, *oncart3a* **(E)**, *oncart3b* **(F)**, *oncart4* **(G)** was analyzed in the FB, MB, and HB of the brain using qPCR (control n = 14, starved n = 14). Significant difference (p < 0.05) is indicated by (*), p <0.01 (**) and p<0.001 (***).

**Figure 11:**
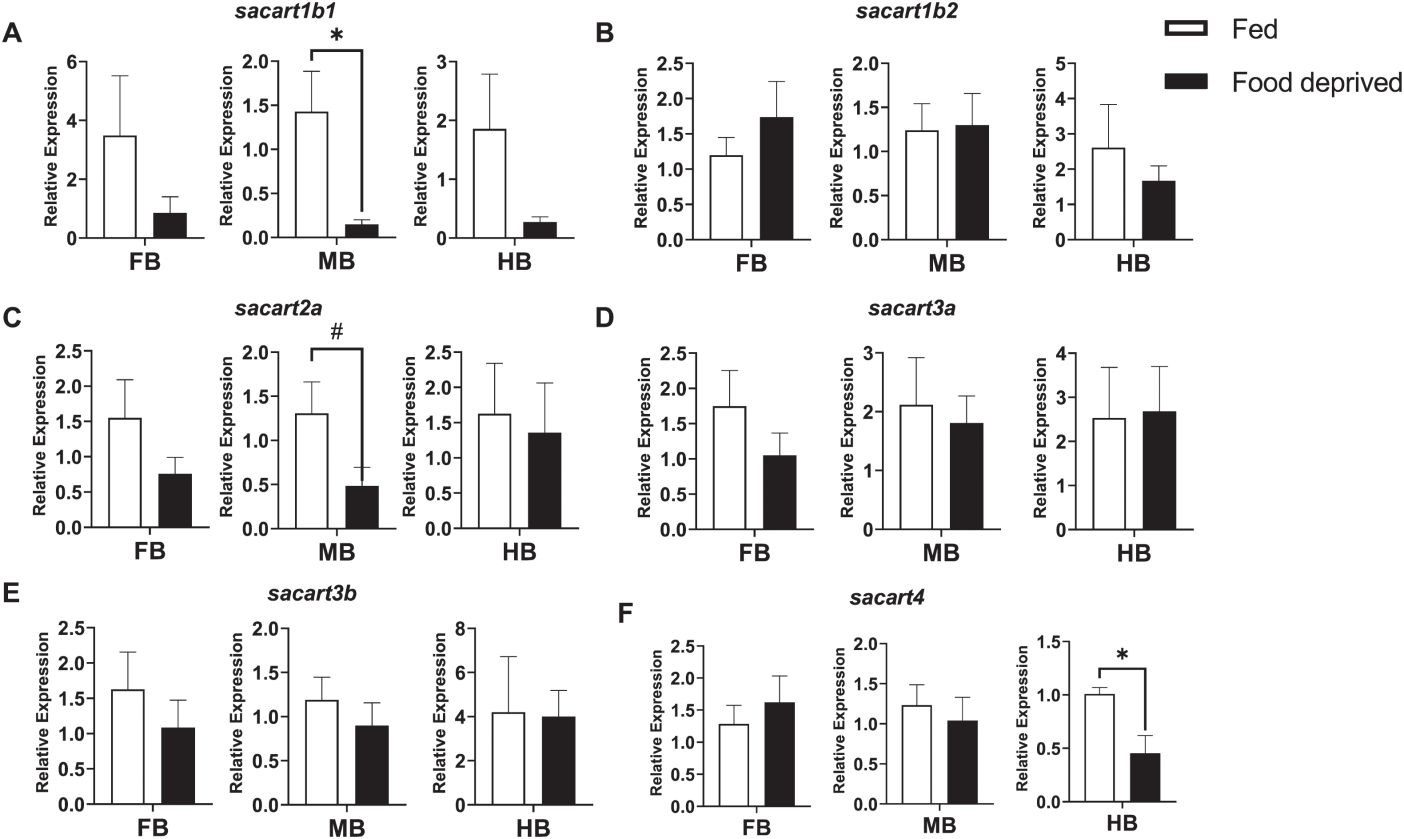
Relative expression of *cart* orthologues in major compartments of seabream brain following LD. Adult gilthead seabream was food deprived for 7 days. Expression of *sacart1b1* **(A)**, *sacart1b2* **(B)**, *sacart2a* **(C)**, *sacart3a* **(D)**, *sacart3b* **(E)**, *sacart4* **(F)** was analyzed in the FB, MB, and HB of the brain using qPCR (control n = 9, starved n = 9). Significant difference (p < 0.05) is indicated by (*) and p = 0.070 (#).

Long-term food deprivation (LD) for 21 days in Nile tilapia led to a decrease in expression of *oncart1b2* and *oncart1b1* in the MB (**Figure 10**). In the FB of tilapia, a decrease in expression of *oncart1b1* and *oncart3a* was observed. A significant decrease in expression of *oncart2a* was also observed in the HB. Moreover, while *oncart3a* expression significantly decreased in the FB, a significant increase in expression was observed in the HB. In seabream LD of 7 days resulted in decreased expression of *sacart1b1* and a trend (p = 0.070) of decreased expression of *sacart2a* in the MB (**Figure 11**). No significant change in expression in the FB was observed in the seabream, but a decreased expression of *sacart4* was identified in the HB.

## 4 Discussion

The role of Cart as an anorexigenic neuropeptide has been extensively studied in various vertebrate species (3, 65). The advent of high throughput new technologies allowed whole genome sequencing of fish species and therefore enabled the identification and characterization of duplicated genes (66–68). Herein, we describe the identification and cloning of seven *cart* genes in Nile tilapia and six *cart* genes in gilthead seabream-. Using phylogenetic analysis, we showed that the newly identified cart peptides cluster with various vertebrate cart sequences into four major lineages. Furthermore, we have bioinformatically identified putative cart peptides in ancient piscine, agnathans, and tunicates, and, based on their phylogeny and the sequence conservation of the core cart peptides, we propose a revised nomenclature herein. Tertiary structure modeling of the newly identified tilapia and seabream carts suggested that although the general tertiary structure was well conserved, some of the carts did not retain all of the stabilizing disulfide bonds. In addition, the variable expression patterns of *cart* genes in central compartments and peripheral tissues, both within and between species, further supported functional partitioning in this pleiotropic and multigenic system in fish.

Our current phylogenetic analysis further extended previous analyses (17, 38, 69) and suggests that the genomes of most taxonomic animalia classes contain multiple copies of *cart* genes, while tunicates and jawless vertebrates possess a single cart gene, as do mammals. Four to six cart genes can be found in the genomes of amphibians according to their taxonomic allocation to microcaelcilia or Anura, respectively. Avians possess two cart genes, and the number of *cart* genes in fish genomes may vary from five to ten genes probably due to family-specific whole genome duplications (*salmonidae*), or gene-specific duplication or reduction events (*S. senegalensis* and *C. semilaevis*, respectively). These findings suggest that evolutionary forces found various solutions to exert the pleiotropic functions of cart peptides, however further support for this claim requires clear identification of a cognate cart receptor for analysis of its functions and genomic phylogeny. Generally, cart3 and cart4 phylogenetic clades are the most diverse, and the sequences of cart a subtypes were more diverse than cart b subtypes. Whether this relates to their putative receptor binding or other function-related structure remains to be determined.

Protein and gene regulatory sequences may change throughout evolution (70). Nevertheless, we found that tilapia and seabream carts share high similarity with other vertebrate Carts, mainly at their C-terminus where the mature cart peptide resides. Mammalian Cart as well as non-mammalian cartM peptides show extremely high sequence conservation and contain highly conserved cleavage sites leading to the maturation of short (42 aa) and long (47 aa) Cart forms (71). However, in species with multiple cart genes, mostly cart1 and agnathan cart peptides contained the KR cleavage site required for the processing of the long cart form. In chicken, only cart4 (formerly cart1) possesses the KR cleavage site, which is responsive to 48-h food deprivation (18). In fish, it seems that at least one of the two carts that contain the KR cleavage site are usually responsive to food deprivation, suggesting that the post-translational processing of cartpt into the long cart form may be appetite-related (17, 37, 38, 72, 73). In tilapia and seabream, only cart1b sequences contain the KR cleavage site. These carts also share high sequence similarity with mammalian Cart peptides at their C-termini, suggesting a stronger association of these carts with appetite regulation. Structural variance of the peptide processing sites was also identified in the cleavage site of the short cart. For example, cart3 peptides, which are the least conserved cart peptides, lost the first two of their three proteolytic sites and terminate with tyrosine (Y; cart3a peptides) or isoleucine (I; cart3b peptides) instead of the Leucine that is shared in almost all other cart peptides. Another defining feature of the Cart peptide is the three pairs of cysteine residues that form disulfide bonds important for biological activity (64). All six cysteines are conserved in Carts of all vertebrates including the ones identified in the current work, except of the Atlantic salmon cart4 (69). Nevertheless, our 3D structure predictions suggested that not all cart peptides have a conserved configuration relative to the mammalian Carts. Based on these models, four tilapia carts and three seabream carts retained all three conserved disulfide bonds, while other carts have partially conserved disulfide bonds. It was previously suggested that only two out of three (DS2 and DS3) disulfide bonds are important for the anorexigenic function of Cart (64). As a clear Cart receptor has yet to be identified (74–76), predicting disulfide bond configuration in multigenic cart systems may alternatively support prediction of anorexigenic function for each peptide. Although no clear association between structural configuration and anorexigenic function was observed in the present study, it is noteworthy that *cart1b*, exhibiting consistent responses to both challenges, possessed the conserved disulfide bond configuration associated with biological activity. Collectively, these findings suggest that structural conservation may serve as a partial, though not definitive, indicator of anorexigenic functionality.

Mainly expressed in the brain, Cart is involved in regulating central functions, with its primary site of expression in the mammalian brain being the hypothalamic arcuate nucleus (nucleus lateralis tuberis in fish) (65, 77, 78). However, there is evidence showing that *cart* genes in mammals are further expressed in extra hypothalamic brain areas, some of which operate independently from the hypothalamus to regulate food intake (79, 80). In order to gain insight into the diverse roles of carts in the piscine central nervous system, we divided the brains into three major compartments (i.e. forebrain, midbrain and hindbrain) The high transcript levels of tilapia and seabream *carts* in the midbrain coincide with the involvement of *cart* in hypothalamic regulation of various homeostatic functions.

Generally, in both tilapia and seabream, the main site of *cart* genes expression is the MB. *cart1b2*, the most conserved *cart*, is highly expressed in the MB and in the FB of both species studied. Among its pleiotropic functions, cart is involved in olfaction (17, 81). It has also been suggested that the olfactory bulbs communicate with the hypothalamus to control feeding and reproduction (82, 83). This might suggest that *cart1b2* is involved in olfaction-related feeding signaling, yet further work is required to clarify this point. Moreover, high transcript levels of *oncart3b* were identified in the tilapia HB, while for seabream, both *cart3* genes were highly expressed in the same region only in males. Similarly, moderate-to-high expression levels of *cart4* were also identified in the HB in both species. High expression levels of *cart* have been previously demonstrated in the brain stem of Atlantic salmon and Senegalese sole (37, 73). In mammals, neural circuits in the hindbrain are involved in feeding and energy homeostasis. Satiety signals are primarily transmitted through afferent fibers of the vagus nerves, initially traveling from the upper gastrointestinal tract to the hindbrain (84). Multiple *cart*s expression in the HB may suggest a conserved role in fish. While our analyses of *cart* expression are provided at a brain-compartment resolution, it is possible that various or even seemingly conflicting cart functions would be exerted by differential *cart* expression in specific brain nuclei or neuronal clusters within a single compartment. The specific expression sites within the hypothalamus and possible co-expression of multiple *cart* genes in a specific neuronal population remain to be determined.

Our analysis demonstrated that most of the *cart* genes are also expressed in the gonads of tilapia and seabream testis, which is consistent with findings in other fish species (40, 85–88). Studies in mammals support the involvement of Cart in reproductive functions through its effects on *kisspeptin* and *gnrh* expression (89). In fish, it has been suggested that *cart* responds more to changes in energy status during spawning than to actual involvement in reproduction, such as in female walking catfish (*Clarias batrachus*) (90). Our findings that *cart* expression differs between males and females and between species suggest that Cart may serve as a paracrine mediator of gonadal metabolism or as an endocrine signal of the gonadal metabolic state to the brain and periphery. For example, while Nile tilapia are gonochoristic mouthbrooders, gilthead seabream are protandrous hermaphrodites and pelagic spawners. Differential modes of sexual development and reproductive strategies could require mode specific regulation of energy allocation (81, 91–93). Whether gonadal *cart* expression is locally related to reproductive functions or systemically involved in metabolic regulation remains to be determined.

**Figure 12:**
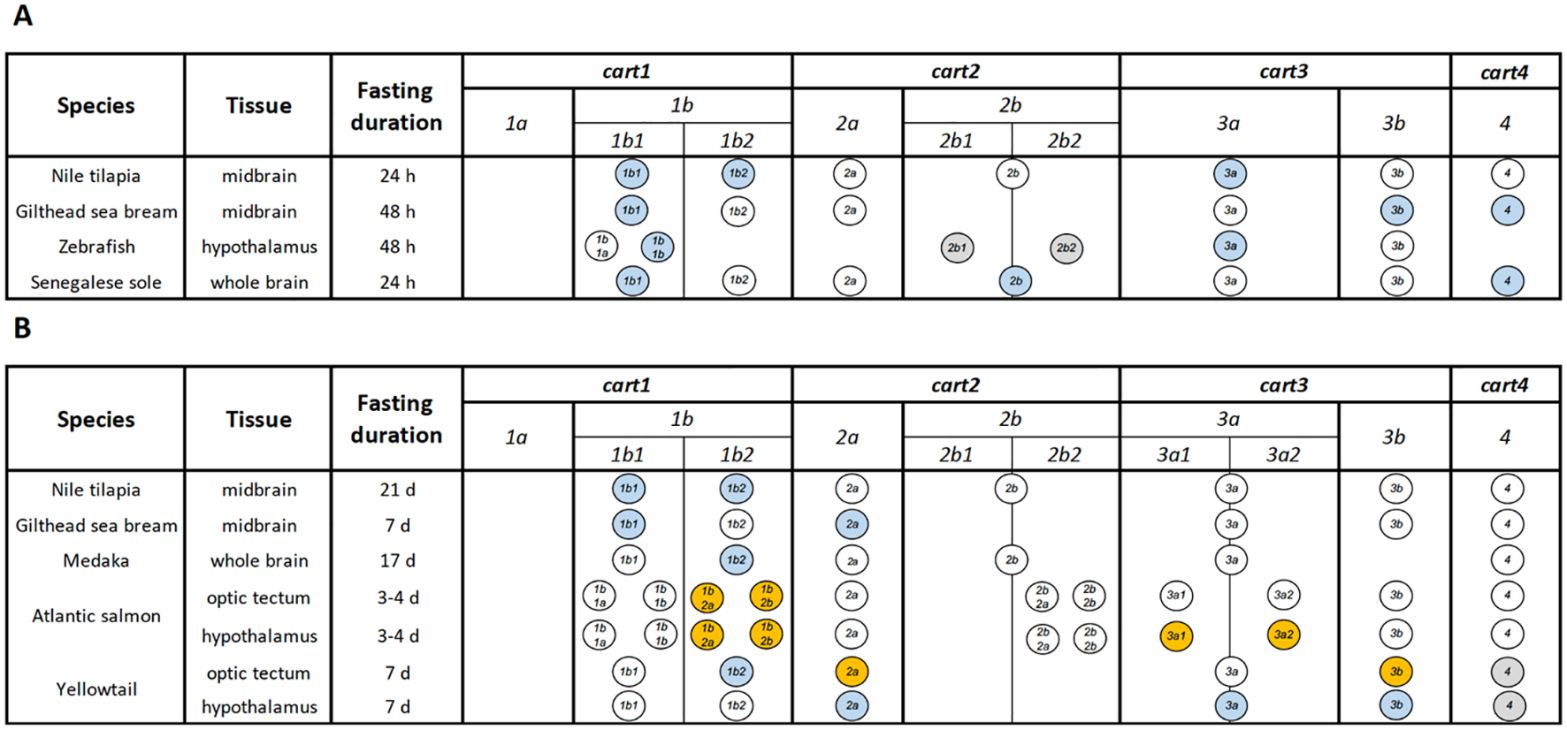
Responses of CART orthologs to food deprivation in fish species with fully sequenced genomes. **(A)** CART genes of Nile tilapia, gilthead seabream, zebrafish (17) and Senegalese sole (37) in response to SD for 24 or 48 h. **(B)** LD response of Nile tilapia, gilthead seabream, medaka (38), Atlantic salmon (69) and yellowtail (72) carts with varying durations. Blue-shaded circles indicate decrease in expression, orange-shaded circles indicate increase in expression, white-shaded circles indicate lack of significant response, and gray-shaded circles indicate genes that have been recently identified.

Fish have diverged from other vertebrate species in terms of long-term energy signaling (94, 95), as in the case of leptin, wherein its production is not affected by fat depots in fish (96). As we report, Nile tilapia and gilthead seabream *cart* gene responsiveness to negative energy balance was demonstrated to be specific. Following SD, *cart* expression in the midbrain significantly reduced for tilapia *cart1b2*, *cart3a*, and a trend to reduced *cart1b1* (p = 0.085), as well as for seabream *cart1b1*, *cart4* (p = 0.059), and *cart3b*. Although there are differences in experimental setup and tissue sampling in the existing literature, the current consensus is that SD results in downregulation of *cart* genes (18, 97–99). **Figure 12A** illustrates that in four fish species with fully sequenced genomes, SD caused a decrease in expression in any of the piscine carts (17, 37). Specifically, *cart1b2* in tilapia and *cart1b* genes in zebrafish are SD-responsive, while the *cart1b1* genes are responsive in seabream and Senegalese sole (*Solea senegalensis*) (37). *cart2* transcripts in both tilapia and seabream did not respond to SD, which is consistent with what was observed in zebrafish *cart1b1b* (17). For *cart3* genes, tilapia *cart3a*, seabream *cart3b*, and zebrafish *cart3a* exhibited decreased expression in response to SD. Expression of *cart3* genes of sole did not change in response to SD (37).

The effect of LD on *cart* genes expression is well-studied in mammals, birds, and fish, with a consensus that *cart* expression decreases after long-term fasting, with some species showing no response to negative energetic balance. The effect of negative energy balance on *cart* expression is more variable in species with multiple orthologues, such as chicken (*cart4*), medaka (*cart1b2*), and others (18, 38). As described for SD, the duration of feed restriction and the brain areas investigated in LD vary greatly depending on the experimental system and animal. In addition, most fish species studied to date do not have fully sequenced genomes. Thus, the complexity of the cart system in lower vertebrates might not be well-represented. In the current study, we identified a significantly reduced midbrain expression of specific *cart* genes in response to LD of Nile tilapia (*oncart1b1*, *oncart1b2*) and gilthead seabream (*sacart1b1*, *sacart2a*). This corroborates findings in other fish species. For example, LD led to decreased expression of *cart1b2* in the medaka brain, and suppressed *cart1b2* in the optic tectum of yellowtail (*Seriola quinqueradiata*; **Figure 12B**). In the yellowtail hypothalamus, LD suppressed *cart2a* gene expression as in seabream *cart2a* that clusters with it (100). Interestingly, yellowtail is the only species in which LD resulted in suppressed hypothalamic *cart3* (100). Evidently, *cart* genes that are involved in SD are not necessarily involved in LD, suggesting some form of functional partitioning that can be influenced by genetic background, feeding strategy, metabolic state of the fish or other factors. It should be noted, however, that differential expression alone does not establish direct functional involvement or confer specific biological role, and may reflect a generalized consequence of negative energy balance rather than a targeted regulatory response to the duration of feed deprivation.

By analyzing *cart* expression across brain regions, we showed that SD increased *oncart4* expression and decreased *oncart1b2* expression in the tilapia forebrain. In seabream, SD resulted in decreased *sacart4* in the same brain region. Similarly, SD in goldfish suppressed *cart* expression in the olfactory bulb (Volkoff and Peter, 2001b). LD caused a decreased expression of tilapia *cart1b1* and *cart3a* in the forebrain, which is in line with the results obtained in yellowtail wherein a decreased expression of *cart1b2 and cart2a* was observed in multiple regions of the forebrain following seven days of food deprivation (72). Contrastingly, the forebrain expression of yellowtail *cart3b* and Atlantic salmon *cart2b* increased in response to LD challenges (69, 72). Studies have shown that neural input from the telencephalon and olfactory bulbs may affect feeding in the forebrain (101). More studies are needed to elucidate the differential expression of cart genes in the forebrain in response to the animal’s energetic state and food availability. In the hindbrain, tilapia *cart4* increased while seabream *cart4* expression decreased following SD. LD supported reduced expression of tilapia *oncart2a* and seabream *sacart4* in the hindbrain. A similar decrease in the cerebellum was observed in yellowtail *cart1b1* after LD (100). It was suggested that the satiety signals produced during feeding, primarily transmitted through the afferent fibers of vagus nerves from the upper gastrointestinal tract to the hindbrain in mammals, may also be present in fish (69, 102). Further studies investigating different food deprivation durations and the response following re-feeding are needed to determine whether functional partitioning of *cart* genes also exists across other species, and whether each gene exhibits a temporal threshold to energy depletion. Such insights would provide a more comprehensive understanding of the evolutionary divergence and specialization of each *cart* orthologue in relation to metabolic regulation and brain region.

In conclusion, we identified 7 *cart* genes in Nile tilapia and 6 in gilthead seabream, two economically important fish species, thereby, improving the phylogenetic understanding of this multigenic system and demonstrating that it diverges into four Cart lineages. The CART peptides that retained the same structural configuration as human CART did not predict responsiveness to SD or LD. However, it is worth noting that *cart1b* genes, which are phylogenetically and structurally close to mammalian Carts, were responsive to both challenges (only *cart1b1* for seabream) and thus may exert a major influence in the anorexigenic function. Our findings not only confirm previous consensus that only a subset of carts respond to negative energy balance, but also suggest some form of functional partitioning that is dependent on energy status. Yet, the responsiveness of these cart genes was also species-specific, and may be related to their habitats (freshwater vs. marine), foraging behavior (omnivore vs. carnivore) as well as reproductive strategy (gonochoristic parenting vs protandrous hermaphrodite pelagic spawner) which require varying feeding physiology and energy allocation, and that CART function may have evolved to accommodate these factors. Taken together, our current findings suggest that although the piscine cart system is multigenic in nature, it maintained its important role in energy homeostasis. Further studies using other additional fish species are required to shed more light on how multiple carts regulate satiety in fish.

## Data Availability

The datasets generated for this study can be found in the GenBank according to the accession numbers detailed in the text.
