# Supplementary material for: Cocaine- and amphetamine-regulated transcripts in two percomorphs: evolutionary conservation and energy-status dependent responses
Source: Front Endocrinol (Lausanne). 2026 Jun 30;17:1870522. doi: 10.3389/fendo.2026.1870522 (PMC13364575; doi:10.3389/fendo.2026.1870522)
Supplement: Supplementary file 1 [file DataSheet1.docx]

CLUSTAL W (1.81) multiple sequence alignment

6Xtropicalis AAALEEMLDYN--QDKG--IRLQRRVGQLPWC--DVGGRCAMKRGPRIGKLCDCLRGTSC

5Acatesbeiana AVALGELLDYN--QDRG--LSLEKKASQLPRC--DVGERCAMKHGPRIGKLCDCLRGASC

5Bbufo AVALGEMLEYN--DPDG-GVALEKKAVQVPRC--DVGERCALKHGPRIGKLCDCLRGASC

5Lchalumnae VEAINDILEND--HDRP--ISVEKKASQIPRC--DVGERCAVKYGPRIGKLCDCLRGAAC

5Loculatus AEALEGLLDES--QDNR--VSVDKK-SLIPRC--DVGERCAVKHGPRIGKLCDCLRGAAC

6Ecalabaricus AEVLEGLLENN--QDNA--IAVDKKASQIPRC--DVGERCAMKHGPRIGKLCDCLRGAAC

7Hhuso VDALEGLLENS--SDTI---AVEKKANQIPRC--DVGERCALKYGPRIGKLCDCLRGAAC

8Hhuso VDALEGLLENS---SDT--IAVEKKASQIPRC--DVGERCALKYGPRIGKLCDCLRGAAC

3aSsenegalensis VEALQGVLGDS--DTLS--LSVEKKASVIPRC--DVGERCAMKHGPRIGRLCDCLRGTAC

4Csemilaevis QEALQSLLSDS---NAA-SLSVEKKAGVIPRC--DVGERCAMKHGPRIGRLCDCLRGTAC

6Aocellaris AEALEDMLDGD--EDNR--IQLEKKASVIPRC--DVGERCALKHGPRIGRLCDCMRGTAC

5Dclupeoides AEALEDFLEGE--QDNR--ISVEKKASVIPRC--DVGERCAMKHGPRIGRLCDCMRGTAC

4Drerio AEALDELLDGE--QDNR--ISLEKKASVIPRC--DVGERCAMKHGPRIGRLCDCMRGTAC

6Sformosus AEALGGLLEGE--QDHR--IFLEKKASVIPRC--DVGERCAMKHGPRIGRLCDCLRGTAC

5Aanguilla AEALEGLLDGT--QDNR--ITLEKKASVIPRC--DVGERCAMKHGPRIGRLCDCLRGTAC

6Cchanos VDALEGLLEGD--QDNR--ISLEKKASVIPRC--DVGERCAMKHGPRIGRLCDCLRGTAC

5Omordax ADALEGLLESG-QENSI-GLSVEKKASVIPRC--DVGERCAMKHGPRIGRLCDCMRGTAC

8Ssalar ADALERLLEGVQQDNRI-GLSVEKKASLIPRC--DVGERCAMKHGPRIGRLCDCLRGTAC

1Salpinus ADALERLLEGVQQDNRI-GLSVEKKASLIPRC--DVGERCAMKHGPRIGRLCDCLRGTAC

3Elucius ADALEGLLEDKQQDNMI-GLSVEKKASLIPRC--DVGERCAMKHGPRIGRLCDCLRGTAC

6Ssalar ADALEGLLEGGQQDNMI-GLSVEKKASLIPRC--DVGERCAMKHGPRIGRLCDCLRGTAC

2Salpinus ADALEGLLEGGQQDNMI-GLSVEKKASLIPRC--DVGERCAMKHGPRIGRLCDCLRGTAC

2Hcomes AEVLQGFLDEA-EGGGA-GVSREKKASFIPRC--DVGERCAMKHGPRIGRLCDCLRGTAC

S3bSquinqueradiata AEALQGFLDEA--DSRV-GLSVEKKASVIPRC--DVGERCAMKHGPRIGRLCDCLRGTAC

3Sdumerili AEALQGFLDEA--DSRV-GLSVEKKASVIPRC--DVGERCAMKHGPRIGRLCDCLRGTAC

2Xmaculatus AEALQGLLDEA--DSRV-GLSVEKKASVIPRC--DVGERCAMKHGPRIGRLCDCLRGTAC

7Aocellaris AEALQGLLDEA--DSRV-GLSVEKKASVIPRC--DVGERCAMKHGPRIGRLCDCLRGTAC

3aSaurata AEALQGFLDEA--DSSV-GLSVEKKASVIPRC--DVGERCAMKHGPRIGRLCDCLRGTAC

2Hburtoni AEALQGLLDEA--DSSA-GLSVEKKASVIPRC--DVGERCAMKHGPRIGRLCDCLRGTAC

3aOniloticus AEALQGLLDEA--DSSA-GLSVEKKASVIPRC--DVGERCAMKHGPRIGRLCDCLRGTAC

2Mzebra AEALQGLLDEA--DSSA-GLSVEKKASVIPRC--DVGERCAMKHGPRIGRLCDCLRGTAC

1Olatipes AEALQGLLDEA--DSSV-GLSVEKKASVIPRC--DVGERCAMKHGPRIGRLCDCLRGTAC

5Drerio VEAMTALLERY--QSHL--PSSEKR--AIPQC--ALGSRCAMRLGSRFGKLCECGRGSNC

1Elucius VDAMEALLVKM--ESRL--PSTEKR-GMIPPC--GVGQRCALRHGPHIGKLCDCGRVSSC

5Ssalar VGAMEALIVKM--QSHL--PTNEKR-GMIPPC--GMGDRCALRHGPRIGKLCDCGRVSSC

6Salpinus VDAMEALLVKM--QSHL--PSNEKR-GMIRPC--GMGDRCALRHGPRIGKLCDCGRVSSC

5Cchanos IEAMEALLGKY---PER--PHAEEKRG-IPTC--LTGSRCAVRLGPRIGKLCECGRGSNC

6Omordax MDVMETLLGKM---NHR-FPSTDKR-GSIPIC--GMRDRCAMRLGPRIGKLCDCGRGGNC

1Hburtoni LEALDVLLGRN--HNQV---SPEKR-GSIPLC--GLGNRCAMKYGPRIGKLCDCGRGANC

3bOniloticus LEALDVLLGRN--HNQV--SSPEKR-GSIPLC--GLGNRCAMKYGPRIGKLCDCGRGANC

1Mzebra LEALDVLLGRN--HNQV--SSPEKR-GSIPLC--GLGNRCAMKYGPRIGKLCDCGRGANC

1Aocellaris LEALEALLGRT--QNRV--PSTEKR-GSIPLC--GMGDRCAMKFGPRIGKLCDCGRAANC

6Xmaculatus VEALEVLLGRI--HSRV--SSTEKR-GSIPLC--GMGGRCAVKFGPRIGKLCDCGRGANC

1Csemilaevis IDALETLLGRM--HNRI---SYEKR-GSIPLC--GMGDRCAMKYGPRIGKLCDCGRGANC

4Hcomes VEALDVLLSRM--HNRI---STEKR-GNIPLC--GMGDRCAMKFGPRIGKLCDCGRGANC

3bSsenegalensis LEALEALLGRM--HNRI--SSTEKR-GSIPLC--GMGERCAMKYGPRIGKLCDCGRAANC

3aSquinqueradiata VEALEALLGRM--HNRI--SSTEKR-GSIPLC--GMGDRCAMKFGPRIGKLCDCGRGANC

4Sdumerili VEALEALLGRM--HNRI--SSTEKR-GSIPLC--GMGDRCAMKFGPRIGKLCDCGRGANC

3bSaurata VEALEALLGRM--HSRT--GSTEKR-GSIPLC--GMGDRCAMKFGPRIGKLCDCGRGANC

3Aanguilla LEAMEDLLGKF--QSRL--PSTEKR-GSIPLC--GVGDRCAVRLGPRIGKLCDCAGRRNC

2Cmilli FEAVEEILGKL--HNAI-SPSYEKKAGQIPKC--DIGDRCAIKQGPRIGKLCDCARGTTC

4Sformosus VEALEEILGKF--ENRV--P--EKR-GSIPTC--GRGERCAVKLGPRIGKLCDCGRGSHC

4Lchalumnae VEAMEELLGKF--QNRY--PTYEKKGGQIPLC--AIGERCAVKQGPRIGKLCDCSRGSSC

4Bbufo VEAMEELLEKF--QDRY--PVYQKR-AQIPLC--DIGERCAVKQGPRIGKLCDCSRGSSC

3Xtropicalis VEAMEELLGKF--QDRY--PTYQKK-AQIPLC--DIGERCAVKQGPRIGKLCDCSRGSSC

3Municolor VEAMEELLGKF--QNKY--PSYQKKAAQIPMC--DIGERCAVKQGPRIGKLCDCSRGAIC

4Acatesbeiana VEAMEELLGKS---------LYQKR-AQIPMC--DIGERCAVKQGPRIGKLCDCSRGSSC

4Ecalabaricus VETMEELLGKF--QSRL--PSYEKKGGTIPLC--DVGDRCAVKLGPRIGKLCNCARGSSC

3Loculatus VEAMEELLGKV--QSRF--PSYEKKAATIPMC--DVGDRCALRQGPRIGKLCDCARGSIC

4Hhuso VEAMEELLGKF--QSRF--PSYEKKAGTIPLC--DVGDRCAVKQGPRIGKLCDCARGSTC

6Hhuso VEAMEELLGKF--QSRF--PSYEKKAGTIPLC--DVGDRCAVKQGPRIGKLCDCARGSTC

2Omordax INDLQGVLERL--KNKR-FLPHAKKHSLLPMC--DAGEQCALRKGARIGKLCDCQQPRAC

6Olatipes ISDLHQVLERL--QSIQ-FPALRKKHGYLPVC--EPGEQCALRKGSRIGKLCDCSLPRTC

1Hcomes INDLHEVLDRL--QKNQ-LSALRKKHGHLPMC--DPGDQCALRRGSRIGKLCDCSLPRTC

4Ssenegalensis ISDLHEVLEKL--QYNQ-YPSLRKKHGYLPLC--DPGDLCALRKGSRIGKLCDCPLPRTC

5Xmaculatus INDLHQVLERL--QHNQ-FPALRKKHSYLPAC--DPGEQCALRKGSRIGKLCDCSLPRTC

7Hburtoni ISDLHDVLERL--QNHP-FPVLRKKHGYLPVC--DPGEQCALRKGSRIGKLCDCSLPRTC

7Mzebra ISDLHDVLERL--QNHP-FPVLRKKHGYLPVC--DPGEQCALRKGSRIGKLCDCSLPRTC

1cOniloticus INDLHEVLERL--QNHP-FPVLRKKHGYLPVC--DPGEQCALRKGSRIGKLCDCSLPRTC

1cSaurata INDLHEVLERL--QNNQ-FPALRKKHGYLPVC--DPGDQCALRRGSRIGKLCDCSPPRTC

8Aocellaris INDLHEVLERL--QNNQ-FPALRKKHGFLPVC--DPGDQCALRKGSRIGKLCDCSLPRTC

6Sdumerili INDLHEVLERL--QNNQ-FPALRKKHGYLPLC--DPGDLCALRKGSRIGKLCDCSLPRTC

nSquinqueradiata INDLHEVLERL--QNNQ-FPALRKKHGYLPLC--DPGDLCALRKGSRIGKLCDCSPPRTC

2Pmajor VEVLQEVLDKL--RTRE-PPALEKRLSWVPWC--EPREPCAVRRGARIGKLCSCPRGTSC

7Sformosus LDVLRNVLEGL--QKKR-LSVLQRRYRRLPGC--NVGDFCSVKRGARHGQLCDCPRGSKC

1Ecalabaricus LGALQEVLEKL--QHRR-LSTWDKKFSRVPRC--AIGDYCSVKKGARFGKLCDCPQEASC

4Loculatus LGALQHVLEKL--QNRR-LGTWEKKLSRLPQC--DIGDYCSVKKGARFGKLCDCPRGVKC

9Aanguilla LDVLHDVLEKL--RNRR-MAILERTHSRLPRC--SVGDFCSVKKGARFGQLCDCPRGSKC

5Sformosus LDVLHNVLEKL--QNRR-MGGWERKTSRLPVC--YIGDFCSVKKGSRFGQLCDCPRGSKC

6Dclupeoides LGVLQNVLEKL--QNRR-MTIWERKHSRLPNC--NVGDFCTVKKGPRFGQLCDCPRGSKC

7Omordax LGVLHNVLEKL--QNRR-MASWERRQSRLPSC--NVGDFCTAKKGPRFGQLCDCPRGSKC

8Salpinus LGVLHNVLEKL--QNRR-MAVWERRQSHLPSC--IVGDYCTVKKGPRYSQLCDCPRGSKC

2Ggallus VEALQEVLEKL--RSRE-LPPTAKKPGRVPSC--HLGEPCAVRVGARYGKRCSCPPGTAC

6Bbufo LLELQDVLYKL--QSKR-SPAWESKYIQVPKC--IMGDACAVKRGARIGKLCDCPPWSTC

6Acatesbeiana LLELQDVLEKL--QSKR-GILWESKLNQMPKC-LHHGDACAVKRGLRIGKLCDCPRRSVC

5Xtropicalis LTELQDVLEKL--QSKR-ILSWESKLNQVPKC--TLGDVCAVKRGARIGKLCDCPRRSNC

Eburgeri LEALQQVLEKL--QNKR-VPTWQKKFGQTPMC--SFGGRCALRRGPRIGKLCDCPQGTSC

Mglutinosa LEALQEVLEKL--QNKR-VPTWQKKYGQTPMC--SFGGRCALRRGPRIGKLCDCPQGTSC

4Dclupeoides LGALQEVLEKL--QTKR-VPPWEKKLGQVPTC--KFGEPCAVRKGARLGKMCECPPLTLC

gekko3 LEELQNVLEKL--QHKT-VSTWEKKFNLVPKC--SFGDLCAVKKGARIGKLCDCPRGSAC

21Pbivittatus LEELQEVLEKL--QHKK-VSPWEKKFNQVPKC--SFGDPCAIRKGARIGKLCDCPRRAAC

2cpb LEELQDVLEKL--QSKR-ISTWEKKHNQVPKC--SIGQACAVKKGARIGRLCDCPRGATC

6Lchalumnae LNALQGVLEKL--QSKR-ILTWEKKFNQVPKC--SIGNFCAVKKGARIGRLCDCPRWTSC

2Pmarinus LEALQEVLEKL--QSKR-IPTWEKKFGQVALC--GAGDQCAVRKGARIGKLCDCPRSYAC

2Lreissneri LEALQEVLEKL--QSKR-IPTWEKKFGQVALC--GAGDQCAVRKGARIGKLCDCPRSYAC

Gaustralis1 LEALQEVLVKL--QSKR-IPTWEKKFGQVAVC--GAGEQCAVRKGARIGKLCDCPRGAAC

1Cmilli LGALQEVLEKL--QSKR-LPTWEKKFGQLPLC--DIGEQCAVRKGARIGKLCDCPRSTGC

4cbp LGALQEALEKL--QKKR-IPPWGKKLGQVPAC--DVGELCAVRKASRIGKLCNCPRGATC

2aSsenegalensis LGALHEVLEKL--QTNR-IHLWEKKYGQVPSC--DLGEHCAVRKGSRIGKMCDCPRGASC

2Olatipes LGALHDVLERL--QTTR-INPWEKKYGQVPSC--DLGEHCAIRKGSRIGKMCDCPRGAFC

6Huburtoni LGALHEVLERL--QTKR-INPWEKKYGQVPSC--DLGEYCAIRKGSRIGKMCDCPRGAFC

1bOniloticus LGALHEVLERL--QTKR-INPWEKKYGQVPSC--DLGEYCAIRKGSRIGKMCDCPRGAFC

5Msebra LGALHEVLERL--QTKR-INPWEKKYGQVPSC--DLGEYCAIRKGSRIGKMCDCPRGAFC

3Aocellaris LGALHEVLERL--QTKR-INPWEKKYGQVPSC--DLGEHCAVRKGSRIGKMCDCPRGAFC

1bSaurata LGALHEVLEKL--QTKR-INPWEKKYGQVPSC--DLGEHCAVRKGSRIGKMCDCPRGAFC

2aSquinqueradiata LGALHEVLEKL--QTKR-INPWEKKYGQVPSC--DLGEHCAVRKGSRIGKMCDCPRGAFC

1Sdumerili LGALHEVLEKL--QTKR-INPWEKKYGQVPSC--DLGEHCAVRKGSRIGKMCDCPRGAFC

7Xmaculatus LGALHEVLEKL--QTKR-INPWEKKYGQVPSC--DLGEHCAIRKGSRIGKMCDCPRGAFC

4Omordax LGALHDVLEKL--QTKR-ISPWEKKFGQVPTC--DMGEHCAVRKGARIGKMCDCPRGAFC

2Elucius LGALHEVLEKL--QTKR-INPWEKKFGQLPTC--DLGEHCAVRKGARIGKMCDCPRGAFC

7Ssalar LGALHEVLKKL--QTKR-INPWEKKFGQVPTC--DVGDHCAVRKGARIGKMCDCPRGAFC

9Ssalar LGALHEVLKKL--QTKR-INPWEKKFGQVPTC--DVGDHCAVRKGARIGKMCDCPRGAFC

4Salpinus LGALHEVLKKL--QTKR-INPWEKKFGQVPTC--DVGEHCAVRKGARIGKMCDCPRGAFC

2Municolor LGALQEVLEKL--QSKR-VPVWEKKFGQVPTC--DIGEQCAVRKASRIGKLCNCPRGAVC

4Xtropicalis LGALQEVLEKL--QNKR-IPSWEKKFGQVPVC--DVGEQCAVRKASRIGKLCNCPRGAVC

3Bbufo LGALQEVLEKL--QSKR-VPLWEKKFGQVPVC--DMGEQCAVRKASRIGKLCNCPRGSVC

3Acatesbeiana LGALQEVLEKL--QSKR-VPAWEKKFGQVPVC--DVGEQCAVRKASRIGKLCNCPRGAVC

3Drerio LGALHDVLEKL--QSKR-ISLWEKKFGRVPTC--DVGEQCAIRKGSRIGKMCDCPRGAFC

4Cchanos LGALHDVLEKL--QSKR-ISLWEKKFGRVPLC--DVGEQCAVRKGSRIGKMCDCPRGAFC

3Lchalumnae VRALQEVLEKL--QSKR-VPTWEKKFGQVPMC--DVGEQCAVRKGARIGKLCDCPRGSIC

3Dclupeoides LGALHEVLEKL--QTKR-IPPWEKKFGRVPTC--DVGEQCAIRKGARIGKMCDCPRGAIC

3Hhuso LGALQEVLEKL--QSKR-VPAWEKKFGQVPTC--DVREQCAVRKGARIGKLCDCPRGAIC

5Hhuso LGALQEVLEKL--QSKR-VPAWEKKFGQVPTC--DVREQCAVRKGARIGKLCDCPRGAIC

3Ecalabaricus LGALQEVLEKL--QSKR-VPAWEKKFGQVPTC--DVGEQCAVRKGARIGKLCDCPRGAIC

3Sformosus LGALQEVLEKL--QTKR-IPPWEKKFGQVPTC--DVGKQCAVRKGARIGKMCDCPRGTFC

4Aanguilla LGALQDVLEKL--QKKR-IPSWEKKFGQVPTC--DVGEQCAVRKGARIGKMCDCPRRAFC

3Cchanos LGALQEVLEKL--QTKR-IPPWEKKFGQVPMC--DVGEQCAVRKGSRIGKMCDCPRGAFC

2Drerio LGALQEVLEKL--QTKR-IPPWEKKFGQVPMC--DLGEQCAIRKGSRIGKMCDCPRGALC

3Omordax LGALQDVLEKL--QTKR-LSMWEKKFGQVPTC--DVGEQCAVRKGARIGKMCDCPRGAFC

1Ssalar LGALHDVLKKL--QTKR-LPFWEKKFGQVPTC--DVGEQCAVRKGARIGKMCDCPRGAFC

10Ssalar LGALHDVLKKL--QTKR-LPFWEKKFGQVPTC--DVGEQCAVRKGARIGKMCDCPRGAFC

5Salpinus LGALHDVLKKL--QTKR-LPFWEKKFGQVPTC--DVGEQCAVRKGARIGKMCDCPRGAFC

5Elucius LGALHDVLEKL--QTKR-LPFWEKKFGQVPTC--DVGEQCAVRKGARIGKMCDCPRGAFC

2Aanguilla LGALQEVLEKL--QSKR-ISPWEKKFGQVPTC--DVGEQCAVRKGSRIGKMCDCPRGAFC

2Sformosus LGALQEVLEKL--QSKR-ISPWEKKFGQVPTC--DVGEQCAIRKGARIGKMCDCPRGAFC

2Loculatus LGALQEVLEKL--QSKR-IPTWEKKFGQVPTC--DVGEQCAVRKGARIGKMCDCPRGAFC

5Hcomes LGALQEVLEKL--QTKR-LPMWEKKFGQVPTC--DIGEQCAVRKGARIGKMCDCPRGAFC

3Olatipes LGALQEVLEKL--QAKR-LPSWEKKFGQVPMC--DVGEQCAVRKGARIGKMCDCPRGAFC

3Xmaculatus LGALQEVLEKL--QAKR-LPSWEKKFGQVPTC--DVGEQCAVRKGARIGKMCDCPRGAFC

2bSsenegalensis LGALQEVLEKL--QTKR-LPLWEKKFGQVPTC--DVGEQCAVRKGARIGKMCDCPRGAFC

1aSaurata LGALQEVLEKL--QAKR-LPMWEKKFGQVPTC--DVGEQCAVRKGARIGKMCDCPRGAFC

2bSquinqueradiata LGALQEVLEKL--QAKR-LPMWEKKFGQVPTC--DVGEQCAVRKGARIGKMCDCPRGAFC

5Sdumerili LGALQEVLEKL--QAKR-LPMWEKKFGQVPTC--DVGEQCAVRKGARIGKMCDCPRGAFC

4Hburtoni LGALQEVLEKL--QAKR-LPLWEKKFGQVPTC--DIGEQCAVRKGARIGKMCDCPRGAFC

1aOniloticus LGALQEVLEKL--QAKR-LPLWEKKFGQVPTC--DIGEQCAVRKGARIGKMCDCPRGAFC

3Mzebra LGALQEVLEKL--QAKR-LPLWEKKFGQVPTC--DIGEQCAVRKGARIGKMCDCPRGAFC

2Aocellaris LGALQEVLEKL--QAKR-LPLWEKKFGQVPTC--DVGEQCAVRKGARIGKMCDCPRGAFC

7Aanguilla ISALKGVLEKL--KNNR-FPLYGKKYGQLPMC--EAGERCALRKGARIGKLCDCPYRISC

Gaustralis2 LDALQSVLEKL--QSKR-MPNWEKKYGQLPIC--DAGEWCAVRKGARIGKLCDCTRGTSC

1Pmarinus LDALQSVLEKL--QNKR-MPSWEKKYGQLPIC--DAGEWCAVRKGARIGKLCDCTRGTSC

1Lreissneri LDALQSVLEKL--QNKR-MPSWEKKYGQLPIC--DAGEWCAVRKGSRIGKLCDCTRGTSC

4Municolor LEALQEVLEKL--KSKR-IPSYEKKYGQVPMC--EAGDQCAVRKGPRIGKLCDCPRRTSC

2Xtropicalis IDALQEVLEKL--KNKR-LPLFEKKYGQVPMC--DAGEQCAVRKGPRIGKLCDCPRRTSC

2Acatesbeiana IDALQEVLEKL--KNKR-LPLFEKKYGQVPMC--DAGEQCAVRKGPRIGKLCDCPRRTSC

2Bbufo INALQEVLEKL--KSKK-LPSFEKKYGQVPMC--DAGEQCAVRKGARIGRLCDCPRRTSC

6Loculatus IEALQEVLEKL--KNKG-MPFYGKKYGQLPMC--EAGEQCALRKGARIGKLCDCPRATSC

2Ecalabaricus IDALQEVLEKL--KNKR-IPYYEKKIGQLPMC--DAGDQCAVRKGARIGKLCDCPRGTSC

1Hhuso IEALQEVLEKL--KSKR-MPYYEKKYGQLPMC--DAGEQCALRKGARIGKLCDCPRGTSC

2Hhuso IEALQEVLEKL--KSKR-MPYYEKKYGQLPMC--DAGEQCALRKGARIGKLCDCPRGTSC

1Lchalumnae IEALQEVLEKL--KSKR-IPVYEKKYNQVPMC--DAGEQCALRKGSRIGKLCDCPRGTSC

Sharrisii IGALQEVLKKL--KSKR-IRIYEKKYGQVPKC--DAGEQCAIRKGARIGKLCDCPRGTSC

Mdomestica IGALQEVLKKL--KSKR-IRIYEKKYGQVPKC--DAGEQCAIRKGARIGKLCDCPRGTSC

Dgliroides IGALQEVLKKL--KSKR-IRIYEKKYGQVPKC--DAGEQCAIRKGARIGKLCDCPRGTSC

3Cmilli IEALQEVLEKL--KSKR-MPTYEKKFGMVPMC--DAGEQCAVRKGARIGKLCDCPRGTFC

Oanatinus IEALQEVLKKL--KSKR-IPVYEKKYSQVPMC--DAGEQCAVRKGARIGKLCDCPRGTAC

Tlatirostris IEALQEVLKKL--KSKR-IPIYEKKYGQVPMC--DAGEQCAVRKGARIGKLCDCPRATSC

Hsapiens IEALQEVLKKL--KSKR-VPIYEKKYGQVPMC--DAGEQCAVRKGARIGKLCDCPRGTSC

Btaurus IEALQEVLKKL--KSKR-IPIYEKKYGQVPMC--DAGEQCAVRKGARIGKLCDCPRGTSC

Mmusculus IEALQEVLKKL--KSKR-IPIYEKKYGQVPMC--DAGEQCAVRKGARIGKLCDCPRGTSC

Dnovemcintus IEALQEVLKKL--KSKR-IPIYEKKYGQVPMC--DAGEQCAVRKGARIGKLCDCPRGTSC

1Pmajor IEALQEVLEKL--KSKR-GPHYEKKFGQVPMC--DAGEQCAVRKGARIGKLCDCPRGTSC

1Ggallus IEALQEVLEKL--KSKR-VPHYEKKFGQVPMC--DAGEQCAVRKGARIGKLCDCPRGTSC

1cpb IEALQEVLEKL--KSKR-IPVYEKKFGQVPMC--DAGEQCAVRKGARIGKLCDCPRGTSC

1Pbivittatus IEALQEVLEKL--KSKR-LPHYEKKYGQVPMC--DAGEQCAVRKGARIGKLCDCPRGTSC

gekko1 IEALQEVLEKL--KSKR-LPHYEKKYGQVPMC--DAGEQCAVRKGARIGKLCDCPRGTSC

anole IEALQEVLEKL--KSKR-LPLYEKKYGQVPMC--DAGEQCALRKGARIGKLCDCPRGTSC

6Drerio IEALQEVLEKL--KNKQ-LPQTGKKLSLLPSC--DAGEQCAIRKGARVGKLCSCPQGTSC

2Dclupeoides IEALQEVLEKL--KNKQ-MPKSARNFGMLPSC--DAGEQCAIRKGARVGKLCGCPPGMAC

2Cchanos IEALQEVLEKL--KNKQ-MPNSGKKFGRLPSC--DAGEQCAVRKGARVGKLCGCPQGTAC

1aSsenegalensis IEALQEVLEKL--KSKQ-LPSTEKKLGWLPSC--DVGQQCALRKGSRIGKLCSCPGGNVC

3Csemilaevis IEALQDVLEKL--KSKQ-LPSTEKKQSWLPSC--DAGQLCALRKGSRIGKLCGCPTGTVC

1Drerio IEALQEVLEKL--RNKQ-IPAVEKKLGWVPSC--DAGEQCAVRKGSRFGKLCSCPGGTAC

1Cchanos IEALQEVLEKL--RNKD-MPTTEKKFGWVPPC--DAGEQCAVRKGARFGKLCSCSGGTTC

1Dclupeoides IEALQEVLERL--KNTE-KPPAEKKLGWVPSC--DAGEPCAVRKGARIGKLCACPRGTSC

5Olatipes IEALQEVLEKL--RNKQ-LPSSEKKLGWLPPC--NTSEQCAVRKGARVGKLCGCPRGMEC

3cpb IDALQEVLEKL--KTER-LPSIEKKLGSVASC--DAGEPCAVRKGARIGRLCSCPRGTAC

4Ssalar FEALQEVLEKL--QSKQ-MPAYEKKLGWVPMCDADAGQQCAVRKGARIGKLCECPRGTSC

7Salpinus FEALQEVLEKL--QSKQ-MPAYEKKLGWVPMCDADAGQQCAVRKGARIGKLCECPRGTSC

4Elucius FDALQEVLEKL--KNKQ-TPSYEKKLGWVPMC--DAGQQCAVRKGARIGKLCECPRGTSC

1Xmaculatus IEALQEVLEKL--KNKQ-LPSSEKKLGWLPPC--DAGEQCAVRKGARIGKLCGCPRGTLC

4Aocellaris IEALQEVLEKL--KNKQ-LPSSEKKLGWLPAC--DAGEQCAVRKASRIGKLCGCPRGTVC

5Hburtoni IEALQEVLEKL--KGKQ-LPSSEKKLGWLAAC--DAGEQCAIRKASRIGKLCGCPGGTAC

4Mzebra IEALQEVLEKL--KGKQ-LPSSEKKLGWLAAC--DAGEQCAIRKASRIGKLCGCPGGTAC

2bOniloticus IEALQEVLEKL--KGKQ-LPSSEKKLGWLAAC--DAGEQCAIRKASRIGKLCGCPGGTVC

6Aanguilla IEALQEVLEKL--KNQR-MPATEKKLGWVSSC--DAGEECALRKGARIGKLCSCPRGTSC

gekko2 MEALQEVLEKL--RSSRLLPPLEKKLGWVPSC--DAGESCAVRKGSRIGKLCNCPRRTSC

4Olatipes IDALQGVLEKL--RNKE-MP-LEKKLGWLPSC--DAGEPCAVRKGARIGTLCGCPRGTSC

3Hcomes IDALQEVLEKL--RNKE-MPS-EKKLGWLPSC--DAGEPCAVRKGSRIGTLCSCPRGTSC

5Csemilaevis IDALQEVLEKL--RSKE-MPS-EKKHGWLPSC--DAGEPCALRKGARIGTLCSCPRGTSC

3Hburtoni IDALQEVLEKL--RSKE-MP-LEKKHGWLPSC--DAGEPCAVRKGARIGTLCSCPRGTTC

2aOniloticus IDALQEVLEKL--RSKE-MP-LEKKHGWLPSC--DAGEPCAVRKGARIGTLCSCPRGTTC

6Mzebra IDALQEVLEKL--RSKE-MP-LEKKHGWLPSC--DAGEPCAVRKGARIGTLCSCPRGTTC

4Xmaculatus IDALQEVLEKL--RNKE-MP-LEKKLGWLPSC--DAGEPCAVRKGARIGTLCSCPRGTAC

2Saurata IDALQEVLEKL--RSKE-MP-LEKKLGWLPSC--DAGEPCAVRKGARIGTLCSCPRGTSC

1bSquinqueradiata IDALQEVLEKL--RSKE-MP-LEKKLGWLPSC--DAGEPCAVRKGARIGTLCSCPRGTSC

5Aocellaris IDALQEVLEKL--RSKE-MP-LEKKLGWLPSC--DAGEPCAVRKGARIGTLCSCPRGTSC

2Sdumerili IDALQEVLEKL--RSKE-MP-LEKKLGWLPSC--DAGEPCAVRKGARIGTLCSCPRGTSC

1bSsenegalensis IDALQEVLEKL--RSKE-MP-LEKKLGWLPSC--DAGEPCAVRKGARIGTLCSCPRGTAC

1Municolor IEALQDVLKKL--ESKR-MPSLEKKLGWLPSC--DAGEQCAVRKGARIGKLCSCPRGTAC

1Xtropicalis IDALQEVLEKL--KSKR-ILPLDKKLGWVPSC--DAGEQCAVRKGARIGKLCNCPRGTAC

1Acatesbeiana IEALQDVLEKL--KSKR-ILPLDKKLGWVPSC--DAGEQCAVRKGARIGKLCNCPRGTTC

1Bbufo IEALQEVLEKL--KSKK-ILPLDKKLGWVPSC--DAGEQCAVRKGARIGKLCNCPRGTSC

1Omordax IEALQEVLEKL--KNKQ-MPSSEKKLGWLPSC--DAGEQCAVRKGARVGTLCGCPRGTSC

2Ssalar IEALQEVLEKL--KNKQ-MPLSEKKLGWLPSC--DAGEQCAVRKGARVGTLCGCPRGTTC

3Ssalar IEALQEVLEKL--KNKQ-MPLSEKKLGWLPSC--DAGEQCAVRKGARVGTLCGCPRGTTC

6Elucius IEALQEVLEKL--KNKQ-IPLSEKKLSWLPSC--DAGEQCAVRKGARVGTLCGCPRGTTC

3Salpinus IEALQEVLEKL--KNKQ-MPLSEKKLSWLPSC--DAGEKCAVRKGARVGTLCGCPRGTTC

1Aanguilla IEALQEVLEKL--KNKQ-MPSAEKKLGWLPSC--DAGEQCAIRKGARIGQLCGCPRGTSC

1Sformosus IEALQEVLEKL--KNKQ-MPSAEKKLGWVPSC--DAGEQCAIRKGARIGKLCNCPRGTSC

9Hhuso IEALQEVLEKL--KSKR-LPSAEKKLGWVPSC--DAGEQCAVRKGSRIGKLCNCPRGTSC

10Hhuso IEALQEVLEKL--KSKR-LPSAEKKLGWVPSC--DAGEQCAVRKGSRIGKLCNCPRGTSC

2Lchalumnae IEALQEVLEKL--KNKR-VPSAEKKLGWVPSC--DAGEQCAVRKGARIGKLCNCPRGTSC

5Ecalabaricus IEALQEVLEKL--KSKR-MPSAEKKLGWVPSC--DAGEQCAVRKGARIGKLCNCPRGTSC

1Loculatus IEALQEVLEKL--KNKR-MPSAEKKLGWVPSC--DAGEQCAVRKGARIGKLCNCPRGTSC

: : * *: . . . . * * *

6Xtropicalis NSFLLRCY--------

5Acatesbeiana SSFMLRCY--------

5Bbufo NSFMLRCY--------

5Lchalumnae NTFLLRCY--------

5Loculatus NTFLLRCY--------

6Ecalabaricus NTFLLRCY--------

7Hhuso NTFLLRCY--------

8Hhuso NTFLLRCY--------

3aSsenegalensis NTFFLRCY--------

4Csemilaevis NTFFLRCY--------

6Aocellaris NTFFLRCY--------

5Dclupeoides NTFFLRCY--------

4Drerio NTFFLRCY--------

6Sformosus NSFFLRCY--------

5Aanguilla NSFFLRCY--------

6Cchanos NTFFLRCY--------

5Omordax NTFFLRCY--------

8Ssalar NTFFLRCY--------

1Salpinus NTFFLRCY--------

3Elucius NTFFLRCY--------

6Ssalar NTFFLRCY--------

2Salpinus NTFFLRCY--------

2Hcomes NTFFLRCY--------

S3bSquinqueradiata NTFFLRCY--------

3Sdumerili NTFFLRCY--------

2Xmaculatus NTFFLRCY--------

7Aocellaris NTFFLRCY--------

3aSaurata NTFFLRCY--------

2Hburtoni NTFFLRCY--------

3aOniloticus NTFFLRCY--------

2Mzebra NTFFLRCY--------

1Olatipes NTFFLRCY--------

5Drerio NSFLLKCI--------

1Elucius NSFLLKCL--------

5Ssalar NSFLLKCL--------

6Salpinus NSFLLKCL--------

5Cchanos NSFLLKCI--------

6Omordax NSYLLKCI--------

1Hburtoni NSYLLKCI--------

3bOniloticus NSYLLKCI--------

1Mzebra NSYLLKCI--------

1Aocellaris NSYLLKCI--------

6Xmaculatus NSYLLKCI--------

1Csemilaevis NSYLLKCI--------

4Hcomes NSYLLKCI--------

3bSsenegalensis NSYLLKCI--------

3aSquinqueradiata NSYLLKCI--------

4Sdumerili NSYLLKCI--------

3bSaurata NSYLLKCI--------

3Aanguilla NSFLLKCI--------

2Cmilli NSFLLKCI--------

4Sformosus NSFLLKCI--------

4Lchalumnae NSFLLKCI--------

4Bbufo NSFLLKCI--------

3Xtropicalis NSFLLKCI--------

3Municolor NTFLLKCI--------

4Acatesbeiana NTFLLKCI--------

4Ecalabaricus NSFLLKCI--------

3Loculatus NSFLLKCI--------

4Hhuso NSFLLKCI--------

6Hhuso NSFLLKCI--------

2Omordax SSFMLRCL--------

6Olatipes NSFLHRCF--------

1Hcomes NSFLHRCL--------

4Ssenegalensis NSFLHRCL--------

5Xmaculatus NSFLHRCL--------

7Hburtoni NSFLHRCL--------

7Mzebra NSFLHRCL--------

1cOniloticus NSFLHRCL--------

1cSaurata NSFLHRCL--------

8Aocellaris NSFLHRCL--------

6Sdumerili NSFLHRCL--------

nSquinqueradiata NSFLHRCL--------

2Pmajor NLFILKCS--------

7Sformosus NYFFLKCL--------

1Ecalabaricus NFFFLKCL--------

4Loculatus NFFFLKCL--------

9Aanguilla NYFFLKCLTPPALWAP

5Sformosus NFFFLKCL--------

6Dclupeoides NLFFLKCL--------

7Omordax NHFFLKCL--------

8Salpinus NLFFLKCL--------

2Ggallus NLYVLRCS--------

6Bbufo SLFFMRCL--------

6Acatesbeiana NQFFLRCL--------

5Xtropicalis NYYFLRCL--------

Eburgeri SSYLLKCL--------

Mglutinosa SSFLLKCL--------

4Dclupeoides HPIVLKCF--------

gekko3 NTFLLKCL--------

21Pbivittatus NAFLLKCL--------

2cpb NTFLLKCL--------

6Lchalumnae NSFLLKCL--------

2Pmarinus NSFLLRCL--------

2Lreissneri NSFLLRCL--------

Gaustralis1 NSFLLRCL--------

1Cmilli NFFLLKCL--------

4cbp NFFLLKCL--------

2aSsenegalensis HFLLLKCL--------

2Olatipes NFFLLKCL--------

6Huburtoni NFFLLKCL--------

1bOniloticus NFFLLKCL--------

5Msebra NFFLLKCL--------

3Aocellaris NFFLLKCL--------

1bSaurata NFFLLKCL--------

2aSquinqueradiata NFFLLKCL--------

1Sdumerili NFFLLKCL--------

7Xmaculatus NFFLLKCL--------

4Omordax NFFLLKCL--------

2Elucius NFFLLKCL--------

7Ssalar NFFLLKCL--------

9Ssalar NFFLLKCL--------

4Salpinus NFFLLKCL--------

2Municolor NFFLLKCL--------

4Xtropicalis NFFLLKCL--------

3Bbufo NFFLLKCL--------

3Acatesbeiana NFFLLKCL--------

3Drerio NYFLLKCL--------

4Cchanos NFFLLKCL--------

3Lchalumnae NFFLLKCL--------

3Dclupeoides NFFLLKCL--------

3Hhuso NFFLLKCL--------

5Hhuso NFFLLKCL--------

3Ecalabaricus NFFLLKCL--------

3Sformosus NFFLLKCL--------

4Aanguilla NFFLLKCL--------

3Cchanos NFFLLKCL--------

2Drerio NFFLLKCL--------

3Omordax NFFLLKCL--------

1Ssalar NSYLLKCL--------

10Ssalar NSYLLKCL--------

5Salpinus NSYLLKCL--------

5Elucius NFFLLKCL--------

2Aanguilla NFFLLKCL--------

2Sformosus NFFLLKCL--------

2Loculatus NFFLLKCL--------

5Hcomes NSFLLKCL--------

3Olatipes NFFLLKCL--------

3Xmaculatus NFFLLKCL--------

2bSsenegalensis NFFLLKCL--------

1aSaurata NFFLLKCL--------

2bSquinqueradiata NFFLLKCL--------

5Sdumerili NFFLLKCL--------

4Hburtoni NFFLLKCL--------

1aOniloticus NFFLLKCL--------

3Mzebra NFFLLKCL--------

2Aocellaris NFFLLKCL--------

7Aanguilla NSFLLRCL--------

Gaustralis2 NSFLLRCL--------

1Pmarinus NSFLLKCL--------

1Lreissneri NSFLLKCL--------

4Municolor NTYLLKCL--------

2Xtropicalis NTFLLKCL--------

2Acatesbeiana NTFLLKCL--------

2Bbufo NTFLLKCL--------

6Loculatus NSFLLKCL--------

2Ecalabaricus NSFLLKCL--------

1Hhuso NSFLLRCL--------

2Hhuso NSFLLRCL--------

1Lchalumnae NSFLLKCL--------

Sharrisii NSFLLKCL--------

Mdomestica NSFLLKCL--------

Dgliroides NSFLLKCL--------

3Cmilli NSFLLKCL--------

Oanatinus NSFLLKCL--------

Tlatirostris NSFLLKCL--------

Hsapiens NSFLLKCL--------

Btaurus NSFLLKCL--------

Mmusculus NSFLLKCL--------

Dnovemcintus NSFLLKCL--------

1Pmajor NSFLLKCL--------

1Ggallus NSFLLKCL--------

1cpb NSFLLKCL--------

1Pbivittatus NTFLLKCL--------

gekko1 NTFLLKCL--------

anole NTFLLKCL--------

6Drerio HFFILKCL--------

2Dclupeoides DLFILKCL--------

2Cchanos DFFIMKCL--------

1aSsenegalensis NFDVLKCV--------

3Csemilaevis NFTVLKCL--------

1Drerio SFSILKCL--------

1Cchanos NFSILKCL--------

1Dclupeoides SFSVMKCS--------

5Olatipes DFSILKCL--------

3cpb NFYILKCL--------

4Ssalar NFSILKCF--------

7Salpinus NFSILKCF--------

4Elucius NFTVLKCF--------

1Xmaculatus NFSVLQCA--------

4Aocellaris NFNVLKCL--------

5Hburtoni NFSVLKCL--------

4Mzebra NFSVLKCL--------

2bOniloticus NFSVLKCL--------

6Aanguilla NFSILKCL--------

gekko2 NMYILKCL--------

4Olatipes NFYVLKCL--------

3Hcomes NFYVLKCL--------

5Csemilaevis NFYVLKCL--------

3Hburtoni NFYVLKCL--------

2aOniloticus NFYVLKCL--------

6Mzebra NFYVLKCL--------

4Xmaculatus NFYVLKCL--------

2Saurata NFYVLKCL--------

1bSquinqueradiata NFYVLKCL--------

5Aocellaris NFYVLKCL--------

2Sdumerili NFYVLKCL--------

1bSsenegalensis NFYVLKCL--------

1Municolor NFYILKCL--------

1Xtropicalis NFYILKCL--------

1Acatesbeiana NFYILKCL--------

1Bbufo NFYILKCL--------

1Omordax NFYVLKCL--------

2Ssalar NFYVLKCL--------

3Ssalar NFYVLKCL--------

6Elucius NFYVLKCL--------

3Salpinus NFYVLKCL--------

1Aanguilla NFYILKCL--------

1Sformosus NFSILKCL--------

9Hhuso NFYILKCL--------

10Hhuso NFYILKCL--------

2Lchalumnae NFYILKCL--------

5Ecalabaricus NFYILKCL--------

1Loculatus NFYILKCL--------

. .*
