# Supplementary material for: Cocaine- and amphetamine-regulated transcripts in two percomorphs: evolutionary conservation and energy-status dependent responses
Source: Front Endocrinol (Lausanne). 2026 Jun 30;17:1870522. doi: 10.3389/fendo.2026.1870522 (PMC13364575; doi:10.3389/fendo.2026.1870522)
Supplement: Supplementary file 2 [file DataSheet2.docx]

all1_clean.muscle

6Bbufo LLELQDVLYKLQSKRSPAWESKYIQVPKCIMGDACAVKRGARIGKLCDCPPWSTCSLFF

6Acatesbeiana LLELQDVLEKLQSKRGILWESKLNQMPKCHHGDACAVKRGLRIGKLCDCPRRSVCNQFF

5Xtropicalis LTELQDVLEKLQSKRILSWESKLNQVPKCTLGDVCAVKRGARIGKLCDCPRRSNCNYYF

4Dclupeoides LGALQEVLEKLQTKRVPPWEKKLGQVPTCKFGEPCAVRKGARLGKMCECPPLTLCHPIV

1Cmilli LGALQEVLEKLQSKRLPTWEKKFGQLPLCDIGEQCAVRKGARIGKLCDCPRSTGCNFFL

4cbp LGALQEALEKLQKKRIPPWGKKLGQVPACDVGELCAVRKASRIGKLCNCPRGATCNFFL

2aSsenegalensis LGALHEVLEKLQTNRIHLWEKKYGQVPSCDLGEHCAVRKGSRIGKMCDCPRGASCHFLL

2Olatipes LGALHDVLERLQTTRINPWEKKYGQVPSCDLGEHCAIRKGSRIGKMCDCPRGAFCNFFL

6Huburtoni LGALHEVLERLQTKRINPWEKKYGQVPSCDLGEYCAIRKGSRIGKMCDCPRGAFCNFFL

1bOniloticus LGALHEVLERLQTKRINPWEKKYGQVPSCDLGEYCAIRKGSRIGKMCDCPRGAFCNFFL

5Msebra LGALHEVLERLQTKRINPWEKKYGQVPSCDLGEYCAIRKGSRIGKMCDCPRGAFCNFFL

3Aocellaris LGALHEVLERLQTKRINPWEKKYGQVPSCDLGEHCAVRKGSRIGKMCDCPRGAFCNFFL

1bSaurata LGALHEVLEKLQTKRINPWEKKYGQVPSCDLGEHCAVRKGSRIGKMCDCPRGAFCNFFL

2aSquinqueradiata LGALHEVLEKLQTKRINPWEKKYGQVPSCDLGEHCAVRKGSRIGKMCDCPRGAFCNFFL

1Sdumerili LGALHEVLEKLQTKRINPWEKKYGQVPSCDLGEHCAVRKGSRIGKMCDCPRGAFCNFFL

7Xmaculatus LGALHEVLEKLQTKRINPWEKKYGQVPSCDLGEHCAIRKGSRIGKMCDCPRGAFCNFFL

4Omordax LGALHDVLEKLQTKRISPWEKKFGQVPTCDMGEHCAVRKGARIGKMCDCPRGAFCNFFL

2Elucius LGALHEVLEKLQTKRINPWEKKFGQLPTCDLGEHCAVRKGARIGKMCDCPRGAFCNFFL

7Ssalar LGALHEVLKKLQTKRINPWEKKFGQVPTCDVGDHCAVRKGARIGKMCDCPRGAFCNFFL

9Ssalar LGALHEVLKKLQTKRINPWEKKFGQVPTCDVGDHCAVRKGARIGKMCDCPRGAFCNFFL

4Salpinus LGALHEVLKKLQTKRINPWEKKFGQVPTCDVGEHCAVRKGARIGKMCDCPRGAFCNFFL

2Municolor LGALQEVLEKLQSKRVPVWEKKFGQVPTCDIGEQCAVRKASRIGKLCNCPRGAVCNFFL

4Xtropicalis LGALQEVLEKLQNKRIPSWEKKFGQVPVCDVGEQCAVRKASRIGKLCNCPRGAVCNFFL

3Bbufo LGALQEVLEKLQSKRVPLWEKKFGQVPVCDMGEQCAVRKASRIGKLCNCPRGSVCNFFL

3Acatesbeiana LGALQEVLEKLQSKRVPAWEKKFGQVPVCDVGEQCAVRKASRIGKLCNCPRGAVCNFFL

3Drerio LGALHDVLEKLQSKRISLWEKKFGRVPTCDVGEQCAIRKGSRIGKMCDCPRGAFCNYFL

4Cchanos LGALHDVLEKLQSKRISLWEKKFGRVPLCDVGEQCAVRKGSRIGKMCDCPRGAFCNFFL

3Lchalumnae VRALQEVLEKLQSKRVPTWEKKFGQVPMCDVGEQCAVRKGARIGKLCDCPRGSICNFFL

3Dclupeoides LGALHEVLEKLQTKRIPPWEKKFGRVPTCDVGEQCAIRKGARIGKMCDCPRGAICNFFL

3Hhuso LGALQEVLEKLQSKRVPAWEKKFGQVPTCDVREQCAVRKGARIGKLCDCPRGAICNFFL

5Hhuso LGALQEVLEKLQSKRVPAWEKKFGQVPTCDVREQCAVRKGARIGKLCDCPRGAICNFFL

3Ecalabaricus LGALQEVLEKLQSKRVPAWEKKFGQVPTCDVGEQCAVRKGARIGKLCDCPRGAICNFFL

3Sformosus LGALQEVLEKLQTKRIPPWEKKFGQVPTCDVGKQCAVRKGARIGKMCDCPRGTFCNFFL

4Aanguilla LGALQDVLEKLQKKRIPSWEKKFGQVPTCDVGEQCAVRKGARIGKMCDCPRRAFCNFFL

3Cchanos LGALQEVLEKLQTKRIPPWEKKFGQVPMCDVGEQCAVRKGSRIGKMCDCPRGAFCNFFL

2Drerio LGALQEVLEKLQTKRIPPWEKKFGQVPMCDLGEQCAIRKGSRIGKMCDCPRGALCNFFL

3Omordax LGALQDVLEKLQTKRLSMWEKKFGQVPTCDVGEQCAVRKGARIGKMCDCPRGAFCNFFL

1Ssalar LGALHDVLKKLQTKRLPFWEKKFGQVPTCDVGEQCAVRKGARIGKMCDCPRGAFCNSYL

10Ssalar LGALHDVLKKLQTKRLPFWEKKFGQVPTCDVGEQCAVRKGARIGKMCDCPRGAFCNSYL

5Salpinus LGALHDVLKKLQTKRLPFWEKKFGQVPTCDVGEQCAVRKGARIGKMCDCPRGAFCNSYL

5Elucius LGALHDVLEKLQTKRLPFWEKKFGQVPTCDVGEQCAVRKGARIGKMCDCPRGAFCNFFL

2Aanguilla LGALQEVLEKLQSKRISPWEKKFGQVPTCDVGEQCAVRKGSRIGKMCDCPRGAFCNFFL

2Sformosus LGALQEVLEKLQSKRISPWEKKFGQVPTCDVGEQCAIRKGARIGKMCDCPRGAFCNFFL

2Loculatus LGALQEVLEKLQSKRIPTWEKKFGQVPTCDVGEQCAVRKGARIGKMCDCPRGAFCNFFL

5Hcomes LGALQEVLEKLQTKRLPMWEKKFGQVPTCDIGEQCAVRKGARIGKMCDCPRGAFCNSFL

3Olatipes LGALQEVLEKLQAKRLPSWEKKFGQVPMCDVGEQCAVRKGARIGKMCDCPRGAFCNFFL

3Xmaculatus LGALQEVLEKLQAKRLPSWEKKFGQVPTCDVGEQCAVRKGARIGKMCDCPRGAFCNFFL

2bSsenegalensis LGALQEVLEKLQTKRLPLWEKKFGQVPTCDVGEQCAVRKGARIGKMCDCPRGAFCNFFL

1aSaurata LGALQEVLEKLQAKRLPMWEKKFGQVPTCDVGEQCAVRKGARIGKMCDCPRGAFCNFFL

2bSquinqueradiata LGALQEVLEKLQAKRLPMWEKKFGQVPTCDVGEQCAVRKGARIGKMCDCPRGAFCNFFL

5Sdumerili LGALQEVLEKLQAKRLPMWEKKFGQVPTCDVGEQCAVRKGARIGKMCDCPRGAFCNFFL

4Hburtoni LGALQEVLEKLQAKRLPLWEKKFGQVPTCDIGEQCAVRKGARIGKMCDCPRGAFCNFFL

1aOniloticus LGALQEVLEKLQAKRLPLWEKKFGQVPTCDIGEQCAVRKGARIGKMCDCPRGAFCNFFL

3Mzebra LGALQEVLEKLQAKRLPLWEKKFGQVPTCDIGEQCAVRKGARIGKMCDCPRGAFCNFFL

2Aocellaris LGALQEVLEKLQAKRLPLWEKKFGQVPTCDVGEQCAVRKGARIGKMCDCPRGAFCNFFL

21Pbivittatus LEELQEVLEKLQHKKVSPWEKKFNQVPKCSFGDPCAIRKGARIGKLCDCPRRAACNAFL

gekko3 LEELQNVLEKLQHKTVSTWEKKFNLVPKCSFGDLCAVKKGARIGKLCDCPRGSACNTFL

6Lchalumnae LNALQGVLEKLQSKRILTWEKKFNQVPKCSIGNFCAVKKGARIGRLCDCPRWTSCNSFL

2cpb LEELQDVLEKLQSKRISTWEKKHNQVPKCSIGQACAVKKGARIGRLCDCPRGATCNTFL

7Sformosus LDVLRNVLEGLQKKRLSVLQRRYRRLPGCNVGDFCSVKRGARHGQLCDCPRGSKCNYFF

1Ecalabaricus LGALQEVLEKLQHRRLSTWDKKFSRVPRCAIGDYCSVKKGARFGKLCDCPQEASCNFFF

4Loculatus LGALQHVLEKLQNRRLGTWEKKLSRLPQCDIGDYCSVKKGARFGKLCDCPRGVKCNFFF

9Aanguilla LDVLHDVLEKLRNRRMAILERTHSRLPRCSVGDFCSVKKGARFGQLCDCPRGSKCNYFF

5Sformosus LDVLHNVLEKLQNRRMGGWERKTSRLPVCYIGDFCSVKKGSRFGQLCDCPRGSKCNFFF

6Dclupeoides LGVLQNVLEKLQNRRMTIWERKHSRLPNCNVGDFCTVKKGPRFGQLCDCPRGSKCNLFF

7Omordax LGVLHNVLEKLQNRRMASWERRQSRLPSCNVGDFCTAKKGPRFGQLCDCPRGSKCNHFF

8Salpinus LGVLHNVLEKLQNRRMAVWERRQSHLPSCIVGDYCTVKKGPRYSQLCDCPRGSKCNLFF

: *. .* *. :* * . *: ... * ..:*:** * .

6Bbufo MRCL

6Acatesbeiana LRCL

5Xtropicalis LRCL

4Dclupeoides LKCF

1Cmilli LKCL

4cbp LKCL

2aSsenegalensis LKCL

2Olatipes LKCL

6Huburtoni LKCL

1bOniloticus LKCL

5Msebra LKCL

3Aocellaris LKCL

1bSaurata LKCL

2aSquinqueradiata LKCL

1Sdumerili LKCL

7Xmaculatus LKCL

4Omordax LKCL

2Elucius LKCL

7Ssalar LKCL

9Ssalar LKCL

4Salpinus LKCL

2Municolor LKCL

4Xtropicalis LKCL

3Bbufo LKCL

3Acatesbeiana LKCL

3Drerio LKCL

4Cchanos LKCL

3Lchalumnae LKCL

3Dclupeoides LKCL

3Hhuso LKCL

5Hhuso LKCL

3Ecalabaricus LKCL

3Sformosus LKCL

4Aanguilla LKCL

3Cchanos LKCL

2Drerio LKCL

3Omordax LKCL

1Ssalar LKCL

10Ssalar LKCL

5Salpinus LKCL

5Elucius LKCL

2Aanguilla LKCL

2Sformosus LKCL

2Loculatus LKCL

5Hcomes LKCL

3Olatipes LKCL

3Xmaculatus LKCL

2bSsenegalensis LKCL

1aSaurata LKCL

2bSquinqueradiata LKCL

5Sdumerili LKCL

4Hburtoni LKCL

1aOniloticus LKCL

3Mzebra LKCL

2Aocellaris LKCL

21Pbivittatus LKCL

gekko3 LKCL

6Lchalumnae LKCL

2cpb LKCL

7Sformosus LKCL

1Ecalabaricus LKCL

4Loculatus LKCL

9Aanguilla LKCL

5Sformosus LKCL

6Dclupeoides LKCL

7Omordax LKCL

8Salpinus LKCL

:.*:

all2_clean.muscle

2Pmajor VEVLQEVLDKLRTREPPALEKRLSWVPWCEPREPCAVRRGARIGKLCSCPRGTSCNL

2Ggallus VEALQEVLEKLRSRELPPTAKKPGRVPSCHLGEPCAVRVGARYGKRCSCPPGTACNL

6Drerio IEALQEVLEKLKNKQLPQTGKKLSLLPSCDAGEQCAIRKGARVGKLCSCPQGTSCHF

2Dclupeoides IEALQEVLEKLKNKQMPKSARNFGMLPSCDAGEQCAIRKGARVGKLCGCPPGMACDL

2Cchanos IEALQEVLEKLKNKQMPNSGKKFGRLPSCDAGEQCAVRKGARVGKLCGCPQGTACDF

1aSsenegalensis IEALQEVLEKLKSKQLPSTEKKLGWLPSCDVGQQCALRKGSRIGKLCSCPGGNVCNF

3Csemilaevis IEALQDVLEKLKSKQLPSTEKKQSWLPSCDAGQLCALRKGSRIGKLCGCPTGTVCNF

1Drerio IEALQEVLEKLRNKQIPAVEKKLGWVPSCDAGEQCAVRKGSRFGKLCSCPGGTACSF

1Cchanos IEALQEVLEKLRNKDMPTTEKKFGWVPPCDAGEQCAVRKGARFGKLCSCSGGTTCNF

1Dclupeoides IEALQEVLERLKNTEKPPAEKKLGWVPSCDAGEPCAVRKGARIGKLCACPRGTSCSF

5Olatipes IEALQEVLEKLRNKQLPSSEKKLGWLPPCNTSEQCAVRKGARVGKLCGCPRGMECDF

3cpb IDALQEVLEKLKTERLPSIEKKLGSVASCDAGEPCAVRKGARIGRLCSCPRGTACNF

4Ssalar FEALQEVLEKLQSKQMPAYEKKLGWVPMCDAGQQCAVRKGARIGKLCECPRGTSCNF

7Salpinus FEALQEVLEKLQSKQMPAYEKKLGWVPMCDAGQQCAVRKGARIGKLCECPRGTSCNF

4Elucius FDALQEVLEKLKNKQTPSYEKKLGWVPMCDAGQQCAVRKGARIGKLCECPRGTSCNF

1Xmaculatus IEALQEVLEKLKNKQLPSSEKKLGWLPPCDAGEQCAVRKGARIGKLCGCPRGTLCNF

4Aocellaris IEALQEVLEKLKNKQLPSSEKKLGWLPACDAGEQCAVRKASRIGKLCGCPRGTVCNF

5Hburtoni IEALQEVLEKLKGKQLPSSEKKLGWLAACDAGEQCAIRKASRIGKLCGCPGGTACNF

4Mzebra IEALQEVLEKLKGKQLPSSEKKLGWLAACDAGEQCAIRKASRIGKLCGCPGGTACNF

2bOniloticus IEALQEVLEKLKGKQLPSSEKKLGWLAACDAGEQCAIRKASRIGKLCGCPGGTVCNF

6Aanguilla IEALQEVLEKLKNQRMPATEKKLGWVSSCDAGEECALRKGARIGKLCSCPRGTSCNF

gekko2 MEALQEVLEKLRSSRLPPLEKKLGWVPSCDAGESCAVRKGSRIGKLCNCPRRTSCNM

4Olatipes IDALQGVLEKLRNKEMP-LEKKLGWLPSCDAGEPCAVRKGARIGTLCGCPRGTSCNF

3Hcomes IDALQEVLEKLRNKEMP-SEKKLGWLPSCDAGEPCAVRKGSRIGTLCSCPRGTSCNF

5Csemilaevis IDALQEVLEKLRSKEMP-SEKKHGWLPSCDAGEPCALRKGARIGTLCSCPRGTSCNF

3Hburtoni IDALQEVLEKLRSKEMP-LEKKHGWLPSCDAGEPCAVRKGARIGTLCSCPRGTTCNF

2aOniloticus IDALQEVLEKLRSKEMP-LEKKHGWLPSCDAGEPCAVRKGARIGTLCSCPRGTTCNF

6Mzebra IDALQEVLEKLRSKEMP-LEKKHGWLPSCDAGEPCAVRKGARIGTLCSCPRGTTCNF

4Xmaculatus IDALQEVLEKLRNKEMP-LEKKLGWLPSCDAGEPCAVRKGARIGTLCSCPRGTACNF

2Saurata IDALQEVLEKLRSKEMP-LEKKLGWLPSCDAGEPCAVRKGARIGTLCSCPRGTSCNF

1bSquinqueradiata IDALQEVLEKLRSKEMP-LEKKLGWLPSCDAGEPCAVRKGARIGTLCSCPRGTSCNF

5Aocellaris IDALQEVLEKLRSKEMP-LEKKLGWLPSCDAGEPCAVRKGARIGTLCSCPRGTSCNF

2Sdumerili IDALQEVLEKLRSKEMP-LEKKLGWLPSCDAGEPCAVRKGARIGTLCSCPRGTSCNF

1bSsenegalensis IDALQEVLEKLRSKEMP-LEKKLGWLPSCDAGEPCAVRKGARIGTLCSCPRGTACNF

1Municolor IEALQDVLKKLESKRMPSLEKKLGWLPSCDAGEQCAVRKGARIGKLCSCPRGTACNF

1Xtropicalis IDALQEVLEKLKSKRLP-LDKKLGWVPSCDAGEQCAVRKGARIGKLCNCPRGTACNF

1Acatesbeiana IEALQDVLEKLKSKRLP-LDKKLGWVPSCDAGEQCAVRKGARIGKLCNCPRGTTCNF

1Bbufo IEALQEVLEKLKSKKLP-LDKKLGWVPSCDAGEQCAVRKGARIGKLCNCPRGTSCNF

1Omordax IEALQEVLEKLKNKQMPSSEKKLGWLPSCDAGEQCAVRKGARVGTLCGCPRGTSCNF

2Ssalar IEALQEVLEKLKNKQMPLSEKKLGWLPSCDAGEQCAVRKGARVGTLCGCPRGTTCNF

3Ssalar IEALQEVLEKLKNKQMPLSEKKLGWLPSCDAGEQCAVRKGARVGTLCGCPRGTTCNF

6Elucius IEALQEVLEKLKNKQIPLSEKKLSWLPSCDAGEQCAVRKGARVGTLCGCPRGTTCNF

3Salpinus IEALQEVLEKLKNKQMPLSEKKLSWLPSCDAGEKCAVRKGARVGTLCGCPRGTTCNF

1Aanguilla IEALQEVLEKLKNKQMPSAEKKLGWLPSCDAGEQCAIRKGARIGQLCGCPRGTSCNF

1Sformosus IEALQEVLEKLKNKQMPSAEKKLGWVPSCDAGEQCAIRKGARIGKLCNCPRGTSCNF

9Hhuso IEALQEVLEKLKSKRLPSAEKKLGWVPSCDAGEQCAVRKGSRIGKLCNCPRGTSCNF

10Hhuso IEALQEVLEKLKSKRLPSAEKKLGWVPSCDAGEQCAVRKGSRIGKLCNCPRGTSCNF

2Lchalumnae IEALQEVLEKLKNKRVPSAEKKLGWVPSCDAGEQCAVRKGARIGKLCNCPRGTSCNF

5Ecalabaricus IEALQEVLEKLKSKRMPSAEKKLGWVPSCDAGEQCAVRKGARIGKLCNCPRGTSCNF

1Loculatus IEALQEVLEKLKNKRMPSAEKKLGWVPSCDAGEQCAVRKGARIGKLCNCPRGTSCNF

.:.** **..* * .. . :. * : **:* .:* * * *. *

2Pmajor FILKCS

2Ggallus YVLRCS

6Drerio FILKCL

2Dclupeoides FILKCL

2Cchanos FIMKCL

1aSsenegalensis DVLKCV

3Csemilaevis TVLKCL

1Drerio SILKCL

1Cchanos SILKCL

1Dclupeoides SVMKCS

5Olatipes SILKCL

3cpb YILKCL

4Ssalar SILKCF

7Salpinus SILKCF

4Elucius TVLKCF

1Xmaculatus SVLQCA

4Aocellaris NVLKCL

5Hburtoni SVLKCL

4Mzebra SVLKCL

2bOniloticus SVLKCL

6Aanguilla SILKCL

gekko2 YILKCL

4Olatipes YVLKCL

3Hcomes YVLKCL

5Csemilaevis YVLKCL

3Hburtoni YVLKCL

2aOniloticus YVLKCL

6Mzebra YVLKCL

4Xmaculatus YVLKCL

2Saurata YVLKCL

1bSquinqueradiata YVLKCL

5Aocellaris YVLKCL

2Sdumerili YVLKCL

1bSsenegalensis YVLKCL

1Municolor YILKCL

1Xtropicalis YILKCL

1Acatesbeiana YILKCL

1Bbufo YILKCL

1Omordax YVLKCL

2Ssalar YVLKCL

3Ssalar YVLKCL

6Elucius YVLKCL

3Salpinus YVLKCL

1Aanguilla YILKCL

1Sformosus SILKCL

9Hhuso YILKCL

10Hhuso YILKCL

2Lchalumnae YILKCL

5Ecalabaricus YILKCL

1Loculatus YILKCL

::.*

all3.muscle

6Xtropicalis AAALEEMLDYN--QDKG-IRLQRRVGQLPWCDVGGRCAMKRGPRIGKLCDCLRGTSCNSF

5Acatesbeiana AVALGELLDYN--QDRG-LSLEKKASQLPRCDVGERCAMKHGPRIGKLCDCLRGASCSSF

5Bbufo AVALGEMLEYN--DPDGGVALEKKAVQVPRCDVGERCALKHGPRIGKLCDCLRGASCNSF

5Lchalumnae VEAINDILEND--HDRP-ISVEKKASQIPRCDVGERCAVKYGPRIGKLCDCLRGAACNTF

5Loculatus AEALEGLLDES-QDNRV--SVDKK-SLIPRCDVGERCAVKHGPRIGKLCDCLRGAACNTF

6Ecalabaricus AEVLEGLLENN--QDNA-IAVDKKASQIPRCDVGERCAMKHGPRIGKLCDCLRGAACNTF

7Hhuso VDALEGLLENS--SDTI--AVEKKANQIPRCDVGERCALKYGPRIGKLCDCLRGAACNTF

8Hhuso VDALEGLLENS--SDTI--AVEKKASQIPRCDVGERCALKYGPRIGKLCDCLRGAACNTF

3aSsenegalensis VEALQGVLGDS---DTLSLSVEKKASVIPRCDVGERCAMKHGPRIGRLCDCLRGTACNTF

4Csemilaevis QEALQSLLSDS---NAASLSVEKKAGVIPRCDVGERCAMKHGPRIGRLCDCLRGTACNTF

Amexicanus AEALEDMLDGD-EDNRI--QLEKKASVIPRCDVGERCALKHGPRIGRLCDCMRGTACNTF

5Dclupeoides AEALEDFLEGE-QDNRI--SVEKKASVIPRCDVGERCAMKHGPRIGRLCDCMRGTACNTF

4Drerio AEALDELLDGE-QDNRI--SLEKKASVIPRCDVGERCAMKHGPRIGRLCDCMRGTACNTF

6Sformosus AEALGGLLEGE-QDHRI--FLEKKASVIPRCDVGERCAMKHGPRIGRLCDCLRGTACNSF

5Aanguilla AEALEGLLDGT-QDNRI--TLEKKASVIPRCDVGERCAMKHGPRIGRLCDCLRGTACNSF

6Cchanos VDALEGLLEGD-QDNRI--SLEKKASVIPRCDVGERCAMKHGPRIGRLCDCLRGTACNTF

5Omordax ADALEGLLESG-QENSIGLSVEKKASVIPRCDVGERCAMKHGPRIGRLCDCMRGTACNTF

8Ssalar ADALERLLEGVQQDNRIGLSVEKKASLIPRCDVGERCAMKHGPRIGRLCDCLRGTACNTF

1Salpinus ADALERLLEGVQQDNRIGLSVEKKASLIPRCDVGERCAMKHGPRIGRLCDCLRGTACNTF

3Elucius ADALEGLLEDKQQDNMIGLSVEKKASLIPRCDVGERCAMKHGPRIGRLCDCLRGTACNTF

6Ssalar ADALEGLLEGGQQDNMIGLSVEKKASLIPRCDVGERCAMKHGPRIGRLCDCLRGTACNTF

2Salpinus ADALEGLLEGGQQDNMIGLSVEKKASLIPRCDVGERCAMKHGPRIGRLCDCLRGTACNTF

2Hcomes AEVLQGFLDEA-EGGGAGVSREKKASFIPRCDVGERCAMKHGPRIGRLCDCLRGTACNTF

S3bSquinqueradiata AEALQGFLDEA--DSRVGLSVEKKASVIPRCDVGERCAMKHGPRIGRLCDCLRGTACNTF

3Sdumerili AEALQGFLDEA--DSRVGLSVEKKASVIPRCDVGERCAMKHGPRIGRLCDCLRGTACNTF

2Xmaculatus AEALQGLLDEA--DSRVGLSVEKKASVIPRCDVGERCAMKHGPRIGRLCDCLRGTACNTF

7Aocellaris AEALQGLLDEA--DSRVGLSVEKKASVIPRCDVGERCAMKHGPRIGRLCDCLRGTACNTF

3aSaurata AEALQGFLDEA--DSSVGLSVEKKASVIPRCDVGERCAMKHGPRIGRLCDCLRGTACNTF

2Hburtoni AEALQGLLDEA--DSSAGLSVEKKASVIPRCDVGERCAMKHGPRIGRLCDCLRGTACNTF

3aOniloticus AEALQGLLDEA--DSSAGLSVEKKASVIPRCDVGERCAMKHGPRIGRLCDCLRGTACNTF

2Mzebra AEALQGLLDEA--DSSAGLSVEKKASVIPRCDVGERCAMKHGPRIGRLCDCLRGTACNTF

1Olatipes AEALQGLLDEA--DSSVGLSVEKKASVIPRCDVGERCAMKHGPRIGRLCDCLRGTACNTF

5Drerio VEAMTALLERY--QSHL-PSSEKR--AIPQCALGSRCAMRLGSRFGKLCECGRGSNCNSF

1Elucius VDAMEALLVKM--ESRL-PSTEKR-GMIPPCGVGQRCALRHGPHIGKLCDCGRVSSCNSF

5Ssalar VGAMEALIVKM--QSHL-PTNEKR-GMIPPCGMGDRCALRHGPRIGKLCDCGRVSSCNSF

6Salpinus VDAMEALLVKM--QSHL-PSNEKR-GMIRPCGMGDRCALRHGPRIGKLCDCGRVSSCNSF

5Cchanos IEAMEALLGKY--PER--PHAEEKRG-IPTCLTGSRCAVRLGPRIGKLCECGRGSNCNSF

6Omordax MDVMETLLGKM--NHRF-PSTDKR-GSIPICGMRDRCAMRLGPRIGKLCDCGRGGNCNSY

1Hburtoni LEALDVLLGRN--HNQV--SPEKR-GSIPLCGLGNRCAMKYGPRIGKLCDCGRGANCNSY

3bOniloticus LEALDVLLGRN--HNQV-SSPEKR-GSIPLCGLGNRCAMKYGPRIGKLCDCGRGANCNSY

1Mzebra LEALDVLLGRN--HNQV-SSPEKR-GSIPLCGLGNRCAMKYGPRIGKLCDCGRGANCNSY

1Aocellaris LEALEALLGRT--QNRV-PSTEKR-GSIPLCGMGDRCAMKFGPRIGKLCDCGRAANCNSY

6Xmaculatus VEALEVLLGRI--HSRV-SSTEKR-GSIPLCGMGGRCAVKFGPRIGKLCDCGRGANCNSY

1Csemilaevis IDALETLLGRM--HNRI--SYEKR-GSIPLCGMGDRCAMKYGPRIGKLCDCGRGANCNSY

4Hcomes VEALDVLLSRM--HNRI--STEKR-GNIPLCGMGDRCAMKFGPRIGKLCDCGRGANCNSY

3bSsenegalensis LEALEALLGRM--HNRI-SSTEKR-GSIPLCGMGERCAMKYGPRIGKLCDCGRAANCNSY

3aSquinqueradiata VEALEALLGRM--HNRI-SSTEKR-GSIPLCGMGDRCAMKFGPRIGKLCDCGRGANCNSY

4Sdumerili VEALEALLGRM--HNRI-SSTEKR-GSIPLCGMGDRCAMKFGPRIGKLCDCGRGANCNSY

3bSaurata VEALEALLGRM--HSRT-GSTEKR-GSIPLCGMGDRCAMKFGPRIGKLCDCGRGANCNSY

3Aanguilla LEAMEDLLGKF--QSRL-PSTEKR-GSIPLCGVGDRCAVRLGPRIGKLCDCAGRRNCNSF

2Cmilli FEAVEEILGKL--HNAISPSYEKKAGQIPKCDIGDRCAIKQGPRIGKLCDCARGTTCNSF

4Sformosus VEALEEILGKF--ENRV---PEKR-GSIPTCGRGERCAVKLGPRIGKLCDCGRGSHCNSF

4Lchalumnae VEAMEELLGKF--QNRY-PTYEKKGGQIPLCAIGERCAVKQGPRIGKLCDCSRGSSCNSF

4Bbufo VEAMEELLEKF--QDRY-PVYQKR-AQIPLCDIGERCAVKQGPRIGKLCDCSRGSSCNSF

3Xtropicalis VEAMEELLGKF--QDRY-PTYQKK-AQIPLCDIGERCAVKQGPRIGKLCDCSRGSSCNSF

3Municolor VEAMEELLGKF--QNKY-PSYQKKAAQIPMCDIGERCAVKQGPRIGKLCDCSRGAICNTF

4Acatesbeiana VEAMEELLGKS--------LYQKR-AQIPMCDIGERCAVKQGPRIGKLCDCSRGSSCNTF

4Ecalabaricus VETMEELLGKF--QSRL-PSYEKKGGTIPLCDVGDRCAVKLGPRIGKLCNCARGSSCNSF

3Loculatus VEAMEELLGKV--QSRF-PSYEKKAATIPMCDVGDRCALRQGPRIGKLCDCARGSICNSF

4Hhuso VEAMEELLGKF--QSRF-PSYEKKAGTIPLCDVGDRCAVKQGPRIGKLCDCARGSTCNSF

6Hhuso VEAMEELLGKF--QSRF-PSYEKKAGTIPLCDVGDRCAVKQGPRIGKLCDCARGSTCNSF

.: .: : . : * ***:. *..:*.**:* *.::

6Xtropicalis LLRCY

5Acatesbeiana MLRCY

5Bbufo MLRCY

5Lchalumnae LLRCY

5Loculatus LLRCY

6Ecalabaricus LLRCY

7Hhuso LLRCY

8Hhuso LLRCY

3aSsenegalensis FLRCY

4Csemilaevis FLRCY

Amexicanus FLRCY

5Dclupeoides FLRCY

4Drerio FLRCY

6Sformosus FLRCY

5Aanguilla FLRCY

6Cchanos FLRCY

5Omordax FLRCY

8Ssalar FLRCY

1Salpinus FLRCY

3Elucius FLRCY

6Ssalar FLRCY

2Salpinus FLRCY

2Hcomes FLRCY

S3bSquinqueradiata FLRCY

3Sdumerili FLRCY

2Xmaculatus FLRCY

7Aocellaris FLRCY

3aSaurata FLRCY

2Hburtoni FLRCY

3aOniloticus FLRCY

2Mzebra FLRCY

1Olatipes FLRCY

5Drerio LLKCI

1Elucius LLKCL

5Ssalar LLKCL

6Salpinus LLKCL

5Cchanos LLKCI

6Omordax LLKCI

1Hburtoni LLKCI

3bOniloticus LLKCI

1Mzebra LLKCI

1Aocellaris LLKCI

6Xmaculatus LLKCI

1Csemilaevis LLKCI

4Hcomes LLKCI

3bSsenegalensis LLKCI

3aSquinqueradiata LLKCI

4Sdumerili LLKCI

3bSaurata LLKCI

3Aanguilla LLKCI

2Cmilli LLKCI

4Sformosus LLKCI

4Lchalumnae LLKCI

4Bbufo LLKCI

3Xtropicalis LLKCI

3Municolor LLKCI

4Acatesbeiana LLKCI

4Ecalabaricus LLKCI

3Loculatus LLKCI

4Hhuso LLKCI

6Hhuso LLKCI

:*.*

4.muscle

7Aanguilla ISALKGVLEKLKNNRFPLYGKKYGQLPMCEAGERCALRKGARIGKLCDCPYRISCNSFLL

4Municolor LEALQEVLEKLKSKRIPSYEKKYGQVPMCEAGDQCAVRKGPRIGKLCDCPRRTSCNTYLL

2Ecalabaricus IDALQEVLEKLKNKRIPYYEKKIGQLPMCDAGDQCAVRKGARIGKLCDCPRGTSCNSFLL

6Loculatus IEALQEVLEKLKNKGMPFYGKKYGQLPMCEAGEQCALRKGARIGKLCDCPRATSCNSFLL

1Hhuso IEALQEVLEKLKSKRMPYYEKKYGQLPMCDAGEQCALRKGARIGKLCDCPRGTSCNSFLL

2Hhuso IEALQEVLEKLKSKRMPYYEKKYGQLPMCDAGEQCALRKGARIGKLCDCPRGTSCNSFLL

1Ggallus IEALQEVLEKLKSKRVPHYEKKFGQVPMCDAGEQCAVRKGARIGKLCDCPRGTSCNSFLL

1Pmajor IEALQEVLEKLKSKRGPHYEKKFGQVPMCDAGEQCAVRKGARIGKLCDCPRGTSCNSFLL

anole IEALQEVLEKLKSKRLPLYEKKYGQVPMCDAGEQCALRKGARIGKLCDCPRGTSCNTFLL

1Pbivittatus IEALQEVLEKLKSKRLPHYEKKYGQVPMCDAGEQCAVRKGARIGKLCDCPRGTSCNTFLL

gekko1 IEALQEVLEKLKSKRLPHYEKKYGQVPMCDAGEQCAVRKGARIGKLCDCPRGTSCNTFLL

2Xtropicalis IDALQEVLEKLKNKRLPLFEKKYGQVPMCDAGEQCAVRKGPRIGKLCDCPRRTSCNTFLL

2Acatesbeiana IDALQEVLEKLKNKRLPLFEKKYGQVPMCDAGEQCAVRKGPRIGKLCDCPRRTSCNTFLL

2Bbufo INALQEVLEKLKSKKLPSFEKKYGQVPMCDAGEQCAVRKGARIGRLCDCPRRTSCNTFLL

2Omordax INDLQGVLERLKNKRFLPHAKKHSLLPMCDAGEQCALRKGARIGKLCDCQQPRACSSFML

6Olatipes ISDLHQVLERLQSIQFPALRKKHGYLPVCEPGEQCALRKGSRIGKLCDCSLPRTCNSFLH

1Hcomes INDLHEVLDRLQKNQLSALRKKHGHLPMCDPGDQCALRRGSRIGKLCDCSLPRTCNSFLH

4Ssenegalensis ISDLHEVLEKLQYNQYPSLRKKHGYLPLCDPGDLCALRKGSRIGKLCDCPLPRTCNSFLH

5Xmaculatus INDLHQVLERLQHNQFPALRKKHSYLPACDPGEQCALRKGSRIGKLCDCSLPRTCNSFLH

7Hburtoni ISDLHDVLERLQNHPFPVLRKKHGYLPVCDPGEQCALRKGSRIGKLCDCSLPRTCNSFLH

7Mzebra ISDLHDVLERLQNHPFPVLRKKHGYLPVCDPGEQCALRKGSRIGKLCDCSLPRTCNSFLH

1cOniloticus INDLHEVLERLQNHPFPVLRKKHGYLPVCDPGEQCALRKGSRIGKLCDCSLPRTCNSFLH

1cSaurata INDLHEVLERLQNNQFPALRKKHGYLPVCDPGDQCALRRGSRIGKLCDCSPPRTCNSFLH

8Aocellaris INDLHEVLERLQNNQFPALRKKHGFLPVCDPGDQCALRKGSRIGKLCDCSLPRTCNSFLH

6Sdumerili INDLHEVLERLQNNQFPALRKKHGYLPLCDPGDLCALRKGSRIGKLCDCSLPRTCNSFLH

nSquinqueradiata INDLHEVLERLQNNQFPALRKKHGYLPLCDPGDLCALRKGSRIGKLCDCSPPRTCNSFLH

:. *: **:.*: ** . :* *:.*: **:*.*.***.**** :*.:::

7Aanguilla RCL

4Municolor KCL

2Ecalabaricus KCL

6Loculatus KCL

1Hhuso RCL

2Hhuso RCL

1Ggallus KCL

1Pmajor KCL

anole KCL

1Pbivittatus KCL

gekko1 KCL

2Xtropicalis KCL

2Acatesbeiana KCL

2Bbufo KCL

2Omordax RCL

6Olatipes RCF

1Hcomes RCL

4Ssenegalensis RCL

5Xmaculatus RCL

7Hburtoni RCL

7Mzebra RCL

1cOniloticus RCL

1cSaurata RCL

8Aocellaris RCL

6Sdumerili RCL

nSquinqueradiata RCL

.*:

Mammalian.muscle

1Lchalumnae IEALQEVLEKLKSKRIPVYEKKYNQVPMCDAGEQCALRKGSRIGKLCDCPRGTSCNSFLL

Sharrisii IGALQEVLKKLKSKRIRIYEKKYGQVPKCDAGEQCAIRKGARIGKLCDCPRGTSCNSFLL

Mdomestica IGALQEVLKKLKSKRIRIYEKKYGQVPKCDAGEQCAIRKGARIGKLCDCPRGTSCNSFLL

Dgliroides IGALQEVLKKLKSKRIRIYEKKYGQVPKCDAGEQCAIRKGARIGKLCDCPRGTSCNSFLL

1cpb IEALQEVLEKLKSKRIPVYEKKFGQVPMCDAGEQCAVRKGARIGKLCDCPRGTSCNSFLL

Oanatinus IEALQEVLKKLKSKRIPVYEKKYSQVPMCDAGEQCAVRKGARIGKLCDCPRGTACNSFLL

Tlatirostris IEALQEVLKKLKSKRIPIYEKKYGQVPMCDAGEQCAVRKGARIGKLCDCPRATSCNSFLL

Hsapiens IEALQEVLKKLKSKRVPIYEKKYGQVPMCDAGEQCAVRKGARIGKLCDCPRGTSCNSFLL

Btaurus IEALQEVLKKLKSKRIPIYEKKYGQVPMCDAGEQCAVRKGARIGKLCDCPRGTSCNSFLL

Mmusculus IEALQEVLKKLKSKRIPIYEKKYGQVPMCDAGEQCAVRKGARIGKLCDCPRGTSCNSFLL

Dnovemcintus IEALQEVLKKLKSKRIPIYEKKYGQVPMCDAGEQCAVRKGARIGKLCDCPRGTSCNSFLL

* ******:******: :****:.*** ********:***:**********.*:******

1Lchalumnae KCL

Sharrisii KCL

Mdomestica KCL

Dgliroides KCL

1cpb KCL

Oanatinus KCL

Tlatirostris KCL

Hsapiens KCL

Btaurus KCL

Mmusculus KCL

Dnovemcintus KCL

***

hag_lamp.muscle

Eburgeri LEALQQVLEKLQNKRVPTWQKKFGQTPMCSFGGRCALRRGPRIGKLCDCPQGTSCSSYLL

Mglutinosa LEALQEVLEKLQNKRVPTWQKKYGQTPMCSFGGRCALRRGPRIGKLCDCPQGTSCSSFLL

1Pmarinus LDALQSVLEKLQNKRMPSWEKKYGQLPICDAGEWCAVRKGARIGKLCDCTRGTSCNSFLL

1Lreissneri LDALQSVLEKLQNKRMPSWEKKYGQLPICDAGEWCAVRKGSRIGKLCDCTRGTSCNSFLL

Gaustralis2 LDALQSVLEKLQSKRMPNWEKKYGQLPICDAGEWCAVRKGARIGKLCDCTRGTSCNSFLL

2Pmarinus LEALQEVLEKLQSKRIPTWEKKFGQVALCGAGDQCAVRKGARIGKLCDCPRSYACNSFLL

2Lreissneri LEALQEVLEKLQSKRIPTWEKKFGQVALCGAGDQCAVRKGARIGKLCDCPRSYACNSFLL

Gaustralis1 LEALQEVLVKLQSKRIPTWEKKFGQVAVCGAGEQCAVRKGARIGKLCDCPRGAACNSFLL

*:***.** ***.**:*.*:**:** .:*. * .**:*.*.********... :*.*:**

Eburgeri KCL

Mglutinosa KCL

1Pmarinus KCL

1Lreissneri KCL

Gaustralis2 RCL

2Pmarinus RCL

2Lreissneri RCL

Gaustralis1 RCL

.**

1a_clean.muscle

7Sformosus LDVLRNVLEGLQKKRLSVLQRRYRRLPGCNVGDFCSVKRGARHGQLCDCPRGSKCNYFF

1Ecalabaricus LGALQEVLEKLQHRRLSTWDKKFSRVPRCAIGDYCSVKKGARFGKLCDCPQEASCNFFF

4Loculatus LGALQHVLEKLQNRRLGTWEKKLSRLPQCDIGDYCSVKKGARFGKLCDCPRGVKCNFFF

9Aanguilla LDVLHDVLEKLRNRRMAILERTHSRLPRCSVGDFCSVKKGARFGQLCDCPRGSKCNYFF

5Sformosus LDVLHNVLEKLQNRRMGGWERKTSRLPVCYIGDFCSVKKGSRFGQLCDCPRGSKCNFFF

6Dclupeoides LGVLQNVLEKLQNRRMTIWERKHSRLPNCNVGDFCTVKKGPRFGQLCDCPRGSKCNLFF

7Omordax LGVLHNVLEKLQNRRMASWERRQSRLPSCNVGDFCTAKKGPRFGQLCDCPRGSKCNHFF

8Salpinus LGVLHNVLEKLQNRRMAVWERRQSHLPSCIVGDYCTVKKGPRYSQLCDCPRGSKCNLFF

21Pbivittatus LEELQEVLEKLQHKKVSPWEKKFNQVPKCSFGDPCAIRKGARIGKLCDCPRRAACNAFL

gekko3 LEELQNVLEKLQHKTVSTWEKKFNLVPKCSFGDLCAVKKGARIGKLCDCPRGSACNTFL

6Lchalumnae LNALQGVLEKLQSKRILTWEKKFNQVPKCSIGNFCAVKKGARIGRLCDCPRWTSCNSFL

2cpb LEELQDVLEKLQSKRISTWEKKHNQVPKCSIGQACAVKKGARIGRLCDCPRGATCNTFL

6Bbufo LLELQDVLYKLQSKRSPAWESKYIQVPKCIMGDACAVKRGARIGKLCDCPPWSTCSLFF

6Acatesbeiana LLELQDVLEKLQSKRGILWESKLNQMPKCHHGDACAVKRGLRIGKLCDCPRRSVCNQFF

5Xtropicalis LTELQDVLEKLQSKRILSWESKLNQVPKCTLGDVCAVKRGARIGKLCDCPRRSNCNYYF

* *. ** *. . : :* * *: *: ..* * ..***** *. ::

7Sformosus LKCL

1Ecalabaricus LKCL

4Loculatus LKCL

9Aanguilla LKCL

5Sformosus LKCL

6Dclupeoides LKCL

7Omordax LKCL

8Salpinus LKCL

21Pbivittatus LKCL

gekko3 LKCL

6Lchalumnae LKCL

2cpb LKCL

6Bbufo MRCL

6Acatesbeiana LRCL

5Xtropicalis LRCL

:.**

1b.muscle

4Dclupeoides LGALQEVLEKLQTKRVPPWEKKLGQVPTCKFGEPCAVRKGARLGKMCECPPLTLCHPIVL

1Cmilli LGALQEVLEKLQSKRLPTWEKKFGQLPLCDIGEQCAVRKGARIGKLCDCPRSTGCNFFLL

4cbp LGALQEALEKLQKKRIPPWGKKLGQVPACDVGELCAVRKASRIGKLCNCPRGATCNFFLL

2aSsenegalensis LGALHEVLEKLQTNRIHLWEKKYGQVPSCDLGEHCAVRKGSRIGKMCDCPRGASCHFLLL

2Olatipes LGALHDVLERLQTTRINPWEKKYGQVPSCDLGEHCAIRKGSRIGKMCDCPRGAFCNFFLL

6Huburtoni LGALHEVLERLQTKRINPWEKKYGQVPSCDLGEYCAIRKGSRIGKMCDCPRGAFCNFFLL

1bOniloticus LGALHEVLERLQTKRINPWEKKYGQVPSCDLGEYCAIRKGSRIGKMCDCPRGAFCNFFLL

5Msebra LGALHEVLERLQTKRINPWEKKYGQVPSCDLGEYCAIRKGSRIGKMCDCPRGAFCNFFLL

3Aocellaris LGALHEVLERLQTKRINPWEKKYGQVPSCDLGEHCAVRKGSRIGKMCDCPRGAFCNFFLL

1bSaurata LGALHEVLEKLQTKRINPWEKKYGQVPSCDLGEHCAVRKGSRIGKMCDCPRGAFCNFFLL

2aSquinqueradiata LGALHEVLEKLQTKRINPWEKKYGQVPSCDLGEHCAVRKGSRIGKMCDCPRGAFCNFFLL

1Sdumerili LGALHEVLEKLQTKRINPWEKKYGQVPSCDLGEHCAVRKGSRIGKMCDCPRGAFCNFFLL

7Xmaculatus LGALHEVLEKLQTKRINPWEKKYGQVPSCDLGEHCAIRKGSRIGKMCDCPRGAFCNFFLL

4Omordax LGALHDVLEKLQTKRISPWEKKFGQVPTCDMGEHCAVRKGARIGKMCDCPRGAFCNFFLL

2Elucius LGALHEVLEKLQTKRINPWEKKFGQLPTCDLGEHCAVRKGARIGKMCDCPRGAFCNFFLL

7Ssalar LGALHEVLKKLQTKRINPWEKKFGQVPTCDVGDHCAVRKGARIGKMCDCPRGAFCNFFLL

9Ssalar LGALHEVLKKLQTKRINPWEKKFGQVPTCDVGDHCAVRKGARIGKMCDCPRGAFCNFFLL

4Salpinus LGALHEVLKKLQTKRINPWEKKFGQVPTCDVGEHCAVRKGARIGKMCDCPRGAFCNFFLL

2Municolor LGALQEVLEKLQSKRVPVWEKKFGQVPTCDIGEQCAVRKASRIGKLCNCPRGAVCNFFLL

4Xtropicalis LGALQEVLEKLQNKRIPSWEKKFGQVPVCDVGEQCAVRKASRIGKLCNCPRGAVCNFFLL

3Bbufo LGALQEVLEKLQSKRVPLWEKKFGQVPVCDMGEQCAVRKASRIGKLCNCPRGSVCNFFLL

3Acatesbeiana LGALQEVLEKLQSKRVPAWEKKFGQVPVCDVGEQCAVRKASRIGKLCNCPRGAVCNFFLL

3Drerio LGALHDVLEKLQSKRISLWEKKFGRVPTCDVGEQCAIRKGSRIGKMCDCPRGAFCNYFLL

4Cchanos LGALHDVLEKLQSKRISLWEKKFGRVPLCDVGEQCAVRKGSRIGKMCDCPRGAFCNFFLL

3Lchalumnae VRALQEVLEKLQSKRVPTWEKKFGQVPMCDVGEQCAVRKGARIGKLCDCPRGSICNFFLL

3Dclupeoides LGALHEVLEKLQTKRIPPWEKKFGRVPTCDVGEQCAIRKGARIGKMCDCPRGAICNFFLL

3Hhuso LGALQEVLEKLQSKRVPAWEKKFGQVPTCDVREQCAVRKGARIGKLCDCPRGAICNFFLL

5Hhuso LGALQEVLEKLQSKRVPAWEKKFGQVPTCDVREQCAVRKGARIGKLCDCPRGAICNFFLL

3Ecalabaricus LGALQEVLEKLQSKRVPAWEKKFGQVPTCDVGEQCAVRKGARIGKLCDCPRGAICNFFLL

3Sformosus LGALQEVLEKLQTKRIPPWEKKFGQVPTCDVGKQCAVRKGARIGKMCDCPRGTFCNFFLL

4Aanguilla LGALQDVLEKLQKKRIPSWEKKFGQVPTCDVGEQCAVRKGARIGKMCDCPRRAFCNFFLL

3Cchanos LGALQEVLEKLQTKRIPPWEKKFGQVPMCDVGEQCAVRKGSRIGKMCDCPRGAFCNFFLL

2Drerio LGALQEVLEKLQTKRIPPWEKKFGQVPMCDLGEQCAIRKGSRIGKMCDCPRGALCNFFLL

3Omordax LGALQDVLEKLQTKRLSMWEKKFGQVPTCDVGEQCAVRKGARIGKMCDCPRGAFCNFFLL

1Ssalar LGALHDVLKKLQTKRLPFWEKKFGQVPTCDVGEQCAVRKGARIGKMCDCPRGAFCNSYLL

10Ssalar LGALHDVLKKLQTKRLPFWEKKFGQVPTCDVGEQCAVRKGARIGKMCDCPRGAFCNSYLL

5Salpinus LGALHDVLKKLQTKRLPFWEKKFGQVPTCDVGEQCAVRKGARIGKMCDCPRGAFCNSYLL

5Elucius LGALHDVLEKLQTKRLPFWEKKFGQVPTCDVGEQCAVRKGARIGKMCDCPRGAFCNFFLL

2Aanguilla LGALQEVLEKLQSKRISPWEKKFGQVPTCDVGEQCAVRKGSRIGKMCDCPRGAFCNFFLL

2Sformosus LGALQEVLEKLQSKRISPWEKKFGQVPTCDVGEQCAIRKGARIGKMCDCPRGAFCNFFLL

2Loculatus LGALQEVLEKLQSKRIPTWEKKFGQVPTCDVGEQCAVRKGARIGKMCDCPRGAFCNFFLL

5Hcomes LGALQEVLEKLQTKRLPMWEKKFGQVPTCDIGEQCAVRKGARIGKMCDCPRGAFCNSFLL

3Olatipes LGALQEVLEKLQAKRLPSWEKKFGQVPMCDVGEQCAVRKGARIGKMCDCPRGAFCNFFLL

3Xmaculatus LGALQEVLEKLQAKRLPSWEKKFGQVPTCDVGEQCAVRKGARIGKMCDCPRGAFCNFFLL

2bSsenegalensis LGALQEVLEKLQTKRLPLWEKKFGQVPTCDVGEQCAVRKGARIGKMCDCPRGAFCNFFLL

1aSaurata LGALQEVLEKLQAKRLPMWEKKFGQVPTCDVGEQCAVRKGARIGKMCDCPRGAFCNFFLL

2bSquinqueradiata LGALQEVLEKLQAKRLPMWEKKFGQVPTCDVGEQCAVRKGARIGKMCDCPRGAFCNFFLL

5Sdumerili LGALQEVLEKLQAKRLPMWEKKFGQVPTCDVGEQCAVRKGARIGKMCDCPRGAFCNFFLL

4Hburtoni LGALQEVLEKLQAKRLPLWEKKFGQVPTCDIGEQCAVRKGARIGKMCDCPRGAFCNFFLL

1aOniloticus LGALQEVLEKLQAKRLPLWEKKFGQVPTCDIGEQCAVRKGARIGKMCDCPRGAFCNFFLL

3Mzebra LGALQEVLEKLQAKRLPLWEKKFGQVPTCDIGEQCAVRKGARIGKMCDCPRGAFCNFFLL

2Aocellaris LGALQEVLEKLQAKRLPLWEKKFGQVPTCDVGEQCAVRKGARIGKMCDCPRGAFCNFFLL

: **::.*:.** .*: * ** *.:* *.. . **:**.:*:**:*:** : *: :*

4Dclupeoides KCF

1Cmilli KCL

4cbp KCL

2aSsenegalensis KCL

2Olatipes KCL

6Huburtoni KCL

1bOniloticus KCL

5Msebra KCL

3Aocellaris KCL

1bSaurata KCL

2aSquinqueradiata KCL

1Sdumerili KCL

7Xmaculatus KCL

4Omordax KCL

2Elucius KCL

7Ssalar KCL

9Ssalar KCL

4Salpinus KCL

2Municolor KCL

4Xtropicalis KCL

3Bbufo KCL

3Acatesbeiana KCL

3Drerio KCL

4Cchanos KCL

3Lchalumnae KCL

3Dclupeoides KCL

3Hhuso KCL

5Hhuso KCL

3Ecalabaricus KCL

3Sformosus KCL

4Aanguilla KCL

3Cchanos KCL

2Drerio KCL

3Omordax KCL

1Ssalar KCL

10Ssalar KCL

5Salpinus KCL

5Elucius KCL

2Aanguilla KCL

2Sformosus KCL

2Loculatus KCL

5Hcomes KCL

3Olatipes KCL

3Xmaculatus KCL

2bSsenegalensis KCL

1aSaurata KCL

2bSquinqueradiata KCL

5Sdumerili KCL

4Hburtoni KCL

1aOniloticus KCL

3Mzebra KCL

2Aocellaris KCL

**:

2a_clean.muscle

2Pmajor VEVLQEVLDKLRTRE-PPALEKRLSWVPWCEPREPCAVRRGARIGKLCSCPRGTSCNL

2Ggallus VEALQEVLEKLRSRE-LPPTAKKPGRVPSCHLGEPCAVRVGARYGKRCSCPPGTACNL

4Ssalar FEALQEVLEKLQSKQ-MPAYEKKLGWVPMCDAGQQCAVRKGARIGKLCECPRGTSCNF

7Salpinus FEALQEVLEKLQSKQ-MPAYEKKLGWVPMCDAGQQCAVRKGARIGKLCECPRGTSCNF

4Elucius FDALQEVLEKLKNKQ-TPSYEKKLGWVPMCDAGQQCAVRKGARIGKLCECPRGTSCNF

3cpb IDALQEVLEKLKTER-LPSIEKKLGSVASCDAGEPCAVRKGARIGRLCSCPRGTACNF

gekko2 MEALQEVLEKLRSSRLLPPLEKKLGWVPSCDAGESCAVRKGSRIGKLCNCPRRTSCNM

1Xtropicalis IDALQEVLEKLKSKRILP-LDKKLGWVPSCDAGEQCAVRKGARIGKLCNCPRGTACNF

1Acatesbeiana IEALQDVLEKLKSKRILP-LDKKLGWVPSCDAGEQCAVRKGARIGKLCNCPRGTTCNF

1Bbufo IEALQEVLEKLKSKKILP-LDKKLGWVPSCDAGEQCAVRKGARIGKLCNCPRGTSCNF

1Municolor IEALQDVLKKLESKR-MPSLEKKLGWLPSCDAGEQCAVRKGARIGKLCSCPRGTACNF

4Olatipes IDALQGVLEKLRNKE-MP-LEKKLGWLPSCDAGEPCAVRKGARIGTLCGCPRGTSCNF

3Hcomes IDALQEVLEKLRNKE-MP-SEKKLGWLPSCDAGEPCAVRKGSRIGTLCSCPRGTSCNF

5Csemilaevis IDALQEVLEKLRSKE-MP-SEKKHGWLPSCDAGEPCALRKGARIGTLCSCPRGTSCNF

3Hburtoni IDALQEVLEKLRSKE-MP-LEKKHGWLPSCDAGEPCAVRKGARIGTLCSCPRGTTCNF

2aOniloticus IDALQEVLEKLRSKE-MP-LEKKHGWLPSCDAGEPCAVRKGARIGTLCSCPRGTTCNF

6Mzebra IDALQEVLEKLRSKE-MP-LEKKHGWLPSCDAGEPCAVRKGARIGTLCSCPRGTTCNF

4Xmaculatus IDALQEVLEKLRNKE-MP-LEKKLGWLPSCDAGEPCAVRKGARIGTLCSCPRGTACNF

2Saurata IDALQEVLEKLRSKE-MP-LEKKLGWLPSCDAGEPCAVRKGARIGTLCSCPRGTSCNF

1bSquinqueradiata IDALQEVLEKLRSKE-MP-LEKKLGWLPSCDAGEPCAVRKGARIGTLCSCPRGTSCNF

5Aocellaris IDALQEVLEKLRSKE-MP-LEKKLGWLPSCDAGEPCAVRKGARIGTLCSCPRGTSCNF

2Sdumerili IDALQEVLEKLRSKE-MP-LEKKLGWLPSCDAGEPCAVRKGARIGTLCSCPRGTSCNF

1bSsenegalensis IDALQEVLEKLRSKE-MP-LEKKLGWLPSCDAGEPCAVRKGARIGTLCSCPRGTACNF

.:.** **.** . * *. . :. * : **:* *:* * * ** *:**:

2Pmajor FILKCS

2Ggallus YVLRCS

4Ssalar SILKCF

7Salpinus SILKCF

4Elucius TVLKCF

3cpb YILKCL

gekko2 YILKCL

1Xtropicalis YILKCL

1Acatesbeiana YILKCL

1Bbufo YILKCL

1Municolor YILKCL

4Olatipes YVLKCL

3Hcomes YVLKCL

5Csemilaevis YVLKCL

3Hburtoni YVLKCL

2aOniloticus YVLKCL

6Mzebra YVLKCL

4Xmaculatus YVLKCL

2Saurata YVLKCL

1bSquinqueradiata YVLKCL

5Aocellaris YVLKCL

2Sdumerili YVLKCL

1bSsenegalensis YVLKCL

:*.*

2b.muscle

1aSsenegalensis IEALQEVLEKLKSKQLPSTEKKLGWLPSCDVGQQCALRKGSRIGKLCSCPGGNVCNFDVL

3Csemilaevis IEALQDVLEKLKSKQLPSTEKKQSWLPSCDAGQLCALRKGSRIGKLCGCPTGTVCNFTVL

6Drerio IEALQEVLEKLKNKQLPQTGKKLSLLPSCDAGEQCAIRKGARVGKLCSCPQGTSCHFFIL

2Dclupeoides IEALQEVLEKLKNKQMPKSARNFGMLPSCDAGEQCAIRKGARVGKLCGCPPGMACDLFIL

2Cchanos IEALQEVLEKLKNKQMPNSGKKFGRLPSCDAGEQCAVRKGARVGKLCGCPQGTACDFFIM

1Dclupeoides IEALQEVLERLKNTEKPPAEKKLGWVPSCDAGEPCAVRKGARIGKLCACPRGTSCSFSVM

1Drerio IEALQEVLEKLRNKQIPAVEKKLGWVPSCDAGEQCAVRKGSRFGKLCSCPGGTACSFSIL

1Cchanos IEALQEVLEKLRNKDMPTTEKKFGWVPPCDAGEQCAVRKGARFGKLCSCSGGTTCNFSIL

5Olatipes IEALQEVLEKLRNKQLPSSEKKLGWLPPCNTSEQCAVRKGARVGKLCGCPRGMECDFSIL

1Xmaculatus IEALQEVLEKLKNKQLPSSEKKLGWLPPCDAGEQCAVRKGARIGKLCGCPRGTLCNFSVL

4Aocellaris IEALQEVLEKLKNKQLPSSEKKLGWLPACDAGEQCAVRKASRIGKLCGCPRGTVCNFNVL

5Hburtoni IEALQEVLEKLKGKQLPSSEKKLGWLAACDAGEQCAIRKASRIGKLCGCPGGTACNFSVL

4Mzebra IEALQEVLEKLKGKQLPSSEKKLGWLAACDAGEQCAIRKASRIGKLCGCPGGTACNFSVL

2bOniloticus IEALQEVLEKLKGKQLPSSEKKLGWLAACDAGEQCAIRKASRIGKLCGCPGGTVCNFSVL

6Aanguilla IEALQEVLEKLKNQRMPATEKKLGWVSSCDAGEECALRKGARIGKLCSCPRGTSCNFSIL

1Omordax IEALQEVLEKLKNKQMPSSEKKLGWLPSCDAGEQCAVRKGARVGTLCGCPRGTSCNFYVL

2Ssalar IEALQEVLEKLKNKQMPLSEKKLGWLPSCDAGEQCAVRKGARVGTLCGCPRGTTCNFYVL

3Ssalar IEALQEVLEKLKNKQMPLSEKKLGWLPSCDAGEQCAVRKGARVGTLCGCPRGTTCNFYVL

6Elucius IEALQEVLEKLKNKQIPLSEKKLSWLPSCDAGEQCAVRKGARVGTLCGCPRGTTCNFYVL

3Salpinus IEALQEVLEKLKNKQMPLSEKKLSWLPSCDAGEKCAVRKGARVGTLCGCPRGTTCNFYVL

1Aanguilla IEALQEVLEKLKNKQMPSAEKKLGWLPSCDAGEQCAIRKGARIGQLCGCPRGTSCNFYIL

1Sformosus IEALQEVLEKLKNKQMPSAEKKLGWVPSCDAGEQCAIRKGARIGKLCNCPRGTSCNFSIL

9Hhuso IEALQEVLEKLKSKRLPSAEKKLGWVPSCDAGEQCAVRKGSRIGKLCNCPRGTSCNFYIL

10Hhuso IEALQEVLEKLKSKRLPSAEKKLGWVPSCDAGEQCAVRKGSRIGKLCNCPRGTSCNFYIL

2Lchalumnae IEALQEVLEKLKNKRVPSAEKKLGWVPSCDAGEQCAVRKGARIGKLCNCPRGTSCNFYIL

5Ecalabaricus IEALQEVLEKLKSKRMPSAEKKLGWVPSCDAGEQCAVRKGARIGKLCNCPRGTSCNFYIL

1Loculatus IEALQEVLEKLKNKRMPSAEKKLGWVPSCDAGEQCAVRKGARIGKLCNCPRGTSCNFYIL

*****:***.*.. * .: . :..*:..: **:**.:*.* ** *. * * : ::

1aSsenegalensis KCV

3Csemilaevis KCL

6Drerio KCL

2Dclupeoides KCL

2Cchanos KCL

1Dclupeoides KCS

1Drerio KCL

1Cchanos KCL

5Olatipes KCL

1Xmaculatus QCA

4Aocellaris KCL

5Hburtoni KCL

4Mzebra KCL

2bOniloticus KCL

6Aanguilla KCL

1Omordax KCL

2Ssalar KCL

3Ssalar KCL

6Elucius KCL

3Salpinus KCL

1Aanguilla KCL

1Sformosus KCL

9Hhuso KCL

10Hhuso KCL

2Lchalumnae KCL

5Ecalabaricus KCL

1Loculatus KCL

:*

3a.muscle

4Bbufo VEAMEELLEKF-QDRY---PVYQKRAQIPLCDIGERCAVKQGPRIGKLCDCSRGSSCNSF

3Xtropicalis VEAMEELLGKF-QDRYP--TYQKKA-QIPLCDIGERCAVKQGPRIGKLCDCSRGSSCNSF

3Municolor VEAMEELLGKF-QNKYP--SYQKKAAQIPMCDIGERCAVKQGPRIGKLCDCSRGAICNTF

4Acatesbeiana VEAMEELLGKS---------LYQKRAQIPMCDIGERCAVKQGPRIGKLCDCSRGSSCNTF

6Xtropicalis AAALEEMLDYN-QDKGI--RLQRRVGQLPWCDVGGRCAMKRGPRIGKLCDCLRGTSCNSF

5Acatesbeiana AVALGELLDYN---QDRGLSLEKKASQLPRCDVGERCAMKHGPRIGKLCDCLRGASCSSF

5Bbufo AVALGEMLEYN--DPDGGVALEKKAVQVPRCDVGERCALKHGPRIGKLCDCLRGASCNSF

5Lchalumnae VEAINDILEND-HDRPI--SVEKKASQIPRCDVGERCAVKYGPRIGKLCDCLRGAACNTF

5Loculatus AEALEGLLDES-QDNRV--SVDKK-SLIPRCDVGERCAVKHGPRIGKLCDCLRGAACNTF

6Ecalabaricus AEVLEGLLENN-QDNAI--AVDKKASQIPRCDVGERCAMKHGPRIGKLCDCLRGAACNTF

7Hhuso VDALEGLLENS--SDTI--AVEKKANQIPRCDVGERCALKYGPRIGKLCDCLRGAACNTF

8Hhuso VDALEGLLENS--SDTI--AVEKKASQIPRCDVGERCALKYGPRIGKLCDCLRGAACNTF

3aSsenegalensis VEALQGVLGDS---DTLSLSVEKKASVIPRCDVGERCAMKHGPRIGRLCDCLRGTACNTF

4Csemilaevis QEALQSLLSDS---NAASLSVEKKAGVIPRCDVGERCAMKHGPRIGRLCDCLRGTACNTF

Amexicanus AEALEDMLDGD-EDNRI--QLEKKASVIPRCDVGERCALKHGPRIGRLCDCMRGTACNTF

5Dclupeoides AEALEDFLEGE-QDNRI--SVEKKASVIPRCDVGERCAMKHGPRIGRLCDCMRGTACNTF

4Drerio AEALDELLDGE-QDNRI--SLEKKASVIPRCDVGERCAMKHGPRIGRLCDCMRGTACNTF

6Sformosus AEALGGLLEGE-QDHRI--FLEKKASVIPRCDVGERCAMKHGPRIGRLCDCLRGTACNSF

5Aanguilla AEALEGLLDGT-QDNRI--TLEKKASVIPRCDVGERCAMKHGPRIGRLCDCLRGTACNSF

6Cchanos VDALEGLLEGD-QDNRI--SLEKKASVIPRCDVGERCAMKHGPRIGRLCDCLRGTACNTF

5Omordax ADALEGLLESG-QENSIGLSVEKKASVIPRCDVGERCAMKHGPRIGRLCDCMRGTACNTF

8Ssalar ADALERLLEGVQQDNRIGLSVEKKASLIPRCDVGERCAMKHGPRIGRLCDCLRGTACNTF

1Salpinus ADALERLLEGVQQDNRIGLSVEKKASLIPRCDVGERCAMKHGPRIGRLCDCLRGTACNTF

3Elucius ADALEGLLEDKQQDNMIGLSVEKKASLIPRCDVGERCAMKHGPRIGRLCDCLRGTACNTF

6Ssalar ADALEGLLEGGQQDNMIGLSVEKKASLIPRCDVGERCAMKHGPRIGRLCDCLRGTACNTF

2Salpinus ADALEGLLEGGQQDNMIGLSVEKKASLIPRCDVGERCAMKHGPRIGRLCDCLRGTACNTF

2Hcomes AEVLQGFLDEA-EGGGAGVSREKKASFIPRCDVGERCAMKHGPRIGRLCDCLRGTACNTF

S3bSquinqueradiata AEALQGFLDEA--DSRVGLSVEKKASVIPRCDVGERCAMKHGPRIGRLCDCLRGTACNTF

3Sdumerili AEALQGFLDEA--DSRVGLSVEKKASVIPRCDVGERCAMKHGPRIGRLCDCLRGTACNTF

2Xmaculatus AEALQGLLDEA--DSRVGLSVEKKASVIPRCDVGERCAMKHGPRIGRLCDCLRGTACNTF

7Aocellaris AEALQGLLDEA--DSRVGLSVEKKASVIPRCDVGERCAMKHGPRIGRLCDCLRGTACNTF

3aSaurata AEALQGFLDEA--DSSVGLSVEKKASVIPRCDVGERCAMKHGPRIGRLCDCLRGTACNTF

2Hburtoni AEALQGLLDEA--DSSAGLSVEKKASVIPRCDVGERCAMKHGPRIGRLCDCLRGTACNTF

3aOniloticus AEALQGLLDEA--DSSAGLSVEKKASVIPRCDVGERCAMKHGPRIGRLCDCLRGTACNTF

2Mzebra AEALQGLLDEA--DSSAGLSVEKKASVIPRCDVGERCAMKHGPRIGRLCDCLRGTACNTF

1Olatipes AEALQGLLDEA--DSSVGLSVEKKASVIPRCDVGERCAMKHGPRIGRLCDCLRGTACNTF

.: .* .. :* **:* ***:* *****.**** **: *.:*

4Bbufo LLKCI

3Xtropicalis LLKCI

3Municolor LLKCI

4Acatesbeiana LLKCI

6Xtropicalis LLRCY

5Acatesbeiana MLRCY

5Bbufo MLRCY

5Lchalumnae LLRCY

5Loculatus LLRCY

6Ecalabaricus LLRCY

7Hhuso LLRCY

8Hhuso LLRCY

3aSsenegalensis FLRCY

4Csemilaevis FLRCY

Amexicanus FLRCY

5Dclupeoides FLRCY

4Drerio FLRCY

6Sformosus FLRCY

5Aanguilla FLRCY

6Cchanos FLRCY

5Omordax FLRCY

8Ssalar FLRCY

1Salpinus FLRCY

3Elucius FLRCY

6Ssalar FLRCY

2Salpinus FLRCY

2Hcomes FLRCY

S3bSquinqueradiata FLRCY

3Sdumerili FLRCY

2Xmaculatus FLRCY

7Aocellaris FLRCY

3aSaurata FLRCY

2Hburtoni FLRCY

3aOniloticus FLRCY

2Mzebra FLRCY

1Olatipes FLRCY

:*.*

3b_clean.muscle

4Bbufo VEAMEELLEKF-QDRY---PVYQKRAQIPLCDIGERCAVKQGPRIGKLCDCSRGSSCNSF

3Xtropicalis VEAMEELLGKF-QDRYP--TYQKKA-QIPLCDIGERCAVKQGPRIGKLCDCSRGSSCNSF

3Municolor VEAMEELLGKF-QNKYP--SYQKKAAQIPMCDIGERCAVKQGPRIGKLCDCSRGAICNTF

4Acatesbeiana VEAMEELLGKS---------LYQKRAQIPMCDIGERCAVKQGPRIGKLCDCSRGSSCNTF

6Xtropicalis AAALEEMLDYN-QDKGI--RLQRRVGQLPWCDVGGRCAMKRGPRIGKLCDCLRGTSCNSF

5Acatesbeiana AVALGELLDYN---QDRGLSLEKKASQLPRCDVGERCAMKHGPRIGKLCDCLRGASCSSF

5Bbufo AVALGEMLEYN--DPDGGVALEKKAVQVPRCDVGERCALKHGPRIGKLCDCLRGASCNSF

5Lchalumnae VEAINDILEND-HDRPI--SVEKKASQIPRCDVGERCAVKYGPRIGKLCDCLRGAACNTF

5Loculatus AEALEGLLDES-QDNRV--SVDKK-SLIPRCDVGERCAVKHGPRIGKLCDCLRGAACNTF

6Ecalabaricus AEVLEGLLENN-QDNAI--AVDKKASQIPRCDVGERCAMKHGPRIGKLCDCLRGAACNTF

7Hhuso VDALEGLLENS--SDTI--AVEKKANQIPRCDVGERCALKYGPRIGKLCDCLRGAACNTF

8Hhuso VDALEGLLENS--SDTI--AVEKKASQIPRCDVGERCALKYGPRIGKLCDCLRGAACNTF

3aSsenegalensis VEALQGVLGDS---DTLSLSVEKKASVIPRCDVGERCAMKHGPRIGRLCDCLRGTACNTF

4Csemilaevis QEALQSLLSDS---NAASLSVEKKAGVIPRCDVGERCAMKHGPRIGRLCDCLRGTACNTF

Amexicanus AEALEDMLDGD-EDNRI--QLEKKASVIPRCDVGERCALKHGPRIGRLCDCMRGTACNTF

5Dclupeoides AEALEDFLEGE-QDNRI--SVEKKASVIPRCDVGERCAMKHGPRIGRLCDCMRGTACNTF

4Drerio AEALDELLDGE-QDNRI--SLEKKASVIPRCDVGERCAMKHGPRIGRLCDCMRGTACNTF

6Sformosus AEALGGLLEGE-QDHRI--FLEKKASVIPRCDVGERCAMKHGPRIGRLCDCLRGTACNSF

5Aanguilla AEALEGLLDGT-QDNRI--TLEKKASVIPRCDVGERCAMKHGPRIGRLCDCLRGTACNSF

6Cchanos VDALEGLLEGD-QDNRI--SLEKKASVIPRCDVGERCAMKHGPRIGRLCDCLRGTACNTF

5Omordax ADALEGLLESG-QENSIGLSVEKKASVIPRCDVGERCAMKHGPRIGRLCDCMRGTACNTF

8Ssalar ADALERLLEGVQQDNRIGLSVEKKASLIPRCDVGERCAMKHGPRIGRLCDCLRGTACNTF

1Salpinus ADALERLLEGVQQDNRIGLSVEKKASLIPRCDVGERCAMKHGPRIGRLCDCLRGTACNTF

3Elucius ADALEGLLEDKQQDNMIGLSVEKKASLIPRCDVGERCAMKHGPRIGRLCDCLRGTACNTF

6Ssalar ADALEGLLEGGQQDNMIGLSVEKKASLIPRCDVGERCAMKHGPRIGRLCDCLRGTACNTF

2Salpinus ADALEGLLEGGQQDNMIGLSVEKKASLIPRCDVGERCAMKHGPRIGRLCDCLRGTACNTF

2Hcomes AEVLQGFLDEA-EGGGAGVSREKKASFIPRCDVGERCAMKHGPRIGRLCDCLRGTACNTF

S3bSquinqueradiata AEALQGFLDEA--DSRVGLSVEKKASVIPRCDVGERCAMKHGPRIGRLCDCLRGTACNTF

3Sdumerili AEALQGFLDEA--DSRVGLSVEKKASVIPRCDVGERCAMKHGPRIGRLCDCLRGTACNTF

2Xmaculatus AEALQGLLDEA--DSRVGLSVEKKASVIPRCDVGERCAMKHGPRIGRLCDCLRGTACNTF

7Aocellaris AEALQGLLDEA--DSRVGLSVEKKASVIPRCDVGERCAMKHGPRIGRLCDCLRGTACNTF

3aSaurata AEALQGFLDEA--DSSVGLSVEKKASVIPRCDVGERCAMKHGPRIGRLCDCLRGTACNTF

2Hburtoni AEALQGLLDEA--DSSAGLSVEKKASVIPRCDVGERCAMKHGPRIGRLCDCLRGTACNTF

3aOniloticus AEALQGLLDEA--DSSAGLSVEKKASVIPRCDVGERCAMKHGPRIGRLCDCLRGTACNTF

2Mzebra AEALQGLLDEA--DSSAGLSVEKKASVIPRCDVGERCAMKHGPRIGRLCDCLRGTACNTF

1Olatipes AEALQGLLDEA--DSSVGLSVEKKASVIPRCDVGERCAMKHGPRIGRLCDCLRGTACNTF

.: .* .. :* **:* ***:* *****.**** **: *.:*

4Bbufo LLKCI

3Xtropicalis LLKCI

3Municolor LLKCI

4Acatesbeiana LLKCI

6Xtropicalis LLRCY

5Acatesbeiana MLRCY

5Bbufo MLRCY

5Lchalumnae LLRCY

5Loculatus LLRCY

6Ecalabaricus LLRCY

7Hhuso LLRCY

8Hhuso LLRCY

3aSsenegalensis FLRCY

4Csemilaevis FLRCY

Amexicanus FLRCY

5Dclupeoides FLRCY

4Drerio FLRCY

6Sformosus FLRCY

5Aanguilla FLRCY

6Cchanos FLRCY

5Omordax FLRCY

8Ssalar FLRCY

1Salpinus FLRCY

3Elucius FLRCY

6Ssalar FLRCY

2Salpinus FLRCY

2Hcomes FLRCY

S3bSquinqueradiata FLRCY

3Sdumerili FLRCY

2Xmaculatus FLRCY

7Aocellaris FLRCY

3aSaurata FLRCY

2Hburtoni FLRCY

3aOniloticus FLRCY

2Mzebra FLRCY

1Olatipes FLRCY

:*.*
