# Supplementary material for: Cocaine- and amphetamine-regulated transcripts in two percomorphs: evolutionary conservation and energy-status dependent responses
Source: Front Endocrinol (Lausanne). 2026 Jun 30;17:1870522. doi: 10.3389/fendo.2026.1870522 (PMC13364575; doi:10.3389/fendo.2026.1870522)
Supplement: Supplementary file 3 [file DataSheet3.docx]

CLUSTAL W (1.81) multiple sequence alignment

3a1Xtropicalis AAALEEMLDYN--QDKG--IRLQRRVGQLPWC--DVGGRCAMKRGPRIGKLCDCLRGTSC

3a1Acatesbeiana AVALGELLDYN--QDRG--LSLEKKASQLPRC--DVGERCAMKHGPRIGKLCDCLRGASC

3a1Bbufo AVALGEMLEYN--DPDG-GVALEKKAVQVPRC--DVGERCALKHGPRIGKLCDCLRGASC

3aSaurata AEALQGFLDEA--DSSV-GLSVEKKASVIPRC--DVGERCAMKHGPRIGRLCDCLRGTAC

3aOniloticus AEALQGLLDEA--DSSA-GLSVEKKASVIPRC--DVGERCAMKHGPRIGRLCDCLRGTAC

3aOmordax ADALEGLLESG-QENSI-GLSVEKKASVIPRC--DVGERCAMKHGPRIGRLCDCMRGTAC

3a2Ssalar ADALERLLEGVQQDNRI-GLSVEKKASLIPRC--DVGERCAMKHGPRIGRLCDCLRGTAC

3a1Ssalar ADALEGLLEGGQQDNMI-GLSVEKKASLIPRC--DVGERCAMKHGPRIGRLCDCLRGTAC

3aDrerio AEALDELLDGE--QDNR--ISLEKKASVIPRC--DVGERCAMKHGPRIGRLCDCMRGTAC

3aSformosus AEALGGLLEGE--QDHR--IFLEKKASVIPRC--DVGERCAMKHGPRIGRLCDCLRGTAC

3aAanguilla AEALEGLLDGT--QDNR--ITLEKKASVIPRC--DVGERCAMKHGPRIGRLCDCLRGTAC

3aLchalumnae VEAINDILEN---DHDR-PISVEKKASQIPRC--DVGERCAVKYGPRIGKLCDCLRGAAC

3a1Hhuso VDALEGLLENS--SDTI---AVEKKANQIPRC--DVGERCALKYGPRIGKLCDCLRGAAC

3a2Hhuso VDALEGLLENS--SDTI---AVEKKASQIPRC--DVGERCALKYGPRIGKLCDCLRGAAC

3aLoculatus AEALEGLLDES--QDNR--VSVDKK-SLIPRC--DVGERCAVKHGPRIGKLCDCLRGAAC

3aEcalabaricus AEVLEGLLENN--QDNA--IAVDKKASQIPRC--DVGERCAMKHGPRIGKLCDCLRGAAC

3bDrerio VEAMTALLERY--QSHL--PSSEKR--AIPQC--ALGSRCAMRLGSRFGKLCECGRGSNC

3bSsalar VGAMEALIVKM--QSHL--PTNEKR-GMIPPC--GMGDRCALRHGPRIGKLCDCGRVSSC

3bAanguilla LEAMEDLLGKF--QSRL--PSTEKR-GSIPLC--GVGDRCAVRLGPRIGKLCDCAGRRNC

3Cmilli FEAVEEILGKL--HNAI-SPSYEKKAGQIPKC--DIGDRCAIKQGPRIGKLCDCARGTTC

3bSformosus VEALEEILGKF--ENRV--P--EKR-GSIPTC--GRGERCAVKLGPRIGKLCDCGRGSHC

3bLchalumnae VEAMEELLGKF--QNRY--PTYEKKGGQIPLC--AIGERCAVKQGPRIGKLCDCSRGSSC

3a2Bbufo VEAMEELLEKF--QDRY--PVYQKR-AQIPLC--DIGERCAVKQGPRIGKLCDCSRGSSC

3a2Xtropicalis VEAMEELLGKF--QDRY--PTYQKK-AQIPLC--DIGERCAVKQGPRIGKLCDCSRGSSC

3aMunicolor VEAMEELLGKF--QNKY--PSYQKKAAQIPMC--DIGERCAVKQGPRIGKLCDCSRGAIC

3a2Acatesbeiana VEAMEELLGKS---------LYQKR-AQIPMC--DIGERCAVKQGPRIGKLCDCSRGSSC

3bEcalabaricus VETMEELLGKF--QSRL--PSYEKKGGTIPLC--DVGDRCAVKLGPRIGKLCNCARGSSC

3bLoculatus VEAMEELLGKV--QSRF--PSYEKKAATIPMC--DVGDRCALRQGPRIGKLCDCARGSIC

3b2Hhuso VEAMEELLGKF--QSRF--PSYEKKAGTIPLC--DVGDRCAVKQGPRIGKLCDCARGSTC

3b1Hhuso VEAMEELLGKF--QSRF--PSYEKKAGTIPLC--DVGDRCAVKQGPRIGKLCDCARGSTC

3bOmordax MDVMETLLGKM---NHR-FPSTDKR-GSIPIC--GMRDRCAMRLGPRIGKLCDCGRGGNC

3bSaurata VEALEALLGRM--HSRT--GSTEKR-GSIPLC--GMGDRCAMKFGPRIGKLCDCGRGANC

3bOniloticus LEALDVLLGRN--HNQV--SSPEKR-GSIPLC--GLGNRCAMKYGPRIGKLCDCGRGANC

4Omordax INDLQGVLERL--KNKR-FLPHAKKHSLLPMC--DAGEQCALRKGARIGKLCDCQQPRAC

4Saurata INDLHEVLERL--QNNQ-FPALRKKHGYLPVC--DPGDQCALRRGSRIGKLCDCSPPRTC

4Oniloticus INDLHEVLERL--QNHP-FPVLRKKHGYLPVC--DPGEQCALRKGSRIGKLCDCSLPRTC

2aPmajor VEVLQEVLDKL--RTRE-PPALEKRLSWVPWC--EPREPCAVRRGARIGKLCSCPRGTSC

2aGgallus VEALQEVLEKL--RSRE-LPPTAKKPGRVPSC--HLGEPCAVRVGARYGKRCSCPPGTAC

1a2Sformosus LDVLRNVLEGL--QKKR-LSVLQRRYRRLPGC--NVGDFCSVKRGARHGQLCDCPRGSKC

1aEcalabaricus LGALQEVLEKL--QHRR-LSTWDKKFSRVPRC--AIGDYCSVKKGARFGKLCDCPQEASC

1aLoculatus LGALQHVLEKL--QNRR-LGTWEKKLSRLPQC--DIGDYCSVKKGARFGKLCDCPRGVKC

1aAanguilla LDVLHDVLEKL--RNRR-MAILERTHSRLPRC--SVGDFCSVKKGARFGQLCDCPRGSKC

1aOmordax LGVLHNVLEKL--QNRR-MASWERRQSRLPSC--NVGDFCTAKKGPRFGQLCDCPRGSKC

1a1Sformosus LDVLHNVLEKL--QNRR-MGGWERKTSRLPVC--YIGDFCSVKKGSRFGQLCDCPRGSKC

1aGjaponicus LEELQNVLEKL--QHKT-VSTWEKKFNLVPKC--SFGDLCAVKKGARIGKLCDCPRGSAC

1aPbivittatus LEELQEVLEKL--QHKK-VSPWEKKFNQVPKC--SFGDPCAIRKGARIGKLCDCPRRAAC

1aLchalumnae LNALQGVLEKL--QSKR-ILTWEKKFNQVPKC--SIGNFCAVKKGARIGRLCDCPRWTSC

1acpb LEELQDVLEKL--QSKR-ISTWEKKHNQVPKC--SIGQACAVKKGARIGRLCDCPRGATC

2bOniloticus IEALQEVLEKL--KGKQ-LPSSEKKLGWLAAC--DAGEQCAIRKASRIGKLCGCPGGTVC

2b2Drerio IEALQEVLEKL--KNKQ-LPQTGKKLSLLPSC--DAGEQCAIRKGARVGKLCSCPQGTSC

2acpb IDALQEVLEKL--KTER-LPSIEKKLGSVASC--DAGEPCAVRKGARIGRLCSCPRGTAC

2b1Drerio IEALQEVLEKL--RNKQ-IPAVEKKLGWVPSC--DAGEQCAVRKGSRFGKLCSCPGGTAC

2aSsalar FEALQEVLEKL--QSKQ-MPAYEKKLGWVPMCDADAGQQCAVRKGARIGKLCECPRGTSC

2aSaurata IDALQEVLEKL--RSKE-MP-LEKKLGWLPSC--DAGEPCAVRKGARIGTLCSCPRGTSC

2aOniloticus IDALQEVLEKL--RSKE-MP-LEKKHGWLPSC--DAGEPCAVRKGARIGTLCSCPRGTTC

2b1Aanguilla IEALQEVLEKL--KNQR-MPATEKKLGWVSSC--DAGEECALRKGARIGKLCSCPRGTSC

2aGjaponicus MEALQEVLEKL--RSSRLLPPLEKKLGWVPSC--DAGESCAVRKGSRIGKLCNCPRRTSC

2aMunicolor IEALQDVLKKL--ESKR-MPSLEKKLGWLPSC--DAGEQCAVRKGARIGKLCSCPRGTAC

2aXtropicalis IDALQEVLEKL--KSKR-ILPLDKKLGWVPSC--DAGEQCAVRKGARIGKLCNCPRGTAC

2aAcatesbeiana IEALQDVLEKL--KSKR-ILPLDKKLGWVPSC--DAGEQCAVRKGARIGKLCNCPRGTTC

2aBbufo IEALQEVLEKL--KSKK-ILPLDKKLGWVPSC--DAGEQCAVRKGARIGKLCNCPRGTSC

2b2bSsalar IEALQEVLEKL--KNKQ-MPLSEKKLGWLPSC--DAGEQCAVRKGARVGTLCGCPRGTTC

2b2aSsalar IEALQEVLEKL--KNKQ-MPLSEKKLGWLPSC--DAGEQCAVRKGARVGTLCGCPRGTTC

2bOmordax IEALQEVLEKL--KNKQ-MPSSEKKLGWLPSC--DAGEQCAVRKGARVGTLCGCPRGTSC

2bAanguilla IEALQEVLEKL--KNKQ-MPSAEKKLGWLPSC--DAGEQCAIRKGARIGQLCGCPRGTSC

2bSformosus IEALQEVLEKL--KNKQ-MPSAEKKLGWVPSC--DAGEQCAIRKGARIGKLCNCPRGTSC

2b1aHhuso IEALQEVLEKL--KSKR-LPSAEKKLGWVPSC--DAGEQCAVRKGSRIGKLCNCPRGTSC

2b1bHhuso IEALQEVLEKL--KSKR-LPSAEKKLGWVPSC--DAGEQCAVRKGSRIGKLCNCPRGTSC

2bLchalumnae IEALQEVLEKL--KNKR-VPSAEKKLGWVPSC--DAGEQCAVRKGARIGKLCNCPRGTSC

2bEcalabaricus IEALQEVLEKL--KSKR-MPSAEKKLGWVPSC--DAGEQCAVRKGARIGKLCNCPRGTSC

2bLoculatus IEALQEVLEKL--KNKR-MPSAEKKLGWVPSC--DAGEQCAVRKGARIGKLCNCPRGTSC

1Cmilli LGALQEVLEKL--QSKR-LPTWEKKFGQLPLC--DIGEQCAVRKGARIGKLCDCPRSTGC

1b1cbp LGALQEALEKL--QKKR-IPPWGKKLGQVPAC--DVGELCAVRKASRIGKLCNCPRGATC

1b1Oniloticus LGALHEVLERL--QTKR-INPWEKKYGQVPSC--DLGEYCAIRKGSRIGKMCDCPRGAFC

1b1Saurata LGALHEVLEKL--QTKR-INPWEKKYGQVPSC--DLGEHCAVRKGSRIGKMCDCPRGAFC

1b1bDrerio LGALQEVLEKL--QTKR-IPPWEKKFGQVPMC--DLGEQCAIRKGSRIGKMCDCPRGALC

1b1aDrerio LGALHDVLEKL--QSKR-ISLWEKKFGRVPTC--DVGEQCAIRKGSRIGKMCDCPRGAFC

1b2bSsalar LGALHDVLKKL--QTKR-LPFWEKKFGQVPTC--DVGEQCAVRKGARIGKMCDCPRGAFC

1b2aSsalar LGALHDVLKKL--QTKR-LPFWEKKFGQVPTC--DVGEQCAVRKGARIGKMCDCPRGAFC

1b1Omordax LGALHDVLEKL--QTKR-ISPWEKKFGQVPTC--DMGEHCAVRKGARIGKMCDCPRGAFC

1b1bSsalar LGALHEVLKKL--QTKR-INPWEKKFGQVPTC--DVGDHCAVRKGARIGKMCDCPRGAFC

1b1aSsalar LGALHEVLKKL--QTKR-INPWEKKFGQVPTC--DVGDHCAVRKGARIGKMCDCPRGAFC

1b4Xtropicalis LGALQEVLEKL--QNKR-IPSWEKKFGQVPVC--DVGEQCAVRKASRIGKLCNCPRGAVC

1bBbufo LGALQEVLEKL--QSKR-VPLWEKKFGQVPVC--DMGEQCAVRKASRIGKLCNCPRGSVC

1bMunicolor LGALQEVLEKL--QSKR-VPVWEKKFGQVPTC--DIGEQCAVRKASRIGKLCNCPRGAVC

1bAcatesbeiana LGALQEVLEKL--QSKR-VPAWEKKFGQVPVC--DVGEQCAVRKASRIGKLCNCPRGAVC

1bLchalumnae VRALQEVLEKL--QSKR-VPTWEKKFGQVPMC--DVGEQCAVRKGARIGKLCDCPRGSIC

1b1aHhuso LGALQEVLEKL--QSKR-VPAWEKKFGQVPTC--DVREQCAVRKGARIGKLCDCPRGAIC

1b1bHhuso LGALQEVLEKL--QSKR-VPAWEKKFGQVPTC--DVREQCAVRKGARIGKLCDCPRGAIC

1bEcalabaricus LGALQEVLEKL--QSKR-VPAWEKKFGQVPTC--DVGEQCAVRKGARIGKLCDCPRGAIC

1b2Sformosus LGALQEVLEKL--QTKR-IPPWEKKFGQVPTC--DVGKQCAVRKGARIGKMCDCPRGTFC

1b2Aanguilla LGALQDVLEKL--QKKR-IPSWEKKFGQVPTC--DVGEQCAVRKGARIGKMCDCPRRAFC

1b1Aanguilla LGALQEVLEKL--QSKR-ISPWEKKFGQVPTC--DVGEQCAVRKGSRIGKMCDCPRGAFC

1b1Sformosus LGALQEVLEKL--QSKR-ISPWEKKFGQVPTC--DVGEQCAIRKGARIGKMCDCPRGAFC

1b1Loculatus LGALQEVLEKL--QSKR-IPTWEKKFGQVPTC--DVGEQCAVRKGARIGKMCDCPRGAFC

1b2Omordax LGALQDVLEKL--QTKR-LSMWEKKFGQVPTC--DVGEQCAVRKGARIGKMCDCPRGAFC

1b2Saurata LGALQEVLEKL--QAKR-LPMWEKKFGQVPTC--DVGEQCAVRKGARIGKMCDCPRGAFC

1b2Oniloticus LGALQEVLEKL--QAKR-LPLWEKKFGQVPTC--DIGEQCAVRKGARIGKMCDCPRGAFC

4Aanguilla ISALKGVLEKL--KNNR-FPLYGKKYGQLPMC--EAGERCALRKGARIGKLCDCPYRISC

4Municolor LEALQEVLEKL--KSKR-IPSYEKKYGQVPMC--EAGDQCAVRKGPRIGKLCDCPRRTSC

4Xtropicalis IDALQEVLEKL--KNKR-LPLFEKKYGQVPMC--DAGEQCAVRKGPRIGKLCDCPRRTSC

4Acatesbeiana IDALQEVLEKL--KNKR-LPLFEKKYGQVPMC--DAGEQCAVRKGPRIGKLCDCPRRTSC

4Bbufo INALQEVLEKL--KSKK-LPSFEKKYGQVPMC--DAGEQCAVRKGARIGRLCDCPRRTSC

4Loculatus IEALQEVLEKL--KNKG-MPFYGKKYGQLPMC--EAGEQCALRKGARIGKLCDCPRATSC

4Ecalabaricus IDALQEVLEKL--KNKR-IPYYEKKIGQLPMC--DAGDQCAVRKGARIGKLCDCPRGTSC

4bHhuso IEALQEVLEKL--KSKR-MPYYEKKYGQLPMC--DAGEQCALRKGARIGKLCDCPRGTSC

4aHhuso IEALQEVLEKL--KSKR-MPYYEKKYGQLPMC--DAGEQCALRKGARIGKLCDCPRGTSC

MLchalumnae IEALQEVLEKL--KSKR-IPVYEKKYNQVPMC--DAGEQCALRKGSRIGKLCDCPRGTSC

Sharrisii IGALQEVLKKL--KSKR-IRIYEKKYGQVPKC--DAGEQCAIRKGARIGKLCDCPRGTSC

Mdomestica IGALQEVLKKL--KSKR-IRIYEKKYGQVPKC--DAGEQCAIRKGARIGKLCDCPRGTSC

Dgliroides IGALQEVLKKL--KSKR-IRIYEKKYGQVPKC--DAGEQCAIRKGARIGKLCDCPRGTSC

MCmilli IEALQEVLEKL--KSKR-MPTYEKKFGMVPMC--DAGEQCAVRKGARIGKLCDCPRGTFC

Oanatinus IEALQEVLKKL--KSKR-IPVYEKKYSQVPMC--DAGEQCAVRKGARIGKLCDCPRGTAC

Tlatirostris IEALQEVLKKL--KSKR-IPIYEKKYGQVPMC--DAGEQCAVRKGARIGKLCDCPRATSC

Hsapiens IEALQEVLKKL--KSKR-VPIYEKKYGQVPMC--DAGEQCAVRKGARIGKLCDCPRGTSC

Btaurus IEALQEVLKKL--KSKR-IPIYEKKYGQVPMC--DAGEQCAVRKGARIGKLCDCPRGTSC

Mmusculus IEALQEVLKKL--KSKR-IPIYEKKYGQVPMC--DAGEQCAVRKGARIGKLCDCPRGTSC

Dnovemcintus IEALQEVLKKL--KSKR-IPIYEKKYGQVPMC--DAGEQCAVRKGARIGKLCDCPRGTSC

4Pmajor IEALQEVLEKL--KSKR-GPHYEKKFGQVPMC--DAGEQCAVRKGARIGKLCDCPRGTSC

4Ggallus IEALQEVLEKL--KSKR-VPHYEKKFGQVPMC--DAGEQCAVRKGARIGKLCDCPRGTSC

Mcpb IEALQEVLEKL--KSKR-IPVYEKKFGQVPMC--DAGEQCAVRKGARIGKLCDCPRGTSC

4Pbivittatus IEALQEVLEKL--KSKR-LPHYEKKYGQVPMC--DAGEQCAVRKGARIGKLCDCPRGTSC

4Gjaponicus IEALQEVLEKL--KSKR-LPHYEKKYGQVPMC--DAGEQCAVRKGARIGKLCDCPRGTSC

4anole IEALQEVLEKL--KSKR-LPLYEKKYGQVPMC--DAGEQCALRKGARIGKLCDCPRGTSC

1aBbufo LLELQDVLYKL--QSKR-SPAWESKYIQVPKC--IMGDACAVKRGARIGKLCDCPPWSTC

1aAcatesbeiana LLELQDVLEKL--QSKR-GILWESKLNQMPKC-LHHGDACAVKRGLRIGKLCDCPRRSVC

1a5Xtropicalis LTELQDVLEKL--QSKR-ILSWESKLNQVPKC--TLGDVCAVKRGARIGKLCDCPRRSNC

: : :. * *: . . * * * * *

3a1Xtropicalis NSFLLRCY--------

3a1Acatesbeiana SSFMLRCY--------

3a1Bbufo NSFMLRCY--------

3aSaurata NTFFLRCY--------

3aOniloticus NTFFLRCY--------

3aOmordax NTFFLRCY--------

3a2Ssalar NTFFLRCY--------

3a1Ssalar NTFFLRCY--------

3aDrerio NTFFLRCY--------

3aSformosus NSFFLRCY--------

3aAanguilla NSFFLRCY--------

3aLchalumnae NTFLLRCY--------

3a1Hhuso NTFLLRCY--------

3a2Hhuso NTFLLRCY--------

3aLoculatus NTFLLRCY--------

3aEcalabaricus NTFLLRCY--------

3bDrerio NSFLLKCI--------

3bSsalar NSFLLKCL--------

3bAanguilla NSFLLKCI--------

3Cmilli NSFLLKCI--------

3bSformosus NSFLLKCI--------

3bLchalumnae NSFLLKCI--------

3a2Bbufo NSFLLKCI--------

3a2Xtropicalis NSFLLKCI--------

3aMunicolor NTFLLKCI--------

3a2Acatesbeiana NTFLLKCI--------

3bEcalabaricus NSFLLKCI--------

3bLoculatus NSFLLKCI--------

3b2Hhuso NSFLLKCI--------

3b1Hhuso NSFLLKCI--------

3bOmordax NSYLLKCI--------

3bSaurata NSYLLKCI--------

3bOniloticus NSYLLKCI--------

4Omordax SSFMLRCL--------

4Saurata NSFLHRCL--------

4Oniloticus NSFLHRCL--------

2aPmajor NLFILKCS--------

2aGgallus NLYVLRCS--------

1a2Sformosus NYFFLKCL--------

1aEcalabaricus NFFFLKCL--------

1aLoculatus NFFFLKCL--------

1aAanguilla NYFFLKCLTPPALWAP

1aOmordax NHFFLKCL--------

1a1Sformosus NFFFLKCL--------

1aGjaponicus NTFLLKCL--------

1aPbivittatus NAFLLKCL--------

1aLchalumnae NSFLLKCL--------

1acpb NTFLLKCL--------

2bOniloticus NFSVLKCL--------

2b2Drerio HFFILKCL--------

2acpb NFYILKCL--------

2b1Drerio SFSILKCL--------

2aSsalar NFSILKCF--------

2aSaurata NFYVLKCL--------

2aOniloticus NFYVLKCL--------

2b1Aanguilla NFSILKCL--------

2aGjaponicus NMYILKCL--------

2aMunicolor NFYILKCL--------

2aXtropicalis NFYILKCL--------

2aAcatesbeiana NFYILKCL--------

2aBbufo NFYILKCL--------

2b2bSsalar NFYVLKCL--------

2b2aSsalar NFYVLKCL--------

2bOmordax NFYVLKCL--------

2bAanguilla NFYILKCL--------

2bSformosus NFSILKCL--------

2b1aHhuso NFYILKCL--------

2b1bHhuso NFYILKCL--------

2bLchalumnae NFYILKCL--------

2bEcalabaricus NFYILKCL--------

2bLoculatus NFYILKCL--------

1Cmilli NFFLLKCL--------

1b1cbp NFFLLKCL--------

1b1Oniloticus NFFLLKCL--------

1b1Saurata NFFLLKCL--------

1b1bDrerio NFFLLKCL--------

1b1aDrerio NYFLLKCL--------

1b2bSsalar NSYLLKCL--------

1b2aSsalar NSYLLKCL--------

1b1Omordax NFFLLKCL--------

1b1bSsalar NFFLLKCL--------

1b1aSsalar NFFLLKCL--------

1b4Xtropicalis NFFLLKCL--------

1bBbufo NFFLLKCL--------

1bMunicolor NFFLLKCL--------

1bAcatesbeiana NFFLLKCL--------

1bLchalumnae NFFLLKCL--------

1b1aHhuso NFFLLKCL--------

1b1bHhuso NFFLLKCL--------

1bEcalabaricus NFFLLKCL--------

1b2Sformosus NFFLLKCL--------

1b2Aanguilla NFFLLKCL--------

1b1Aanguilla NFFLLKCL--------

1b1Sformosus NFFLLKCL--------

1b1Loculatus NFFLLKCL--------

1b2Omordax NFFLLKCL--------

1b2Saurata NFFLLKCL--------

1b2Oniloticus NFFLLKCL--------

4Aanguilla NSFLLRCL--------

4Municolor NTYLLKCL--------

4Xtropicalis NTFLLKCL--------

4Acatesbeiana NTFLLKCL--------

4Bbufo NTFLLKCL--------

4Loculatus NSFLLKCL--------

4Ecalabaricus NSFLLKCL--------

4bHhuso NSFLLRCL--------

4aHhuso NSFLLRCL--------

MLchalumnae NSFLLKCL--------

Sharrisii NSFLLKCL--------

Mdomestica NSFLLKCL--------

Dgliroides NSFLLKCL--------

MCmilli NSFLLKCL--------

Oanatinus NSFLLKCL--------

Tlatirostris NSFLLKCL--------

Hsapiens NSFLLKCL--------

Btaurus NSFLLKCL--------

Mmusculus NSFLLKCL--------

Dnovemcintus NSFLLKCL--------

4Pmajor NSFLLKCL--------

4Ggallus NSFLLKCL--------

Mcpb NSFLLKCL--------

4Pbivittatus NTFLLKCL--------

4Gjaponicus NTFLLKCL--------

4anole NTFLLKCL--------

1aBbufo SLFFMRCL--------

1aAcatesbeiana NQFFLRCL--------

1a5Xtropicalis NYYFLRCL--------

. .*
