# Supplementary material for: Cocaine- and amphetamine-regulated transcripts in two percomorphs: evolutionary conservation and energy-status dependent responses
Source: Front Endocrinol (Lausanne). 2026 Jun 30;17:1870522. doi: 10.3389/fendo.2026.1870522 (PMC13364575; doi:10.3389/fendo.2026.1870522)
Supplement: Supplementary file 4 [file DataSheet4.docx]

# Supplemental Figures and Tables


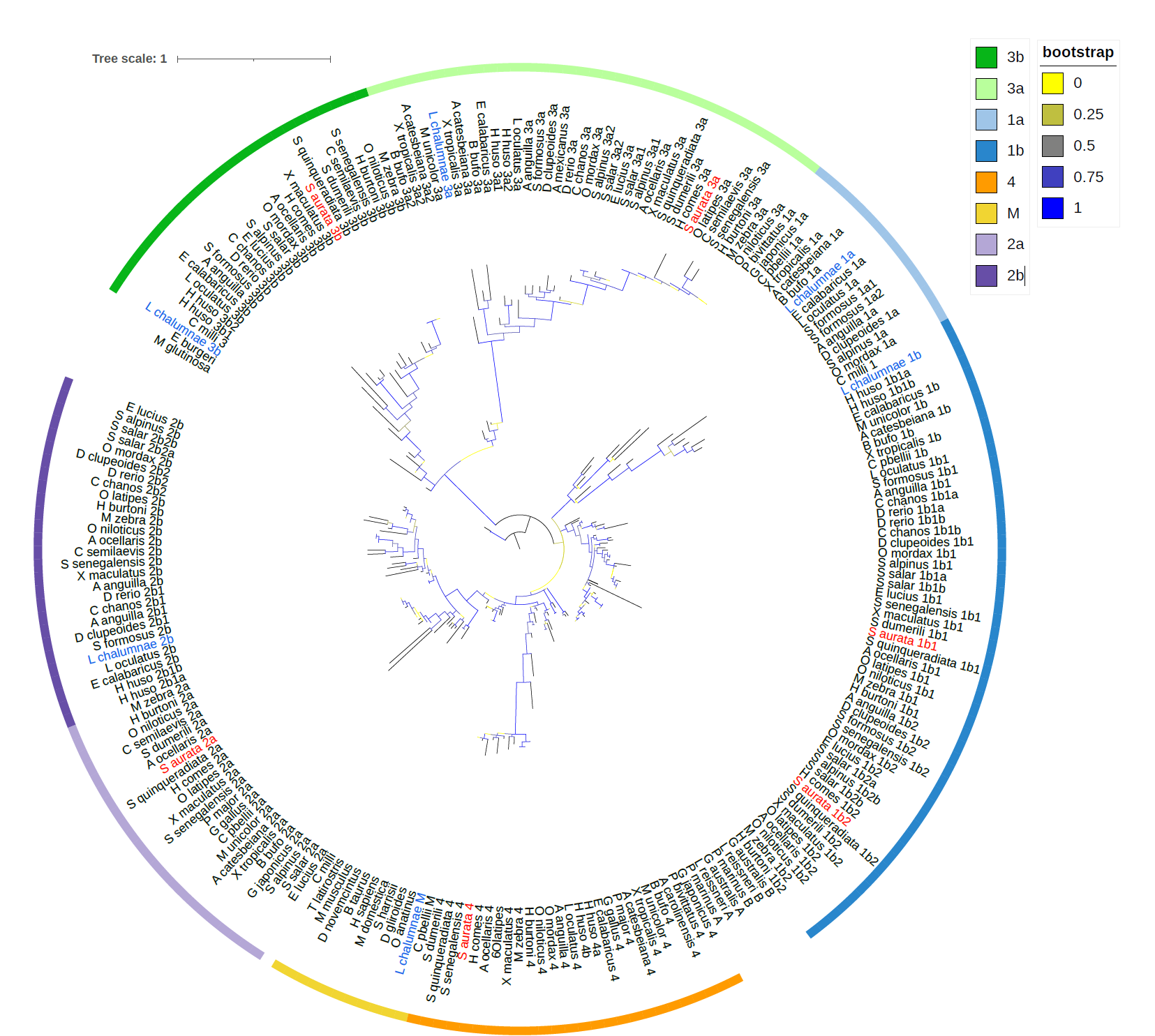


**Supplemental Figure 1**. Phylogenetic tree of tilapia and seabream carts that shows clustering into Four clades. The accession numbers of the sequences used in this analysis are included in Supplemental Tables 1 and 2. Complete parameters of the PhyML run are detailed in Supplemental Table 4, and the alignment in Supplemental file 1. The tree was rooted using the hagfish branch using iTol


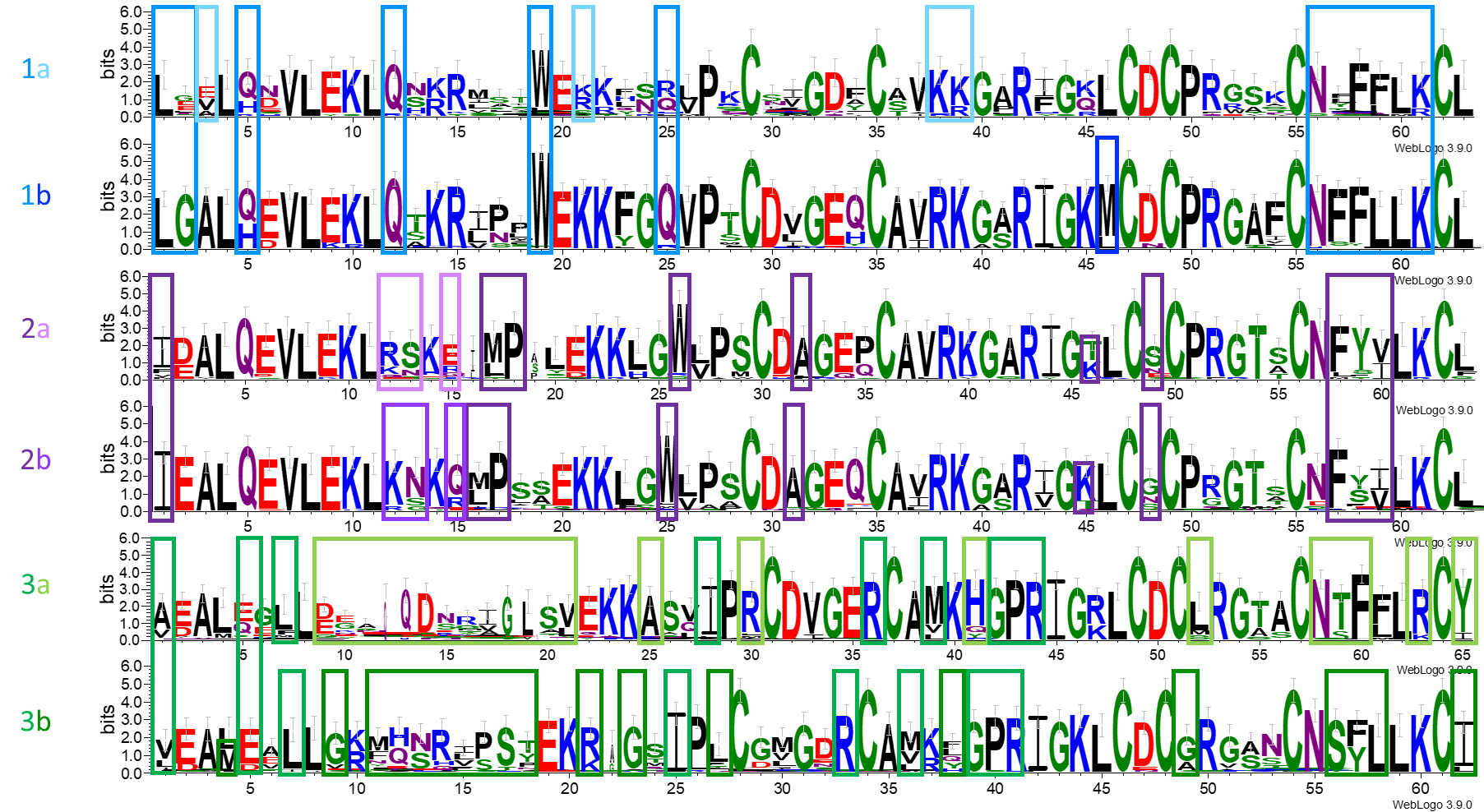


**Supplemental Figure 2**. Sequence logos for the three major groups and subgroups with differential positions highlighted. Each group has a mid color (group 1 blue, group 2 purple, group 3 green) to represent positions that typify the whole group. For each group, the lighter shade is a position unique to the a subclade, and a darker shade to the b subclade. logos were generated using Weblogo 3.9.0. Sequences included in this analysis are detailed in Supplemental file 3.

**Supplemental Figure 3**. Predicted 3D ribbon models of the bioactive form of tilapia carts: (A) human CART, (B) oncart1b1, (C) oncart1b2, (D) oncart2a, (E) oncart2b, (F) oncart3a, (G) oncart3a, and (H) oncart4


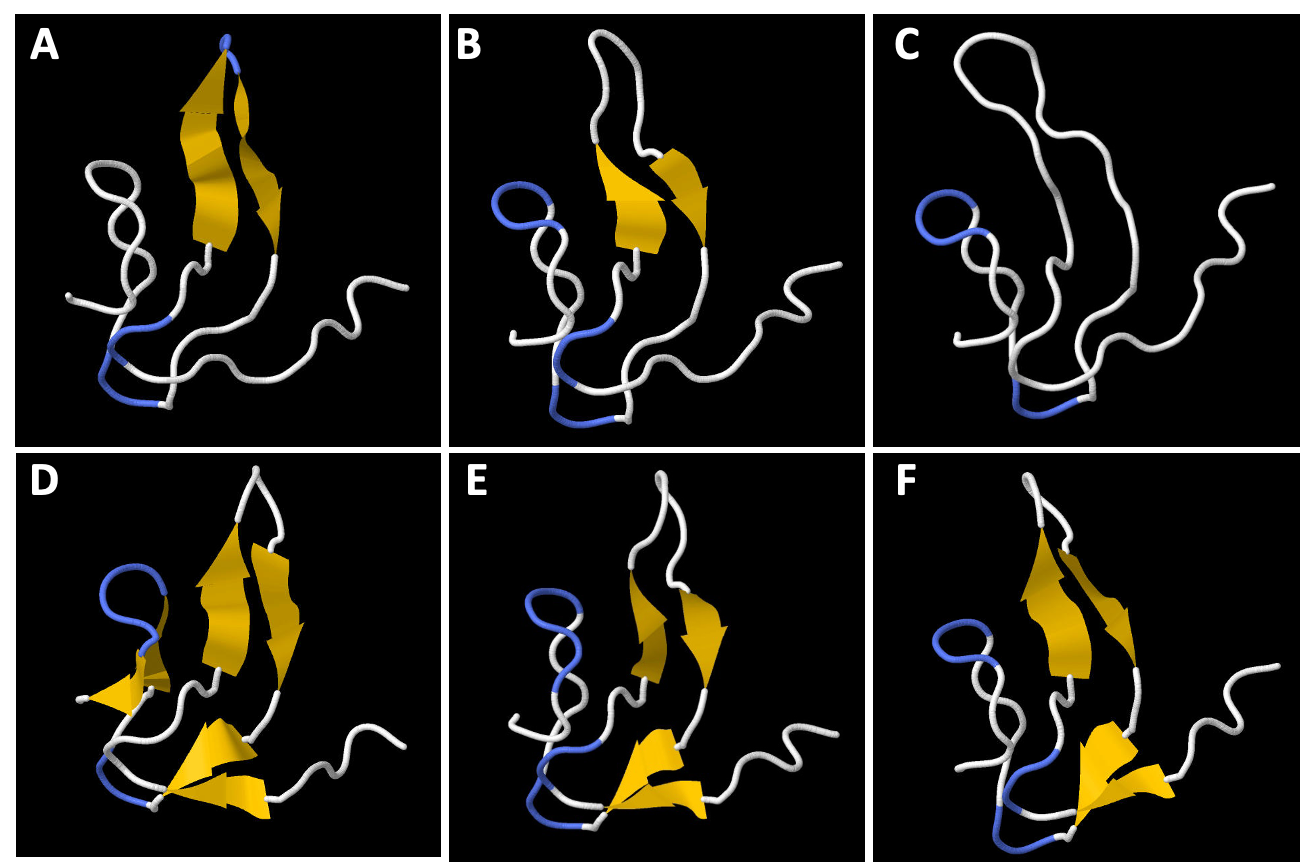


**Supplemental Figure 4**. Predicted 3D models of the bioactive form of seabream carts: (A) sacart1b1, (B) sacart1b2, (C) sacart2a, (D) sacart3a, (E) sacart3b, (F) sacart4.

**Supplemental Table 3.** Complete parameters used for the PhyML run of Figure 2

~~~~~~~~~~~~~~~~~~~~~~~~~~~~~~~~~~~~~~~~~~~~~~~~~~~~~~~~~~~~

                     Starting SMS v2.1

~~~~~~~~~~~~~~~~~~~~~~~~~~~~~~~~~~~~~~~~~~~~~~~~~~~~~~~~~~~~

Input alignment    : cart_exons_small_phy

Data type          : Protein

Number of taxa     : 127

Number of sites    : 76

Number of branches : 251

Criterion          : BIC

~~~~~~~~~~~~~~~~~~~~~~~~~~~~~~~~~~~~~~~~~~~~~~~~~~~~~~~~~~~~

Step 1 : Set a fixed topology

BIC=10012.08348

~~~~~~~~~~~~~~~~~~~~~~~~~~~~~~~~~~~~~~~~~~~~~~~~~~~~~~~~~~~~

Step 2 : Select the best decoration

BIC=9616.03239

decoration : '+G+I'

~~~~~~~~~~~~~~~~~~~~~~~~~~~~~~~~~~~~~~~~~~~~~~~~~~~~~~~~~~~~

Step 3 : Select the best matrix

BIC=9458.39230

matrix : 'Q.plant'

~~~~~~~~~~~~~~~~~~~~~~~~~~~~~~~~~~~~~~~~~~~~~~~~~~~~~~~~~~~~

Step 4 : Select the best final decoration

BIC=9458.39026

decoration : '+G+I+F'

~~~~~~~~~~~~~~~~~~~~~~~~~~~~~~~~~~~~~~~~~~~~~~~~~~~~~~~~~~~~

Selected model

: Q.plant +G+I+F

~~~~~~~~~~~~~~~~~~~~~~~~~~~~~~~~~~~~~~~~~~~~~~~~~~~~~~~~~~~~

Substitution model

: Q.plant

Equilibrium frequencies

: ML optimized

Proportion of invariable sites

: estimated (0.123)

Number of substitution rate categories

: 4

Gamma shape parameter

: estimated (1.585)

**Supplemental Table 4.** Complete parameters used for the PhyML run of Supplemental Figure 1

~~~~~~~~~~~~~~~~~~~~~~~~~~~~~~~~~~~~~~~~~~~~~~~~~~~~~~~~~~~~

                     Starting SMS v2.1

~~~~~~~~~~~~~~~~~~~~~~~~~~~~~~~~~~~~~~~~~~~~~~~~~~~~~~~~~~~~

Input alignment    : cart_2exons_phy

Data type          : Protein

Number of taxa     : 224

Number of sites    : 76

Number of branches : 445

Criterion          : BIC

~~~~~~~~~~~~~~~~~~~~~~~~~~~~~~~~~~~~~~~~~~~~~~~~~~~~~~~~~~~~

Step 1 : Set a fixed topology

BIC=14114.68261

~~~~~~~~~~~~~~~~~~~~~~~~~~~~~~~~~~~~~~~~~~~~~~~~~~~~~~~~~~~~

Step 2 : Select the best decoration

BIC=13578.04152

decoration : '+G+I'

~~~~~~~~~~~~~~~~~~~~~~~~~~~~~~~~~~~~~~~~~~~~~~~~~~~~~~~~~~~~

Step 3 : Select the best matrix

BIC=13324.51547

matrix : 'Q.plant'

~~~~~~~~~~~~~~~~~~~~~~~~~~~~~~~~~~~~~~~~~~~~~~~~~~~~~~~~~~~~

Step 4 : Select the best final decoration

BIC=13324.50607

decoration : '+G+I+F'

~~~~~~~~~~~~~~~~~~~~~~~~~~~~~~~~~~~~~~~~~~~~~~~~~~~~~~~~~~~~

Selected model

: Q.plant +G+I+F

~~~~~~~~~~~~~~~~~~~~~~~~~~~~~~~~~~~~~~~~~~~~~~~~~~~~~~~~~~~~

Substitution model

: Q.plant

Equilibrium frequencies

: ML optimized

Proportion of invariable sites

: estimated (0.092)

Number of substitution rate categories

: 4

Gamma shape parameter

: estimated (1.491)

~~~~~~~~~~~~~~~~~~~~~~~~~~~~~~~~~~~~~~~~~~~~~~~~~~~~~~~~~~~~

**Supplemental Table 5**. C-scores of cart genes of Nile tilapia and gilthead seabream

| ***Gilthead seabream*** | **Gene** | **C-score** | ***Nile tilapia*** | **Gene** | **C-score** |
| --- | --- | --- | --- | --- | --- |
|  | *cart1b1* | 1.27 |  | *cart1b1* | 1.13 |
|  | *cart1b2* | 1.22 |  | *cart1b2* | 1.22 |
|  | *cart2a* | 1.16 |  | *cart2a* | 1.16 |
|  |  |  |  | *cart2b* | 1.13 |
|  | *cart3a* | 1.19 |  | *cart3a* | 1.1 |
|  | *cart3b* | 1.26 |  | *cart3b* | 1.32 |
|  | *cart4* | 1.15 |  | *cart4* | 1.12 |
